# The multilevel correlates, contributions, and consequences of leader humility in humanitarian aid work

**DOI:** 10.3389/fpsyg.2023.1188109

**Published:** 2023-12-13

**Authors:** Edward B. Davis, Kelly Barneche, Jamie D. Aten, Laura R. Shannonhouse, David C. Wang, Daryl R. Van Tongeren, Don E. Davis, Joshua N. Hook, Zhuo Job Chen, G. Tyler Lefevor, Stacey E. McElroy-Heltzel, Emilie L. Elick, Leif Van Grinsven, Ethan K. Lacey, Tyler R. Brandys, Philip K. Sarpong, Sophia A. Osteen, Kati Shepardson

**Affiliations:** ^1^School of Psychology, Counseling, and Family Therapy, Wheaton College, Wheaton, IL, United States; ^2^Medair, Ecublens, Switzerland; ^3^Humanitarian Disaster Institute, Wheaton College, Wheaton, IL, United States; ^4^Department of Counseling and Psychological Services, Georgia State University, Atlanta, GA, United States; ^5^School of Psychology and Marriage and Family Therapy, Fuller Theological Seminary, Pasadena, CA, United States; ^6^Psychology Department, Hope College, Holland, MI, United States; ^7^Psychology Department, University of North Texas, Denton, TX, United States; ^8^School of Nursing, University of North Carolina at Charlotte, Charlotte, NC, United States; ^9^Department of Psychology, Utah State University, Logan, UT, United States; ^10^Department of Psychological and Quantitative Foundations, University of Iowa, Iowa City, IA, United States

**Keywords:** humility, leadership, humanitarian aid, nonprofit organizations, personnel

## Abstract

**Objective:**

Leader humility has been linked to many positive outcomes but not examined in humanitarian aid work. Three studies examined the multilevel correlates, contributions, and consequences of leader humility in Medair—a large, multinational, faith-based aid organization. Study 1 examined correlates of leader humility in a sample of 308 workers and 167 leaders. Study 2 explored multilevel contributions of leader humility in 96 teams comprised of 189 workers. Study 3 utilized a subsample (50 workers, 34 leaders) to explore consequences of Time 1 leader and team humility on outcomes 6 months later.

**Method:**

Participants completed measures of humility (general, relational, team), leader and team attributions (e.g., effectiveness, cohesion, and growth-mindedness), organizational outcomes (e.g., job engagement and satisfaction; worker and team performance), and psychological outcomes (e.g., depression, anxiety, compassion satisfaction, and flourishing).

**Results:**

Leader and team humility contributed to multilevel positive attributions about leaders (as effective and impactful), teams (as cohesive, psychologically safe, and growth-minded), and oneself (as humble), and those attributions contributed to organizational and psychological outcomes. Teams’ shared attributions of their leader’s humility contributed to higher worker job satisfaction and team performance. Longitudinally, for workers and leaders, leader and team humility were associated with some positive organizational and psychological outcomes over time.

**Conclusion:**

In humanitarian organizations, leader humility seems to act as an attributional and motivational social contagion that affects aid personnel’s positive attributions about their leaders, teams, and themselves. In turn, these multilevel positive attributions contribute to several positive team, organizational, and psychological outcomes among workers and leaders.

## Introduction

Growing evidence indicates leader humility is associated with benefits across widespread organizational and applied contexts (Chandler et al., 2023). One context in which it may be particularly helpful is humanitarian work, because humility is valued in many aid organizations (especially faith-based nonprofits) but is regularly strained by how stressful, culturally complex, and unpredictably fluid the nature of humanitarian work is (Shannonhouse et al., 2019; Wang et al., 2021). Even so, this possibility has not yet been evaluated empirically. Therefore, the purpose of these studies is to examine the multilevel correlates, contributions, and consequences of leader humility in Medair—a large, multinational, faith-based humanitarian aid organization.

## Defining and conceptualizing humility

*General humility* is “an interpersonal characteristic that emerges in social contexts [and] connotes (a) a manifested willingness to view oneself accurately, (b) a displayed appreciation of others’ strengths and contributions, and (c) teachability, or openness to new ideas and feedback” (Owens et al., 2013, p. 1518). Humility also has many subtypes, including relational humility, cultural humility, and intellectual humility (see Worthington et al., 2017; Van Tongeren et al., 2019a, for reviews). Humility and its subtypes are typically conceptualized and measured from a personality perspective, usually using the HEXACO model (Lee and Ashton, 2008), VIA character strengths and virtues model (Peterson and Seligman, 2004), or biopsychosocial model of temperament and character (Cloninger et al., 1993; Moreira et al., 2021). As such, humility and its subtypes are usually viewed and measured as stable *traits*—enduring qualities that characterize internal and interpersonal responses across time and situations.

Within organizations, humility and its subtypes can be understood and measured at three levels of analysis: individual humility of leaders (Owens et al., 2013) and followers (Diao et al., 2019), team collective humility (Owens and Hekman, 2016), and broader organizational humility (Chandler et al., 2023). At each of these levels, humility and its subtypes can be viewed as traits that characterize an individual (leader or follower), a team culture, or an organizational culture.

In the current research, we focus on three types of humility: general humility, relational humility, and team collective humility. *Relational humility* is a trait in which an individual or group “(a) is interpersonally other-oriented rather than self-focused, marked by a lack of superiority; and (b) has an accurate view of self [that is] not too inflated or too low” (Davis et al., 2011, p. 226). *Team collective humility* refers to “a group tendency toward owning limitations and mistakes, appreciating group members’ strengths, and being teachable” (Owens and Hekman, 2016, p. 1089). Taken together, this study examines humility at the team level (team collective humility) and general and relational humility at the individual level (leaders and workers).

## Empirical research on leader humility outside a humanitarian context

Research on leader humility has accelerated in the past 25 years, as evidence of its benefits has accrued (see Appendix S1 and Chandler et al., 2023, for reviews). These benefits can be generally categorized into positive team, organizational, and psychological outcomes.

First, leader humility is linked to positive team outcomes. For example, research supports the *social contagion hypothesis* of leader humility (Owens and Hekman, 2016), which posits that “leader behavior can spread via social contagion to followers, producing an emergent state that ultimately affects team performance” (p. 1088). Leader humility particularly contributes to two emergent states—higher team collective humility (Owens and Hekman, 2016; Rego et al., 2017, 2019) and team *collective promotion focus* (growth-mindedness; Owens and Hekman, 2016; Li et al., 2019), defined as “a collective team focus on progressively striving toward achieving the team’s highest potential” (Owens and Hekman, 2016, p. 1089). Leader humility is also linked to more friendly and less conflictual relationships among team members (Chiu et al., 2022), as well as to higher team creativity (Wang et al., 2019), psychological safety (Rego et al., 2021), and spirituality (Naseer et al., 2020). Furthermore, it contributes to better team performance (Chiu et al., 2016; Rego et al., 2019) and team *psychological capital* (PsyCap; Rego et al., 2017, 2019), defined as a “team’s shared positive appraisal of their circumstances and probability for success under those circumstances based on their combined motivated effort and perseverance” (Peterson and Zhang, 2011, p. 134). In sum, these findings resonate with the *positive attribution hypothesis of leader humility* (Qin et al., 2020), which asserts that positive attributions of leader humility are what contribute to its associated positive outcomes at the individual and team level.

Second, leader humility is related to positive organizational outcomes such as better follower job performance (Wang et al., 2018; Diao et al., 2019), job engagement (Nguyen et al., 2020; Li et al., 2021), and job satisfaction (Ou et al., 2017; Zhong et al., 2020). It is also linked to lower turnover (Owens et al., 2013; Ou et al., 2017); higher prosocial behaviors among followers (Carnevale et al., 2019; Owens et al., 2019); and higher perceived organizational support (Yuan et al., 2018). Followers identify more strongly with leaders they think are humble (Carnevale et al., 2019), and they view humble leaders as more competent (Cojuharenco and Karelaia, 2020), effective (Owens et al., 2013), and impactful on their team (Rego et al., 2018).

Lastly, leader humility is linked to positive psychological outcomes. Consistent with the social contagion and positive attribution hypotheses, leader humility is related to higher levels of followers’ self-rated humility (Diao et al., 2019; Zhong et al., 2020), PsyCap virtues (hope, resilience, and optimism; Bin et al., 2021), and other virtues (empathy, gratitude, authenticity, etc.; Naseer et al., 2020; Oc et al., 2020). It is related to lower follower burnout (Zhong et al., 2020) and higher follower creativity (Wang et al., 2017). Moreover, it contributes to greater attachment security (Bharanitharan et al., 2019) and relational closeness (Mao et al., 2017) between followers and leaders. Additionally, leaders’ self-rated humility is related to higher levels of leader mental and spiritual health (Jankowski et al., 2019; Ruffing et al., 2021).

## The need for research on leader humility in a humanitarian aid context

As summarized in Appendix S1 (Supplementary Tables S1, S2; cf. Chandler et al., 2023), leader humility has been studied in many organizational contexts, including information technology (Carnevale et al., 2019), hospitality (Bin et al., 2021), the military (Swain and Korenman, 2018), healthcare (Owens et al., 2013, 2015), faith communities (Jankowski et al., 2019), and faith-based colleges (Krumrei-Mancuso, 2018). Only two peer-reviewed studies have examined leader humility in a humanitarian aid context (Wang et al., 2017; Shannonhouse et al., 2019). This dearth is surprising given that 365 million of the world’s 8 billion inhabitants (~1 in 22 people) presently need humanitarian aid (OCHA, 2023), which is an exponential rise from 65 million in 2012 (~1 in 100 people; OCHA, 2013) and 129 million in 2017 (~1 in 55 people; OCHA, 2017).

There are several reasons why leader humility is so vital and impactful in a humanitarian aid context. Three major reasons have been identified: (a) humility involves a certain quality of thinking, acting, and behaving that reflects virtuous moral character; (b) humility has positive multilevel consequences (for aid leaders, workers, teams, organizations, and beneficiaries); and (c) humility corresponds to an authentic representation of humanitarian aid work (Wang et al., 2021). After all, *humanitarian aid* refers to protection and “assistance intended to save lives, alleviate suffering, and maintain human dignity during and after man-made crises and disasters associated with natural hazards, as well as to prevent and strengthen preparedness for when such situations occur” (Development Initiatives, 2021, p. 83). Therefore, at its core, humanitarian aid should be humble—other-oriented, teachable, and committed to accurate information about what is and is not helping achieve humanitarian response goals. Theoretically, the more humanitarian aid leaders, workers, teams, and organizations are characterized by humility and other virtues, the more they might optimize their effectiveness, functioning, and health (Wang et al., 2021).

Such a possibility is consistent with what Shannonhouse et al. (2019) found in a qualitative study of 13 peer-nominated exemplars of humanitarian aid leader humility. This sample reported anecdotal observational evidence that aid leader humility has many levels of benefits in a humanitarian aid context. First, it benefits *humanitarian aid personnel* (i.e., “all workers engaged by humanitarian agencies, whether internationally or nationally recruited,^1^ or formally or informally retained from the beneficiary community, to conduct the activities of that agency,” OCHA, 2003, p. 15). Specifically, leader humility benefits aid personnel by enhancing their job satisfaction and engagement, improving their individual and team performance, and inspiring their team’s cohesion and growth-mindedness. In addition, it has benefits for humanitarian leaders themselves (by making their work more meaningful and by helping them develop other valued virtues), for aid organizations (by preventing burnout, undermining politics, and fostering better team/worker effectiveness, productivity, and cohesion), and for those receiving humanitarian assistance (by helping them feel more valued, understood, and sacred).

Even so, Shannonhouse et al.’s, 2019 sample of exemplar aid leaders identified several barriers to leader humility in the humanitarian aid context. Barriers noted at the systemic level were that humanitarian aid is ongoing, stressful, and demanding; it involves complex systems and organizations; and it requires leaders to behave in seemingly nonhumble ways (e.g., assertive and opportunistic) to garner interest for strategic marketing and fundraising. Barriers identified at the individual level included leaders feeling overstressed and overburdened, leaders having problematic employees, and leaders exhibiting pride and narcissism (Shannonhouse et al., 2019).

Importantly, this latter barrier might be somewhat unique to humanitarian aid contexts. Studies in other organizational contexts—including a multinational Fortune 500 health insurance organization (Owens et al., 2015), the U.S. military (Swain and Korenman, 2018), and traditional business firms in China (Chen, 2018)—have found evidence of a complex and paradoxical relationship between leader trait humility and narcissism. For example, Owens et al. (2015) and Swain and Korenman (2018) found that leaders perceived as high in both trait humility and *narcissism* (defined as heightened self-focus, assertiveness, confidence, entitlement, and ambition; Ames et al., 2006; Owens et al., 2015) were perceived most positively (e.g., as higher in leadership effectiveness and potential) and had workers who demonstrated the most positive organizational outcomes (e.g., higher job engagement and performance). This phenomenon is called the *humility–narcissism paradox* (Jankowski et al., 2019), whereby leader humility and narcissism are each paradoxically associated with positive follower and leader outcomes. However, in studying religious leaders, Jankowski et al. (2019) found this paradox may not characterize highly religious people; that may include personnel in faith-based aid organizations.

## Conceptual framework explaining the contributions of aid leader humility to outcomes

**Figure 1:**
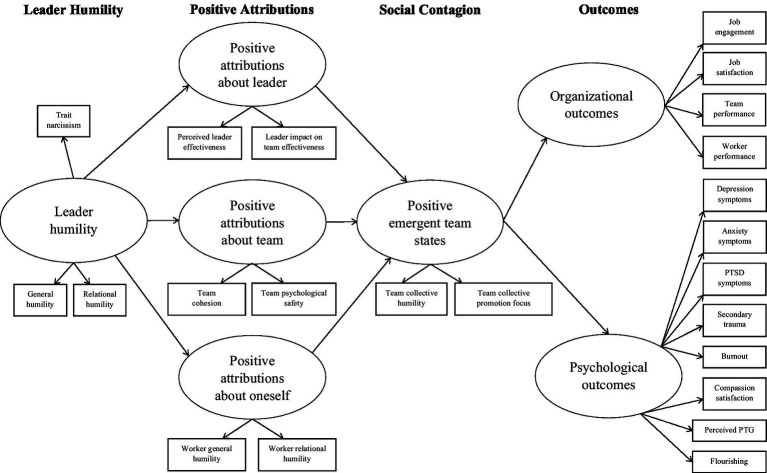
Conceptual model of the contributions of humanitarian-aid leader humility to organizational and psychological outcomes.

This study’s conceptual framework (see Figure 1) integrates the social contagion (Owens and Hekman, 2016) and positive attribution hypotheses of leader humility (Qin et al., 2020). Not only are these hypotheses empirically supported (see Chandler et al., 2023, for reviews), but they also are theoretically consonant because they emphasize the interpersonal nature of humility and its correlates and consequences. In addition, the integration of these hypotheses helps bridge the divide between the two main groups of leader humility scholars—macro-oriented scholars who operationalize leader humility as a stable trait and micro-oriented scholars who operationalize it as a state-like response tendency that emerges based on situational cues (Chandler et al., 2023).

As Figure 1 illustrates, we posit that when workers experience their leader as humble, they are more likely to make positive attributions about their leader (e.g., “my leader is effective and has a strong positive impact on our team”), team (e.g., “my team is cohesive and psychologically safe”), and themselves (e.g., “I also am humble, and humility guides my work”). These positive attributions contribute to emergent positive states of team collective humility and collective promotion focus (growth-mindedness), which spread throughout the team (social contagion) and come to characterize their shared orientation toward their relationships and work (Owens and Hekman, 2016). Ultimately, the leader’s humility contributes directly to some positive organizational and psychological outcomes but mainly contributes indirectly to these outcomes via positive attributions and emergent team states that gradually become defining team traits.

## Overview and hypotheses

The current series of studies addresses several gaps in the literatures on leader humility and humanitarian aid work. First, it addresses the need to extend leader humility research into the humanitarian aid context, where there are such dire and broad-reaching human needs, combined with very difficult and complex working conditions (Wang et al., 2021). Second, it evaluates two key hypotheses of leader humility (the social contagion and positive attribution hypotheses) in a novel organizational and cultural context—Medair, a large, faith-based aid organization that is headquartered in Europe and conducts humanitarian response projects in Africa, the Middle East, and Asia. Third, it expands leader humility research beyond Asia, Europe, and North America, where almost all existing studies have taken place (see Appendix S1 and Chandler et al., 2023). Fourth, it addresses a dearth of humanitarian aid studies that are multilevel and assess a broad array of outcomes. Finally, this study attempts to remedy the common method biases that plague most humanitarian aid research (e.g., most studies have used only one data source and/or data-collection timepoint; *cf.* Podsakoff et al., 2012) and the questionable research practices that may plague leader humility research more broadly (e.g., none of the 65 leader humility articles in Supplementary Table S1 preregistered their study hypotheses/plans, raising concerns about pervasive *p*-hacking, selective reporting, and HARKing [hypothesizing after results are known]; Bosnjak et al., 2022).

**Figure 2:**
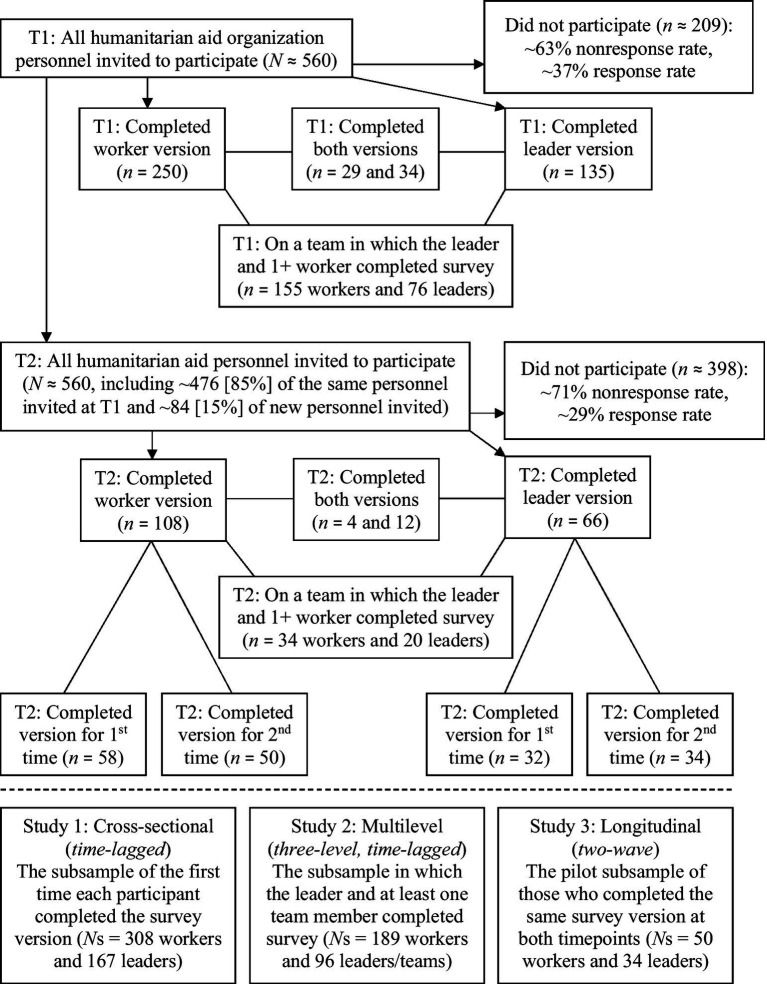
Participant flow chart and adapted project structure.

This project’s hypotheses, plans, and materials were preregistered on the Open Science Framework (OSF).^2^ We originally planned this project to be a two-wave longitudinal study. However, consistent with prior studies that have found it difficult to conduct research in a humanitarian aid context^3^ (e.g., response rates are often as low as 10 to 20%; Ehrenreich and Elliott, 2004; Strohmeier et al., 2018), we had to adapt our project structure. See Figure 2 for a participant flow chart that, among other things, depicts this adaptation process.

Ultimately, we adapted the project into a series of three studies. Study 1 became a cross-sectional, time-lagged study comprised of the subsample of participants whenever those respondents completed a survey version for the first time. Study 2 became a three-level (worker, leader, and team), time-lagged study comprised of the subsample of participants from teams in which the leader and at least one of that leader’s team members completed corresponding survey versions. Finally, Study 3 stayed as the preregistered plan for a two-wave longitudinal study but was adapted into a pilot study, given the small subsample who completed the same survey version at both waves. Even with these adaptations, we kept our same preregistered hypotheses.

*H1*: Cross-sectionally, *workers’ ratings of their leader’s humility* and *team’s collective humility* will be: (a) related to *positive worker-rated organizational outcomes and attributions* (job engagement, job satisfaction, team performance, team collective promotion focus, perceived leader effectiveness, and perceived leader impact on team effectiveness), and *psychological outcomes* (compassion satisfaction, perceived posttraumatic growth, and flourishing); and (b) inversely related to *negative worker psychological outcomes* (e.g., depression and anxiety symptoms, Post-Traumatic Stress Disorder [PTSD] symptoms, secondary traumatic stress, and burnout). As in previous research (Owens et al., 2013; Owens and Hekman, 2016), we expect these relations even adjusting for team cohesion, team psychological safety, and worker sociodemographics (age, gender, time at organization, and time under leader).^4^

*H2*: Cross-sectionally, leaders’ ratings of their own humility and team’s humility will be: (a) positively related to *positive leader-rated organizational outcomes* (leaders’ job engagement, job satisfaction, ratings of their team’s performance, and ratings of their individual workers’ job performance) and *psychological outcomes* (leaders’ compassion satisfaction, perceived posttraumatic growth, and flourishing); and (b) inversely related to *negative leader psychological outcomes* (leaders’ depression symptoms, anxiety symptoms, PTSD symptoms, secondary traumatic stress, and burnout). We expect these relations even controlling for team cohesion, team psychological safety, and leader sociodemographics.

*H3*: Longitudinally, we expect Time 1 leader humility will be related to more positive organizational and psychological outcomes at Time 2. We expect these relations after controlling for team cohesion, team psychological safety, and worker/leader sociodemographics.

*H4*: Drawing on the findings of Owens et al. (2015) and Swain and Korenman (2018), we expect leader humility will paradoxically interact with leader narcissism^5^ in predicting many expected relationships in Hypotheses 1 through 3. That is, we expect high leader humility and narcissism will each be related to better organizational and psychological outcomes.

In our adapted project structure, Studies 1 and 2 test Hypotheses 1, 2, and 4, examining the correlates and contributions of leader humility. Study 3 tests Hypotheses 3 and 4, evaluating its consequences using an outcome-wide longitudinal approach (VanderWeele et al., 2020).

## Study 1: cross-sectional correlates of leader and team humility

### Method

#### Participants and procedure

This time-lagged study occurred in two waves, spaced 6 months apart. Time 1 (T1) data was collected from May to June 2018, and Time 2 (T2) data collection was from November 2018 to January 2019. At T1 and T2, the aid organization (Medair) consisted of around 130 personnel at their headquarters office in Europe and around 430 personnel at their country program offices in Africa, the Middle East, and Asia (~130 internationally recruited and ~ 300 nationally recruited personnel). Based on the length of time that Study 1 respondents reported working at Medair, we estimate 85% of T1 eligible personnel were still at Medair at T2, suggesting collectively about 644 (560 + 84) employees were eligible to participate at one or both study waves. Of this pool, 475 of the organization’s personnel participated in Study 1. See Table 1 for demographic characteristics of this sample, which consisted of 308 workers (*M*_age_ = 34.46, *SD* = 8.82, range = 20–60) and 167 leaders (*M*_age_ = 37.45, *SD* = 9.34, range = 23–60). Because 59 completed both the Leader and Team Member survey versions, the Study 1 response rate was 416/644 (64.60%).

**Table 1:** Demographic characteristics of the study 1 sample.

Characteristic	Leaders	Workers
	*n* (%)	*n* (%)
Gender identity		
Male	98 (58.7)	161 (52.3)
Female	69 (41.3)	147 (47.7)
Nationality		
Afghan	13 (7.8)	32 (10.4)
American	20 (12.0)	19 (6.2)
British	17 (10.2)	19 (6.2)
Canadian	6 (3.6)	5 (1.6)
Congolese	9 (5.4)	30 (9.7)
Dutch	13 (7.8)	15 (4.9)
French	6 (3.6)	11 (3.6)
German	5 (3.0)	5 (1.6)
Iraqi	15 (9.0)	37 (12.0)
Jordanian	3 (1.8)	10 (3.2)
Kenyan	9 (5.4)	7 (2.3)
Lebanese	11 (6.6)	19 (6.2)
Somalian	3 (1.8)	22 (7.1)
South Sudanese	5 (3.0)	27 (8.8)
Swiss	8 (4.8)	18 (5.8)
Other	24 (14.4)	32 (10.4)
Length of time at organization		
Under 6 months	15 (9.0)	46 (14.9)
Between 6 months and 1 year	20 (12.0)	47 (15.3)
Between 1 and 2 years	31 (18.6)	59 (19.2)
Between 2 and 3 years	31 (18.6)	55 (17.9)
Between 3 and 4 years	21 (12.6)	34 (11.0)
Between 4 and 5 years	14 (8.4)	22 (7.1)
Between 5 and 10 years	23 (13.8)	29 (9.4)
Over 10 years	12 (7.2)	16 (5.2)
Length of time in current leader role		
Under 6 months	41 (24.6)	
Between 6 months and 1 year	40 (24.0)	
Between 1 and 2 years	36 (21.6)	
Between 2 and 3 years	29 (17.4)	
Between 3 and 4 years	7 (4.2)	
Between 4 and 5 years	5 (3.0)	
Between 5 and 10 years	7 (4.2)	
Over 10 years	2 (1.2)	
Length of time under current leader		
Under 6 months		109 (35.4)
Between 6 months and 1 year		93 (30.2)
Between 1 and 2 years		61 (19.8)
Between 2 and 3 years		28 (9.1)
Between 3 and 4 years		6 (1.9)
Between 4 and 5 years		5 (1.6)
Between 5 and 10 years		5 (1.6)
Over 10 years		1 (0.3)
Personnel category in organization		
Nationally recruited personnel	68 (40.7)	182 (59.1)
Internationally recruited personnel	72 (43.1)	47 (15.3)
Headquarters personnel	27 (16.2)	79 (25.6)
Organizational group		
Country programs	124 (74.3)	205 (66.6)
*Africa region*	52 (31.1)	97 (31.5)
*Asia region*	44 (26.3)	65 (21.1)
*Middle East region*	26 (15.6)	43 (14.0)
*Other*	2 (1.2)	0 (0.0)
Affiliate offices	19 (11.4)	34 (11.0)
Headquarters	24 (14.4)	69 (22.4)

Leader subsample *n* = 167; worker subsample *n* = 308.

During the research design phase, the aid organization requested all 21 study measures be available not only in English but also in French and Arabic, the two other languages spoken by a sizable proportion of their employees. Hence, all measures that did not yet have Arabic- (*k* = 21) or French-language versions (*k* = 17) were translated and back-translated by a professional translation company and then meticulously field checked and refined by a few native-speaking leaders in the aid organization. This rigorous translation and back-translation process adhered to Brislin’s (1970) procedures for optimizing the cross-cultural validity and construct equivalence of all measures and was consistent with prior leader-humility studies (Hu et al., 2018; Owens et al., 2019). All materials were then preregistered and posted at https://osf.io/gtu9h/files/.

After approval by the Wheaton College Institutional Review Board (1123479–1), all then-current employees of the organization were invited to participate. Inclusion criteria required participants to be (a) 18 years or older; (b) able to access the internet; (c) able to read English, French, or Arabic at or above a 6^th^-grade reading level (each survey’s reading level); and (d) able to identify a direct supervisor they considered a leader (to be eligible for the Team Member survey version) or indicate they directly supervise at least two workers (to be eligible for the Leader survey version). Participants self-identified if they were a team member and/or leader. They then completed the online administered Team Member or Leader survey. The median time it took to complete the Team Member and Leader versions were 80.83 and 61.88 min, respectively. For each version, respondents could select to take the survey in English, French, or Arabic. The same versions were administered at both waves. At the request of Medair, there was no monetary incentive for participating (to encourage intrinsically motivated participation). Of note, our consent and debriefing forms offered referral resources to participants who experienced negative reactions to the survey or who reported significant mental health difficulties.

#### Measures

All measures were initially developed in English and validated with English-speaking samples, so we intended to conduct preliminary validation and measurement invariance analyses based on survey language. However, because so few respondents chose to complete the surveys in Arabic (Team Member: *n* = 48, 15.6%; Leader: *n* = 14, 8.4%) or French (Team Member: *n* = 51, 16.6%; Leader: *n* = 19, 11.4%), such analyses were deemed methodologically inappropriate. Even so, because such a low portion of respondents used these language versions, all participants were included in the Study 1 through 3 samples, regardless of the language version they chose.

All measures yielded scores reflecting higher levels of the assessed construct. Mean-item scores were calculated for most scales, except for five measures that yielded summed scores—the below-described measures of trait narcissism, depression symptoms, anxiety symptoms, PTSD symptoms, and professional quality of life. Unless otherwise noted, each measure was included in both survey versions. Tables 2, 3 report reliability analyses and possible range of scores for each scale.

**Table 2:** Study 1 bivariate and partial correlations between humility variables and organizational outcomes among aid workers.

Variable	1	2	3	4	5	6	7	8	9	10	11
1. Leader general humility (worker-rated)	(0.93)	0.72^***^	0.26^***^	0.27^***^	0.42^***^	0.63^***^	0.70^***^	0.31^***^	0.37^***^	0.31^***^	0.51^***^
2. Leader relational humility (worker-rated)	0.69^***^	(0.90)	0.16	0.37^***^	0.29^***^	0.54^***^	0.57^***^	0.23^***^	0.21^***^	0.20^***^	0.41^***^
3. Worker general humility	0.12	0.09	(0.80)	0.31^***^	0.44^***^	0.30^***^	0.24^***^	0.32^***^	0.34^***^	0.39^***^	0.31^***^
4. Worker relational humility	0.18^***^	0.33^***^	0.26^***^	(0.75)	0.20^***^	0.23^***^	0.26^***^	0.20^***^	0.21^***^	0.22^***^	0.20^***^
5. Team collective humility	0.14	0.09	0.28^***^	0.05	(0.91)	0.38^***^	0.35^***^	0.43^***^	0.55^***^	0.32^***^	0.51^***^
6. Leader effectiveness	0.54^***^	0.51^***^	0.15	0.14	0.09	(0.94)	0.77^***^	0.46^***^	0.44^***^	0.37^***^	0.43^***^
7. Leader impact on team effectiveness	0.64^***^	0.57^***^	0.07	0.17^***^	0.02	0.69^***^	(0.95)	0.41^***^	0.44^***^	0.37^***^	0.45^***^
8. Team performance	0.12	0.13	0.18^***^	0.10	0.16	0.29^***^	0.19^***^	(0.91)	0.58^***^	0.39^***^	0.44^***^
9. Team collective promotion focus	0.10	0.05	0.14	0.08	0.18^***^	0.20^***^	0.17	0.39^***^	(0.92)	0.43^***^	0.52^***^
10. Worker job engagement	0.16	0.13	0.27^***^	0.14	0.06	0.19^***^	0.17	0.22^***^	0.21^***^	(0.85)	0.55^***^
11. Worker job satisfaction	0.37^***^	0.31^***^	0.18^***^	0.11	0.26^***^	0.27^***^	0.29^***^	0.28^***^	0.32^***^	0.46^***^	(0.91)
Team cohesion	0.46^***^	0.28^***^	0.36^***^	0.23^***^	0.69^***^	0.46^***^	0.50^***^	0.50^***^	0.65^***^	0.43^***^	0.48^***^
Team psychological safety	0.34^***^	0.40^***^	0.14	0.14	0.45^***^	0.24^***^	0.22^***^	0.21^***^	0.30^***^	0.13	0.33^***^
Worker age	0.11	0.08	0.05	0.04	0.05	−0.03	−0.03	−0.08	0.04	0.03	0.01
Worker gender (1 = female)	−0.08	0.00	−0.17^***^	−0.05	−0.16	−0.18^***^	−0.17	−0.10	−0.21^***^	−0.22^***^	−0.12
Worker length of time at aid organization	−0.05	0.01	−0.04	−0.04	−0.01	−0.15	−0.18^***^	−0.14	−0.04	−0.13	−0.01
Worker length of time under current leader	−0.05	−0.03	−0.01	−0.02	0.00	−0.06	−0.01	−0.05	0.02	0.04	0.05
Possible range	1–5	1–5	1–5	1–5	1–5	1–5	1–7	1–5	1–5	1–6	1–7
*M*	3.99	3.81	4.51	4.05	4.12	3.92	5.28	3.69	3.98	4.67	5.09
*SD*	0.92	0.72	0.45	0.46	0.76	0.85	1.60	0.73	0.93	0.96	0.96
Number of items	9	16	9	16	9	11	3	4	4	9	16

*N* = 308. Bivariate correlations are presented above the diagonal, and partial correlations are presented below the diagonal. All bivariate and partial correlation coefficients that are significant at ***p ≤ 0.001 are presented in boldface and italicized type. Cronbach’s alphas are presented in parentheses along the diagonal.

**Table 3:** Study 1 bivariate and partial correlations between humility variables and psychological outcomes among aid workers.

Variable	1	2	3	4	5	6	7	8	9	10	11	12	13
1. Leader general humility	(0.93)	0.72^***^	0.26^***^	0.27^***^	0.42^***^	−0.18^***^	−0.14	−0.18^***^	0.09	−0.20^***^	0.20^***^	0.06	0.14
2. Leader relational humility	0.69^***^	(0.90)	0.16	0.37^***^	0.29^***^	−0.13	−0.13	−0.23^***^	−0.06	−0.17^***^	0.04	−0.06	0.05
3. Worker general humility	0.12	0.09	(0.80)	0.31^***^	0.44^***^	−0.14	−0.09	0.03	0.15	−0.29^***^	0.45^***^	0.26^***^	0.28^***^
4. Worker relational humility	0.18^***^	0.33^***^	0.26^***^	(0.75)	0.20^***^	−0.23^***^	−0.18^***^	−0.21^***^	−0.01	−0.36^***^	0.24^***^	0.18^***^	0.25^***^
5. Team collective humility	0.14	0.09	0.28^***^	0.05	(0.91)	−0.17	−0.12	−0.02	0.21^***^	−0.21^***^	0.30^***^	0.16	0.28^***^
6. Depression symptoms	−0.07	−0.06	−0.06	−0.18^***^	−0.01	(0.85)	0.71^***^	0.51^***^	0.10	0.38^***^	−0.20^***^	−0.13	−0.35^***^
7. Anxiety symptoms	−0.04	−0.05	−0.03	−0.14	0.04	0.70^***^	(0.89)	0.41^***^	0.10	0.30^***^	−0.13	−0.09	−0.30^***^
8. PTSD symptoms	−0.14	−0.17^***^	0.05	−0.20^***^	0.09	0.51^***^	0.40^***^	(0.87)	0.36^***^	0.30^***^	−0.03	0.01	−0.21^***^
9. Secondary traumatic stress	0.03	−0.05	0.06	−0.06	0.15	0.15	0.13	0.35^***^	(0.81)	0.24^***^	0.34^***^	0.36^***^	0.14
10. Burnout	−0.08	−0.07	−0.24^***^	−0.32^***^	−0.05	0.36^***^	0.26^***^	0.27^***^	0.27^***^	(0.59)	−0.47^***^	−0.15	−0.34^***^
11. Compassion satisfaction	0.03	−0.05	0.35^***^	0.17^***^	0.04	−0.13	−0.07	−0.03	0.25^***^	−0.46^***^	(0.87)	0.48^***^	0.47^***^
12. Perceived posttraumatic growth	−0.04	−0.09	0.18^***^	0.14	0.02	−0.09	−0.07	−0.02	0.28^***^	−0.14	0.41^***^	(0.93)	0.24^***^
13. Psychological flourishing	0.00	−0.04	0.18^***^	0.19^***^	0.09	−0.31^***^	−0.26^***^	−0.21^***^	0.07	−0.31^***^	0.39^***^	0.17^***^	(0.93)
Team cohesion	0.46^***^	0.28^***^	0.36^***^	0.23^***^	0.69^***^	−0.22^***^	−0.18^***^	−0.09	0.18^***^	−0.24^***^	0.41^***^	0.24^***^	0.31^***^
Team psychological safety	0.34^***^	0.40^***^	0.14	0.14	0.45^***^	−0.13	−0.18^***^	−0.20^***^	−0.13	−0.26^***^	0.11	−0.05	0.14
Worker age	0.11	0.08	0.05	0.04	0.05	−0.14	−0.14	−0.08	−0.05	−0.03	−0.04	−0.01	0.01
Worker gender (1 = female)	−0.08	0.00	−0.17^***^	−0.05	−0.16	0.07	0.06	−0.09	−0.25^***^	−0.01	−0.21^***^	−0.14	−0.15
Worker length of time at organization	−0.05	0.01	−0.04	−0.04	−0.01	−0.05	−0.04	−0.05	−0.11	0.00	−0.05	−0.07	0.00
Worker length of time under leader	−0.05	−0.03	−0.01	−0.02	0.00	−0.03	−0.01	−0.05	−0.13	−0.04	−0.01	−0.06	−0.01
Possible range	1–5	1–5	1–5	1–5	1–5	0–28	0–21	0–32	10–50	10–50	10–50	0–5	1–7
*M*	3.99	3.81	4.51	4.05	4.12	4.37	2.95	7.75	20.05	20.34	40.92	3.26	5.86
*SD*	0.92	0.72	0.45	0.46	0.76	4.60	3.83	6.51	6.67	4.92	6.40	1.19	0.99
Number of items	9	16	9	16	9	9	7	8	10	10	10	10	8

*N* = 308. PTSD, post-traumatic stress disorder. Bivariate correlations are presented above the diagonal, and partial correlations are presented below the diagonal. All bivariate and partial correlation coefficients that are significant at ***p ≤ 0.001 are presented in boldface and italicized type. Cronbach’s alphas are presented in parentheses along the diagonal.

##### Humility-related variables

*General humility* was measured with the Expressed Humility Scale (9 items; e.g., “This leader [I] actively seek[s] feedback, even if it is critical”; Owens et al., 2013), and *relational humility* was measured with the Relational Humility Scale (16 items; “Most people would consider him/her [me] a humble person”; Davis et al., 2011). Both scales have a 5-point Likert scale (1 = *strongly disagree* to 5 = *strongly agree*). We used two versions of each scale—an informant-report version (to measure workers’ perceptions of their leader’s general and relational humility) and self-report version (to measure workers’ and leaders’ perceptions of their own general and relational humility). On only the Leader version, leaders rated their own *trait narcissism*, using the Narcissistic Personality Inventory–16 (Ames et al., 2006), which has 16 item pairs and uses dichotomous scaling that yields a sum of keyed responses (e.g., “I am an extraordinary person” [1 point] vs. “I am much like everybody else”).

On both survey versions, *team humility* was assessed via the 9-item Collective Humility Scale (e.g., “Members of this team are willing to learn from one another”; Owens and Hekman, 2016). This measure uses a 5-point Likert scale (1 = *strongly disagree* to 5 = *strongly agree*).

##### Positive leader and team attributions

On only the Team Member survey, workers rated their *leader’s perceived effectiveness* using the Leader Performance Scale (11 items, e.g., “Management and administration: forms goals, allocates resources to meet them, and monitors progress toward them,” Colbert et al., 2008) and *perceived team impact* using the Perceived Leader Impact on Team Effectiveness Scale (3 items, e.g., “The way he/she acts is crucial to the team’s effectiveness,” Rego et al., 2018). These use a 5-point (1 = *somewhat below requirements* to 5 = *consistently exceeds requirements*) and 7-point scale (1 = *The statement does not apply to this leader at all* to 7 = *The statement applies completely to this leader*), respectively.

On both survey versions, workers and leaders rated their positive attributions about their *team’s cohesion*, *psychological safety*, and *collective promotion focus* (i.e., growth-mindedness). Both the Team Cohesion Scale (6 items; e.g., “The members of my team are cooperative with each other”; Podsakoff and MacKenzie, 1994) and Team Psychological Safety Scale (7 items; e.g., “It is safe to take a risk on this team”; Edmondson, 1999) use a 7-point Likert scale (1 = *strongly disagree* to 7 = *strongly agree*). The Team Collective Promotion Focus Scale (4 items; e.g., “In general, our team is focused on achieving our hopes and aspirations”; Owens and Hekman, 2016) uses a 5-point Likert scale (1 = *not at all true of our team* to 5 = *very true of our team*).

##### Organizational outcomes

The Utrecht Work Engagement Scale–9 (9 items; e.g., “My job inspires me”; Schaufeli et al., 2006) assessed *job engagement* on a 7-point scale (0 = *never* to 6 = *every day*). The Job Satisfaction Scale (16 items; e.g., “Now, taking everything into consideration, how do you feel about your job as a whole?”; Warr et al., 1979) assessed *job satisfaction* on a 7-point scale (1 = *I’m extremely dissatisfied* to 7 = *I’m extremely satisfied*). The Team Performance Scale (4 items; e.g., “How would you judge the overall quality of the work performed by the team?”; Walumbwa et al., 2008) measured workers’ and leaders’ perceptions of their *team’s performance*, using a 5-point Likert scale (1 = *consistently performs way below expectations* to 5 = *consistently performs way beyond expectations*).

On only the Leader survey version, leaders completed the Supervisor-Rated Task Performance Scale (4 items; e.g., “How would you judge the overall quality of this individual’s work?”; Walumbwa et al., 2008) to assess leader perceptions of *individual workers’ job performance*. Leaders (*N* = 167) rated the performance of up to five team members, using a 5-point scale (1 = *consistently performs way below expectations* to 5 = *consistently performs way beyond expectations*). Every leader’s team had at least two rated members; many teams included three (*n* = 153; 91.62%), four (*n* = 125, 74.85%), or five rated workers (*n* = 102; 61.08%).

##### Psychological outcomes

The Patient Health Questionnaire–9 (9 items; e.g., “Feeling down, depressed, or hopeless; Kroenke et al., 2001) measured *depression symptoms*, and the Generalized Anxiety Disorder–7 (7 items; e.g., “Feeling nervous, anxious, or on edge”; Spitzer et al., 2006) measured *anxiety symptoms*. Both scales ask about the past 2 weeks and use a 4-point scale (0 = *not at all* to 3 = *nearly every day*). Past-month *PTSD symptoms* were assessed via the 8-item PTSD CheckList for DSM-5 (e.g., “Repeated, disturbing, and unwanted memories of the stressful experience; Price et al., 2016), using a 5-point scale (0 = *not at all* to 4 = *extremely*).

The Professional Quality of Life Scale, Version 5 (Stamm, 2010) has a 5-point Likert scale (1 = *never* to 5 = very often) and was used to assess past-month *secondary traumatic stress* (10 items; e.g., “I cannot recall important parts of my work with trauma victims”), *burnout* (10 items; e.g., “I feel overwhelmed because my work load seems endless”), and *compassion satisfaction* (10 items; e.g., “I get satisfaction from being able to help people”). The 10-item Posttraumatic Growth Inventory–Short Form (e.g., “I discovered that I’m stronger than I thought I was”; Cann et al., 2010) assessed *perceived posttraumatic growth*, using a 6-point scale (0 = *I did not experience this change as a result of my humanitarian aid work* to 5 = *I experienced this change to a very great degree as a result of my humanitarian aid work*). Finally, *flourishing* was assessed via the 8-item Flourishing Scale (e.g., “I lead a purposeful and meaningful life”; Diener et al., 2010), which uses a 7-point scale (1 = *strongly disagree* to 7 = *strongly agree*).

### Results

In Study 1, we tested Hypotheses 1 and 2 using partial correlation analyses. We also conducted follow-up exploratory hierarchical regression analyses and frequency analyses. All analyses focused solely on the individual level of analysis in that we focused on workers’ ratings of all variables to test Hypothesis 1 and leaders’ ratings of all variables to test Hypothesis 2.

*H1*: Correlates of Worker-Rated Leader Humility and Team Collective Humility.

Results of bivariate and partial correlation analyses are presented in Tables 2, 3. Hypothesis 1 was partially supported. After controlling for worker sociodemographics and worker-rated team cohesion and psychological safety, worker-rated leader humility was related to a few positive organizational and psychological outcomes. Specifically, worker-rated leader general and relational humility were associated with higher perceived leader effectiveness, perceived leader impact on team effectiveness, and worker job satisfaction. Worker-rated leader relational humility was also associated with lower PTSD symptoms. After controlling for the same covariates, worker-rated team collective humility was only related to higher team collective promotion focus and worker job satisfaction. It was unrelated to any psychological outcomes.

To explore these findings further, we ran several hierarchical regression models. These results are presented in Supplementary Tables S3, S4. Consistent with our conceptual framework (Figure 1), worker-rated leader and team humility contributed uniquely to workers’ positive attributions about their leaders and team. Workers’ positive attributions about their leader (perceived leader effectiveness and impact on team effectiveness) were predicted by worker-rated leader general and relational humility. Their positive attributions about their team’s cohesion were predicted by worker-rated leader general humility, and their positive attributions about their team’s psychological safety were predicted by worker-rated leader relational humility. Team collective humility predicted both positive team attributions (of team cohesion and psychological safety).

Perceived team cohesion was the most consistent contributor to workers’ organizational and psychological outcomes, and once accounting for its influence, the only outcome still predicted by worker-rated leader general humility and team collective humility was workers’ job satisfaction. Even so, workers’ ratings of their own (i.e., self-rated) humility contributed uniquely to workers’ (a) higher job engagement (predicted by general humility), (b) lower depression and PTSD symptoms (by relational humility), (c) lower burnout (by both), and (d) higher compassion satisfaction (by general humility) and flourishing (by relational humility).

*H2*: Correlates of Leader-Rated Personal Humility and Team Collective Humility.

See Tables 4, 5 for results of bivariate and partial correlation analyses of leaders’ responses. Hypothesis 2 was partially supported. After controlling for leader sociodemographics and leader-rated team cohesion and psychological safety, leaders’ self-rated general humility was related to (a) higher team collective promotion focus, (b) higher leader job engagement and job satisfaction, (c) lower burnout, and (d) higher compassion satisfaction. Unexpectedly, it was also related to higher secondary traumatic stress.^6^ Leaders’ self-rated relational humility was unrelated to any organizational or psychological outcomes, but leader ratings of their team’s collective humility were related to higher team collective promotion focus and higher leader job engagement, job satisfaction, compassion satisfaction, and perceived posttraumatic growth.

**Table 4:** Study 1 bivariate and partial correlations between humility variables and organizational outcomes among aid leaders.

Variable	1	2	3	4	5	6	7	8
1. Leader general humility (self-rated)	(0.82)	0.33^***^	0.31^***^	0.19	0.21	0.38^***^	0.39^***^	0.31^***^
2. Leader relational humility (self-rated)	0.28^***^	(0.70)	0.10	0.09	0.08	0.11	0.10	0.05
3. Team collective humility	0.18	0.01	(0.89)	0.35^***^	0.34^***^	0.51^***^	0.36^***^	0.42^***^
4. Individual workers’ performance (mean)	0.07	−0.01	0.18	(0.92)	0.74^***^	0.21	0.19	0.28^***^
5. Team performance	0.12	0.01	0.20	0.71^***^	(0.85)	0.22	0.15	0.23
6. Team collective promotion focus	0.30^***^	0.05	0.31^***^	0.03	0.07	(0.88)	0.42^***^	0.35^***^
7. Leader job engagement	0.36^***^	0.08	0.26^***^	0.10	0.08	*0.35* ^***^	(0.88)	0.57^***^
8. Leader job satisfaction	0.25^***^	0.02	0.26^***^	0.17	0.14	0.21	0.52^***^	(0.92)
Team cohesion	0.26^***^	0.19	0.55^***^	0.39^***^	0.33^***^	0.46^***^	0.25^***^	0.32^***^
Team psychological safety	0.28^***^	0.20	0.33^***^	0.25^***^	0.17	0.22	0.14	0.21
Leader age	0.10	0.04	0.10	0.11	0.04	0.07	−0.03	−0.05
Leader gender (1 = female)	−0.03	0.13	−0.18	−0.04	−0.01	−0.20	−0.14	−0.22
Leader length of time at aid organization	−0.01	−0.10	0.15	−0.02	0.03	0.07	0.02	0.05
Leader length of time in current leader role	0.08	0.01	0.17	0.03	0.10	0.15	0.01	−0.04
Possible range	1–5	1–5	1–5	1–5	1–5	1–5	1–6	1–7
*M*	4.48	3.91	3.93	3.51	3.52	4.02	4.59	5.07
*SD*	0.45	0.42	0.70	0.54	0.58	0.73	0.92	0.96
Number of items	9	16	9	20	4	4	9	16

*N* = 167. Bivariate correlations are presented above the diagonal, and partial correlations are presented below the diagonal. All bivariate and partial correlation coefficients that are significant at ***p ≤ 0.001 are presented in boldface and italicized type. Cronbach’s alphas are presented in parentheses along the diagonal.

**Table 5:** Study 1 bivariate and partial correlations between humility variables and psychological outcomes among aid leaders.

Variable	1	2	3	4	5	6	7	8	9	10	11
1. Leader general humility (self-report)	(0.82)	0.33^***^	0.31^***^	−0.14	−0.06	−0.13	0.22	−0.33^***^	0.46^***^	0.17	0.25^***^
2. Leader relational humility (self-report)	0.28^***^	(0.70)	0.10	−0.16	−0.10	−0.17	−0.02	−0.16	0.16	0.00	0.08
3. Team collective humility	0.18	0.01	(0.89)	−0.18	−0.15	−0.17	0.06	−0.30^***^	0.41^***^	0.13	0.21
4. Depression symptoms	−0.07	−0.15	−0.03	(0.88)	0.78^***^	0.64^***^	0.34^***^	0.60^***^	−0.30^***^	−0.10	−0.39^***^
5. Anxiety symptoms	0.00	−0.09	−0.04	0.76^***^	(0.84)	0.61^***^	0.43^***^	0.55^***^	−0.21	0.03	−0.30^***^
6. PTSD symptoms	−0.04	−0.12	−0.07	0.63^***^	0.59^***^	(0.86)	0.49^***^	0.46^***^	−0.20	−0.01	−0.35^***^
7. Secondary traumatic stress	0.31^***^	0.01	0.10	0.32^***^	0.42^***^	0.43^***^	(0.82)	0.18	0.27^***^	0.28^***^	−0.01
8. Burnout	−0.26^***^	−0.13	−0.10	0.54^***^	0.52^***^	0.43^***^	0.18	(0.69)	−0.64^***^	−0.25^***^	−0.53^***^
9. Compassion satisfaction	0.40^***^	0.12	0.24^***^	−0.24^***^	−0.16	−0.17	0.28^***^	−0.59^***^	(0.89)	0.43^***^	0.49^***^
10. Perceived posttraumatic growth	0.18	0.02	0.07^***^	−0.12^***^	0.01	−0.04	0.26^***^	−0.25^***^	0.43^***^	(0.92)	0.19
11. Psychological flourishing	0.18	0.04	0.04	−0.35^***^	−0.28^***^	−0.31^***^	0.04	−0.48^***^	0.45^***^	0.15	(0.93)
Team cohesion	0.26^***^	0.19	0.55^***^	−0.23	−0.20	−0.18	0.02	−0.34^***^	0.39^***^	0.11	0.26^***^
Team psychological safety	0.28^***^	0.20	0.33^***^	−0.17	−0.10	−0.27^***^	−0.27^***^	−0.20	0.16	−0.04	0.21
Worker age	0.10	0.04	0.10	−0.15	−0.10	−0.17	−0.25^***^	−0.13	0.02	0.05	0.14
Worker gender (1 = female)	−0.03	0.13	−0.18	0.21	0.14	−0.02	−0.10	0.21	−0.18	−0.02	−0.03
Worker length of time at organization	−0.01	−0.10	0.15	0.01	0.07	0.01	−0.05	−0.04	0.01	0.21	0.16
Worker length of time under leader	0.08	0.01	0.17	0.05	0.17	0.09	0.14	−0.07	0.13	0.19	0.11
Possible range	1–5	1–5	1–5	0–28	0–21	0–32	10–50	10–50	10–50	0–5	1–7
*M*	4.48	3.91	3.93	4.14	2.85	6.54	19.71	21.19	40.22	3.14	5.80
*SD*	0.45	0.42	0.70	4.35	3.10	5.78	6.40	5.03	6.30	1.18	0.96
Number of items	9	16	9	9	7	8	10	10	10	10	8

*N* = 167. PTSD, post-traumatic stress disorder. Bivariate correlations are presented above the diagonal, and partial correlations are presented below the diagonal. All bivariate and partial correlation coefficients that are significant at ***p ≤ 0.001 are presented in boldface and italicized type. Cronbach’s alphas are presented in parentheses along the diagonal.

Once more, we conducted exploratory hierarchical regression analyses (see Supplementary Tables S5, S6). Of the humility variables, leaders’ perceptions of their team’s collective humility contributed significantly to leaders’ attributions about their team’s cohesion and psychological safety, but leaders’ perceptions of their own personal humility did not. After controlling for sociodemographics, team cohesion, and psychological safety, leaders’ self-rated general humility contributed to (a) higher perceptions of their team’s collective promotion focus, (b) higher ratings of their own job engagement and job satisfaction, (c) lower levels of leader burnout, and (d) higher levels of leader compassion satisfaction and secondary traumatic stress. In addition, leaders’ perceptions of their team’s collective humility contributed to higher leader job satisfaction and perceptions of their team’s collective promotion focus (growth-mindedness).

#### Exploratory analyses of negative mental health outcomes

It is noteworthy that the negative psychological outcomes of depression, anxiety, PTSD, and burnout were moderately to strongly interrelated, both among workers (*r*s = 0.30–0.71) and leaders (*r*s = 0.46–0.78; all *p*s < 0.001). Previous research has found high prevalence rates of these negative mental health outcomes in samples of aid personnel serving in Africa (Ager et al., 2012; Strohmeier et al., 2018), Asia (Lopes Cardozo et al., 2013), the Middle East (Eriksson et al., 2013), and across all three of those regions (Lopes Cardozo et al., 2012). Like those prior studies have done, we calculated the prevalence rates of likely Major Depressive Disorder, Generalized Anxiety Disorder, PTSD, and burnout, based on normed and validated cutoff scores. Using the established cutoffs from Manea et al. (2012), Spitzer et al. (2006), Price et al. (2016), and Stamm (2010), 0.0% (*n* = 0) of Study 1 participants reported clinically significant burnout (or secondary trauma), and the prevalence rates were 10.1% (*n* = 48) for likely Major Depressive Disorder, 6.3% (*n* = 30) for likely Generalized Anxiety Disorder, and 6.5% (*n* = 31) for likely PTSD.^7^

### Discussion

The positive attribution (Qin et al., 2020) and social contagion (Owens and Hekman, 2016) hypotheses of leader humility were each supported quite strongly in Study 1. Workers’ perception of their leader’s humility was associated with positive attributions they had about their leader (as being effective and impactful on their team), team (as being humble, cohesive, and psychologically safe), and themselves (as being humble personally). Workers’ perception of their team’s collective humility was related to positive attributions about their team’s cohesion and psychological safety. These leader-, team-, and self-attributions were linked to many positive organizational and psychological outcomes. Attributions of team cohesion were especially predictive of these outcomes, which included lower worker depression symptoms; higher team humility and performance; and higher worker job engagement, job satisfaction, compassion satisfaction, and psychological flourishing. And yet, even above the influence of team cohesion and sociodemographics, leader and team humility predicted workers’ job satisfaction, and workers’ self-attributions of humility predicted higher job engagement, compassion satisfaction, and flourishing, as well as lower burnout, depression, and PTSD symptoms.

The positive attribution and social contagion hypotheses were supported from leaders’ perspective as well. Leaders’ self-rated humility was related to positive attributions about their team (as being humble, cohesive, psychologically safe, and growth-minded), and these positive team and self-attributions were linked to several positive outcomes. Team cohesion was again a robust contributor to positive organizational and psychological outcomes, yet even controlling for it and sociodemographics, leaders’ self-attributions of humility predicted lower burnout and higher job engagement, job satisfaction, and compassion satisfaction. Leaders’ perceptions of their team’s humility also contributed to leaders’ higher job satisfaction and more positive attributions about their team (as being cohesive, psychologically safe, and growth-minded).

In sum, when workers see their leader as humble, they tend to view their leader as effective and impactful; their team as humble, cohesive, psychologically safe, and growth-minded; and themselves as humble. When leaders view themselves as humble, they tend to view their team as humble, cohesive, psychologically safe, and growth-minded. These positive multi-level attributions are interrelated and are linked to positive outcomes for aid workers and leaders.

Nevertheless, it is worth noting that, for leaders, humility may paradoxically be both a resilience factor and risk factor. Leaders’ self-rated humility was related to several positive outcomes, but it was also related to higher secondary trauma. Figley and Ludick (2017) describe secondary trauma and compassion fatigue as tightly intertwined, calling them the “cost of caring” (p. 574). It may be that leader humility places aid leaders at risk for secondary trauma and compassion fatigue because humble leaders are so other-oriented and compassionate they take on the traumatic stress of the people and populations they serve. Alternatively, consistent with Figley and Ludick’s (2017) Compassion Fatigue Resilience Model, it may be that aid leaders’ humility is indeed linked to higher secondary trauma (because humble aid leaders are likely high in empathy as well), but when humble aid leaders also have a high degree of social support and compassion satisfaction, then they might be resilient against compassion fatigue.

This latter possibility is consistent with the Study 1 finding that, although leaders’ self-rated humility was associated with higher secondary trauma, their team-attributed humility was related to their attributions of team cohesion and safety, and both their self- and team-attributed humility were linked to positive attributions of team growth-mindedness (collective promotion focus). Moreover, leaders’ self-attributed humility was linked with lower burnout and higher job engagement, job satisfaction, and compassion satisfaction. This constellation of reciprocal, multilevel positive attributions may be an attributional style that characterizes humble aid leaders who exhibit compassion fatigue resilience. It probably is particularly resilience-promoting in faith-based aid organizations such as Medair, whose organizational culture places a high value on team cohesion and on guiding virtues like hope, joy, compassion, and integrity (Medair, n.d.).

The uniqueness of Medair’s organizational culture might also help explain this sample’s lower-than-typical prevalence rates of clinically elevated depression (10.1%), anxiety (6.3%), PTSD (6.5%), and burnout (0.0%). In prior humanitarian aid research, prevalence rates have been notably higher for likely depression [usually ranging from 20% (Lopes Cardozo et al., 2012) to 68% (Ager et al., 2012)], anxiety [38% (Strohmeier et al., 2018) to 53% (Ager et al., 2012; Lopes Cardozo et al., 2013)], PTSD [19% (Eriksson et al., 2013; Lopes Cardozo et al., 2013) to 26% (Ager et al., 2012)], and burnout [5% (Ager et al., 2012) to 19% (Strohmeier et al., 2018)]. Yet, previous studies with aid personnel have found that high team cohesion and social support buffer against these mental health problems (Eriksson et al., 2009, 2013; Ager et al., 2012; Lopes Cardozo et al., 2012, 2013). Therefore, Medair’s strong emphasis on team cohesion and supportiveness may help explain the low prevalence rates in this sample. Regardless, like in prior research, this sample demonstrated high interrelatedness among depression, anxiety, PTSD, and burnout symptoms. That finding underscores a vital need for humanitarian organizations to offer private, confidential, and nonstigmatizing support to personnel who report or exhibit significant signs of mental health difficulty.

## Study 2: the multilevel contributions of leader and team humility

In Study 1, we examined the correlates of leader and team humility while focusing on the individual level of analysis (workers’ perspectives first and leaders’ perspectives next). Study 2 builds on these findings by examining the multilevel contributions leader and team humility might have on concurrent worker, team, and leader attributions and outcomes. In Study 2, we move from focusing solely on the individual level of analysis to focusing additionally on the team level of analysis by grouping team members’ ratings into team-level aggregated variables.

### Method

#### Participants and procedure

The Study 2 subsample included 189 humanitarian aid workers, nested in 96 teams, each led by a different supervisor. Hence, 285 personnel participated in Study 2, reflecting a 44.25% response rate (285/644). Teams on average had 2.62 members (*SD* = 1.33; range = 1–6), not including the leader. Sociodemographic characteristics of the workers (*M*_age_ = 34.46, *SD* = 8.82, range = 20–60) and leaders (*M*_age_ = 37.45, *SD* = 9.34, range = 23–60) are presented in Table 6.

**Table 6:** Demographic characteristics of the study 2 sample.

Characteristic	Leaders	Workers
	*n* (%)	*n* (%)
Gender identity		
Male	54 (56.3)	104 (55.0)
Female	42 (43.8)	85 (45.0)
Nationality		
Afghan	7 (7.3)	28 (14.8)
American	13 (13.5)	9 (4.8)
British	11 (11.5)	9 (4.8)
Canadian	3 (3.1)	1 (0.5)
Congolese	7 (7.3)	25 (13.2)
Dutch	10 (10.4)	10 (5.3)
French	5 (5.2)	8 (4.2)
German	3 (3.1)	3 (1.6)
Iraqi	6 (6.3)	19 (10.1)
Jordanian	0 (0.0)	2 (1.1)
Kenyan	6 (6.3)	5 (2.6)
Lebanese	2 (2.1)	13 (6.9)
Somalian	3 (3.1)	19 (10.1)
South Sudanese	1 (1.0)	7 (3.7)
Swiss	6 (6.3)	12 (6.3)
Other	13 (13.5)	19 (10.1)
Length of time at organization		
Under 6 months	4 (4.2)	20 (10.6)
Between 6 months and 1 year	9 (9.4)	28 (14.8)
Between 1 and 2 years	13 (13.5)	40 (21.2)
Between 2 and 3 years	18 (18.8)	30 (15.9)
Between 3 and 4 years	17 (17.7)	26 (13.8)
Between 4 and 5 years	9 (9.4)	17 (9.0)
Between 5 and 10 years	17 (17.7)	18 (9.5)
Over 10 years	9 (9.4)	10 (5.3)
Length of time in current leader role		
Under 6 months	21 (21.9)	
Between 6 months and 1 year	21 (21.9)	
Between 1 and 2 years	22 (22.9)	
Between 2 and 3 years	16 (16.7)	
Between 3 and 4 years	5 (5.2)	
Between 4 and 5 years	4 (4.2)	
Between 5 and 10 years	7 (7.3)	
Over 10 years	0 (0.0)	
Length of time under current leader		
Under 6 months		57 (30.2)
Between 6 months and 1 year		56 (29.6)
Between 1 and 2 years		44 (23.3)
Between 2 and 3 years		19 (10.1)
Between 3 and 4 years		4 (2.1)
Between 4 and 5 years		4 (2.1)
Between 5 and 10 years		4 (2.1)
Over 10 years		1 (0.5)
Personnel category in organization		
Nationally recruited personnel	32 (33.3)	117 (61.9)
Internationally recruited personnel	42 (43.8)	27 (14.3)
Headquarters personnel	22 (22.9)	45 (23.8)
Organizational group		
Country programs	69 (71.9)	119 (63.0)
*Africa region*	33 (34.4)	64 (33.9)
*Asia region*	23 (24.0)	27 (14.3)
*Middle East region*	13 (13.5)	28 (14.8)
Affiliate offices	8 (8.3)	25 (13.2)
Headquarters	19 (19.8)	38 (20.1)
Other	0 (0.0)	7 (3.7)

Leader subsample *n* = 96; worker subsample *n* = 189.

#### Measures and data aggregation

Study 2 used the same measures as Study 1. Given the nested nature of the data in Study 2 [workers (level-1) within teams and leaders (level-2)], there was a need to calculate several team-aggregated mean scores at level-2. However, to justify this aggregation, building on scholarly precedent (e.g., Chiu et al., 2016, 2022), we first calculated intraclass coefficients [ICC (level-1) and ICC (level-2); Bliese, 2000] and the within-group agreement index (*r*_wg_; James et al., 1993). Recommended cutoffs for justifying aggregation are *r*_wg_ values of at least 0.70 (Bliese, 2000), ICC (1) values greater than 0.12 (James, 1982), and ICC (2) values greater than 0.25 (Dietz et al., 2015; see Chiu et al., 2016). Results of these calculations were as follows: (a) team-rated leader general humility [*M r*_wg_ = 0.76, ICC (1) = 0.26, ICC (2) = 0.41], (b) team-rated leader relational humility [*M r*_wg_ = 0.82, ICC (1) = 0.33, ICC (2) = 0.50], (c) team-rated collective humility [*M r*_wg_ = 0.80, ICC (1) = 0.25, ICC (2) = 0.39], (d) perceived leader impact on team [*M* r_wg_ = 0.76, ICC (1) = 0.21, ICC (2) = 0.34], (e) worker job engagement [*M r*_wg_ = 0.76, ICC (1) = 0.20, ICC (2) = 0.33], and (f) worker job satisfaction [*M r*_wg_ = 0.63, ICC (1) = 0.10; ICC (2) = 0.18]. In sum, results suggested aggregation was justified for all outcomes except job satisfaction. Ultimately, we decided to aggregate all variables, including job satisfaction; nonetheless, job satisfaction’s results should be interpreted with caution, given that variable’s marginally acceptable indices.

#### Analytic plan

In Study 2, we sought to test Hypotheses 1, 2, and 4. We present descriptive statistics and bivariate correlations for worker-level (level-1) variables in Supplementary Table S8 and for leaders and teams (level-2 variables) in Supplementary Table S9. However, Study 2’s primary analytic plan was to use multilevel modeling to examine the unique contribution of worker, leader, and team variables to workers’ outcomes and attributions. Because workers were nested in teams with one leader, worker variables were treated as level-1 variables, and leader and team variables were treated collectively as level-2 variables. Study 2 data were analyzed in R (version 3.6.0; R Development Core Team, 2019) using maximum likelihood estimation with the “lme4” package (Bates et al., 2015). All variables were grand-mean centered to ease interpretation and avoid multicollinearity. The variance inflation factor was examined for each model using the mer-utils.R function (Frank and O’Hara, 2014), and each factor was less than 5 (indicating a lack of multicollinearity). However, when correlations were examined more closely, we noted that several of the level-2 (L2), humility-related variables were highly intercorrelated. L2 Collective Humility was highly related both to L2 Leader Relational Humility (*r* = 0.67) and L2 Leader General Humility (*r* = 0.68). Similarly, L2 Leader Relational Humility and L2 General Humility were also highly related (*r* = 0.85). Because L2 Leader General Humility was ultimately the omnibus construct of focus in this series of studies, we used it as the sole L2 humility-related variable. Models included a random effect for intercept and fixed effects for all slopes.

We used a model building approach to test hypotheses (Hox et al., 2018). This approach involves starting with a simple model and then adding additional parameters that are tested for significance. Where parameters were significant, they were retained for use in subsequent models. Ultimately, we constructed a null model and five additional models for each outcome.

In Model 0, we ran a null model (without any predictors) to examine the percentage of variance explained by L1 and L2 predictors. In Model 1, we included control variables—worker or leader characteristics that might be related to outcomes (worker age, gender, and years in the organization; leader age, gender, and years in leadership role). In Model 2, we included workers’ self-rated general and relational humility. In Model 3, to begin testing Hypotheses 1, 2, and 4, we added leaders’ self-rated general humility, relational humility, and trait narcissism. In Model 4, we included the sole team-level variable—aggregate-mean ratings of the leader’s general humility (see the above explanation about L2 humility variables). In Model 5, to test Hypothesis 4, we included two interaction terms: Leader Narcissism x Leader General Humility and Leader Narcissism x Leader Relational Humility. Table 7 presents final models but only includes significant variables, based on a *p* < 0.05 level (due to Study 2’s lower sample size and power).

**Table 7:** Results of study 2 multilevel models examining the impact of worker-, leader-, and team-level humility variables on outcomes and attributions.

Model and variable	Leader effectiveness	Leader impact	Team cohesion	Team safety	Team performance	Job engagement	Job satisfaction	Compassion satisfaction	Burnout	Secondary trauma	Depression	PTSD	Flourishing
1: Control variables													
Worker age													
Worker gender						−0.19							
Worker length of employment	−0.16	−0.24			−0.17					−0.15			
Leader age													
Leader gender													
Leader length of time in role				0.27									
2: Worker variables													
General humility	0.22		0.29		0.30	0.45	0.31	0.50	−0.26			0.18	0.40
Relational humility		0.21		0.18					−0.34			−0.24	
3: Leader variables													
General humility	0.13	0.15											0.23
Relational humility													
Trait narcissism											−0.21	−0.15	
4: Team variables													
Ratings of leader’s general humility	0.42	0.50	0.26	0.27	0.24		0.28						
5: Interaction terms													
Leader narcissism x general humility													
Leader narcissism x relational humility													
Model variance													
σ^2^	0.68	2.02	1.19	0.72	0.38	0.83	0.85	29.51	22.27	35.16	14.63	41.20	0.66
τ_00_	0.18	0.95	0.20	0.16	0.15	0.20	0.09	14.39	3.12	14.84	2.15	0.09	0.24
% variation at L1	0.79	0.68	0.86	0.82	0.72	0.81	0.90	0.67	0.88	0.70	0.87	1.00	0.74
% variation at L2	0.21	0.32	0.14	0.18	0.28	0.19	0.10	0.33	0.12	0.30	0.13	0.00	0.26
Pseudo *R*^2^	0.30	0.39	0.24	0.14	0.31	0.27	0.18	0.30	0.27	0.33	0.04	0.09	0.19
*f* ^2^	0.43	0.64	0.32	0.16	0.45	0.37	0.22	0.43	0.37	0.49	0.04	0.10	0.23

L1, level 1 (worker-level variables); L2, level 2 (which collectively includes leader- and team-level variables). To facilitate interpretation, only values significant at the *p* < 0.05 level are included. All values indicated in the shaded rows are standardized regression coefficients (*β*).

Building on the results of Study 1, in Study 2, we focused on variables grouped into five classes: leader attributions (perceived leader effectiveness and impact), team attributions and outcomes (team cohesion, psychological safety, and performance), organizational outcomes (job engagement and satisfaction), work-related psychological outcomes (compassion satisfaction, burnout, and secondary traumatic stress), and general psychological outcomes (depression, PTSD, and psychological flourishing). Separate models were conducted for each variable, using the described analytic plan. Due to space limitations, we only report results of significant predictors from each variable’s final model (*p* < 0.05). We also report the level-1 variance estimate [which includes the estimate of sampling error (*σ*^2^)], level-2 variance estimate (τ_00_), percentage of variation attributable to level-2 factors [τ_00_/(τ_00_+ *σ*^2^)], and percentage of variance accounted for by the final model (pseudo *R*^2^), calculated based on guidelines from Lorah (2018).

### Results

Table 7 displays results of the 13 sets of multilevel models examining leader, team, organizational, work-related psychological, and general psychological variables. Of note, across all models, no interaction terms (Trait Narcissism x Leader Humility) were significant.

#### Leader attributions

We tested two leader attributions: perceived leader effectiveness and leader impact on team effectiveness. Perceived leader effectiveness was related to (a) workers’ shorter length of employment [*γ* = −0.08, *SE* = 0.03, *t* (91) = −2.59, *p* = 0.011, *β* = −0.16], (b) workers’ higher self-rated general humility [*γ* = 0.46, *SE* = 0.13, *t* (91) = 3.51, *p* < 0.001, *β* = 0.22], (c) leaders’ higher self-rated general humility [*γ* = 0.25, *SE* = 0.12, *t* (93) = 2.10, *p* = 0.039, *β* = 0.13], and (d) teams’ higher aggregated leader general humility [*γ* = 0.41, *SE* = 0.06, *t* (93) = 6.78, *p* < 0.001, *β* = 0.42]. Perceived leader impact on team effectiveness was related to: (a) workers’ shorter length of employment [*γ* = −0.20, *SE* = 0.05, *t* (91) = −3.99, *p* < 0.001, *β* = −0.24], (b) workers’ higher self-rated relational humility [*γ* = 0.77, *SE* = 0.21, *t* (91) = 3.50, *p* < 0.001, *β* = 0.21], (c) leaders’ higher self-rated general humility [*γ* = 0.54, *SE* = 0.21, *t* (91) = 2.57, *p* < 0.001, *β* = 0.15], and (d) teams’ higher aggregated leader general humility [*γ* = 0.91, *SE* = 0.11, *t* (91) = 8.19, *p* < 0.001, *β* = 0.50].

When comparing standardized values of all variables, a team’s aggregated rating of their leader’s general humility made the strongest contribution to positive leader attributions, both for perceived leader effectiveness and team impact (*β*s = 0.42 and 0.50, respectively), and leaders’ and workers’ self-attributions of humility made small contributions (*β*s = 0.13–0.22). Each model accounted for a large amount of variance in leader attributions [pseudo *R*^2^s = 0.30 and 0.39 (*f* ^2^ = 0.43 and 0.64)]. Worker-, leader-, and team-level humility variables each contributed to positive leader attributions, with the strongest contribution from teams’ shared attributions of leader humility.

#### Team attributions and outcomes

We tested two team attributions (team cohesion and psychological safety) and one team outcome (performance). Team cohesion was related to (a) workers’ higher self-rated general humility [*γ* = 0.78, *SE* = 0.19, *t* (91) = 4.17, *p* < 0.001, *β* = 0.29] and (b) teams’ higher aggregated leader general humility [*γ* = 0.32, *SE* = 0.08, *t* (93) = 3.97, *p* < 0.001, *β* = 0.26]. Team psychological safety was related to (a) leaders’ greater length of time in their role [*γ* = 0.13, *SE* = 0.04, *t* (93) = 3.47, *p* < 0.001, *β* = 0.27], (b) workers’ higher self-rated relational humility [*γ* = 0.36, *SE* = 0.13, *t* (92) = 2.75, *p* = 0.007, *β* = 0.18], and (c) teams’ higher aggregated leader general humility [*γ* = 0.26, *SE* = 0.07, *t* (93) = 3.84, *p* < 0.001, *β* = 0.27]. Team performance was related to (a) workers’ shorter length of employment [*γ* = −0.06, *SE* = 0.02, *t* (91) = −2.67, *p* = 0.009, *β* = −0.17], (b) higher self-rated general humility [*γ* = 0.50, *SE* = 0.11, *t* (91) = 4.46, *p* < 0.001, *β* = 0.30] and (c) teams’ higher aggregated leader general humility [*γ* = 0.18, *SE* = 0.05, *t* (93) = 3.53, *p* < 0.001, *β* = 0.24).

In sum, results indicated team-level variables (higher aggregated leader humility) and worker-level variables (worker self-rated humility) were the best predictors of team attributions and outcomes. Models explained a medium to large portion of variation [pseudo *R*^2^s = 0.14–0.31 (*f*^2^s = 0.16–0.45)], with similarly sized contributions at the team and worker levels but no meaningful contribution at the leader level. That is, workers’ self-attributions of humility and team’s shared attributions of leader humility predicted positive team attributions and outcomes.

#### Organizational outcomes

We tested two organizational outcomes: job engagement and job satisfaction. Job engagement was related to (a) workers’ male gender identification [*γ* = −0.38, *SE* = 0.13, *t* (91) = −2.91, *p* = 0.005, *β* = −0.19] and (b) workers’ higher self-rated general humility [*γ* = 1.05, *SE* = 0.15, *t* (91) = 7.13, *p* < 0.001, *β* = 0.45]. Job satisfaction was related to (a) workers’ higher self-rated general humility [*γ* = 0.73, *SE* = 0.15, *t* (92) = 4.89, *p* < 0.001, *β* = 0.31] and (b) teams’ higher aggregated leader general humility [*γ* = 0.23, *SE* = 0.07, *t* (92) = 3.38, *p* = 0.001, *β* = 0.28].

Consistent with Hypothesis 1, results indicated teams’ shared attributions about their leader’s humility contributed to workers’ job satisfaction. Results also indicated workers’ self-attributions of humility contributed to better job satisfaction and engagement. Models explained medium-to-large variance in outcomes [pseudo *R*^2^s = 0.27 and 0.18 (*f* ^2^s = 0.37 and 0.22)], with similarly sized contributions at the worker and team levels but no contribution at the leader level.

#### Work-related psychological outcomes

We measured three work-related psychological outcomes: compassion satisfaction, burnout, and secondary traumatic stress. Compassion satisfaction was only related to workers’ higher self-rated general humility [*γ* = 7.67, *SE* = 0.97, *t* (92) = 7.93, *p* < 0.001, *β* = 0.50]. Burnout was only related to workers’ lower self-rated general humility [*γ* = −3.02, *SE* = 0.78, *t* (91) = −3.87, *p* < 0.001, *β* = −0.26] and relational humility [*γ* = −3.61, *SE* = 0.71, *t* (91) = −5.09, *p* < 0.001, *β* = −0.34]. Secondary traumatic stress was related only to workers’ shorter length of employment [*γ* = −0.74, *SE* = 0.25, *t* (91) = −2.96, *p* = 0.004, *β* = −0.15]. These three models explained a medium-to-large portion of variance in work-related psychological outcomes [pseudo *R*^2^s = 0.27 to 0.33 (*f* ^2^s = 0.16–0.45)]. In sum, these models indicated aid workers’ self-attributions of their own humility made the strongest contribution to their work-related psychological outcomes. Hypotheses 1 and 2 were unsupported in that neither teams’ aggregated ratings of leader humility nor leaders’ self-rated humility predicted work-related psychological outcomes.

#### General psychological outcomes

Based on Study 1 results, we only tested three general psychological outcomes among workers: depression symptoms, PTSD symptoms, and psychological flourishing. Workers’ depression symptoms were only related (unexpectedly) to lower leader self-rated narcissism [*γ* = −0.31, *SE* = 0.11, *t* (94) = −2.76, *p* < 0.001, *β* = −0.21]. Also unexpectedly, workers’ PTSD symptoms were related to (a) leaders’ lower self-rated narcissism [*γ* = −0.35, *SE* = 0.17, *t* (94) = −2.06, *p* = 0.042, *β* = −0.15], (b) workers’ higher self-rated general humility [*γ* = 2.62, *SE* = 1.12, *t* (91) = 2.33, *p* = 0.022, *β* = 0.18], and (c) workers’ lower self-rated relational humility [*γ* = −3.23, *SE* = 1.02, *t* (91) = −3.18, *p* = 0.002, *β* = −0.24]. Even so, as expected, workers’ flourishing was related to higher worker self-rated general humility [γ = 0.87, *SE* = 0.14, *t*(92) = 6.07, *p* < 0.001, *β* = 0.40] and leader self-rated general humility [*γ* = 0.45, *SE* = 0.14, *t* (94) = 3.14, *p* = 0.002, *β* = 0.23].

In sum, results indicated workers’ and leaders’ self-attributions of humility are tied most reliably to workers’ general psychological outcomes, but these associations are rather complex. Workers’ and leaders’ self-attributions of humility were related to higher worker flourishing, and workers’ self-rated relational humility was related to lower worker PTSD symptoms. However, workers’ self-rated general humility was also associated with higher PTSD symptoms, and consistent with Hypothesis 4, leaders’ self-rated narcissism was related to lower worker PTSD and depression symptoms. These three models explained a small-to-medium amount of variance in outcomes [pseudo *R*^2^s = 0.04 and 0.19 (*f*^2^s = 0.04 to 0.23)], with small-to-moderate contributions from worker and leader self-attributions but no contribution from teams’ shared attributions.

### Discussion

Several Study 2 findings warrant elaboration. First, Hypotheses 1 and 2 were partially supported in that teams’ aggregated ratings of leader humility and leaders’ self-rated humility were related to many positive attributions and outcomes. Teams’ shared perceptions of their leader’s humility were related to positive leader attributions (of perceived leader effectiveness and impact on team effectiveness), as were leaders’ self-attributions of humility. Team’s shared perceptions of their leader’s humility were also linked to positive attributions about their team (as being cohesive and psychological safe) and to higher worker job satisfaction and team performance. Taken together, there was robust support for our project’s conceptual model (Figure 1) and the positive attribution (Qin et al., 2020) and social contagion hypotheses (Owens and Hekman, 2016) that informed it. Study 2 offered evidence of a multilevel attributional social contagion that happens in humanitarian aid teams with humble leaders. Consistent with what Li et al. (2019) found in a study of information technology firms in China, humanitarian aid teams that have humble leaders may develop shared mental models that define and drive their work. The workers and leaders who comprise these teams develop shared positive attributions about their leaders, team, and themselves, and those positive attributions give them a sense of satisfaction in their work and pride in their team and its effectiveness. It may even be that the psychobiological model of personality (Cloninger et al., 1993) can be applied at the team level, in that aid teams led by virtuous leaders come to define their collective character as virtuous, and that virtuousness then helps inspire and motivate their work, which in turn supports the positive functioning and effectiveness of their team and its members. Perhaps aid teams that experience their leader as virtue-driven become virtue-driven teams, comprised of virtue-driven personnel. Virtuousness becomes a defining characteristic of their team and driving motivation of its work.

Yet, when it comes to this virtuousness, Study 2 suggests workers’ views of their own virtuousness may influence their organizational and psychological outcomes more so than views of their leader’s virtuousness. Workers’ personal humility was related to most outcomes—lower burnout and higher job engagement, job satisfaction, compassion satisfaction, and flourishing. In contrast, the only outcomes related to team-aggregated leader humility were job satisfaction and team performance. Worker ratings of leader humility were unassociated with workers’ professional quality of life or mental health; instead, worker and leader self-attributions usually contributed. Consistent with our conceptual framework, views of leader virtuousness may contribute more indirectly to outcomes via their contribution to positive multilevel attributions.

As predicted by our Hypothesis 4, such contributions to outcomes seem especially complex when it comes to humility and narcissism. Consistent with the humility–narcissism paradox, workers’ general psychological outcomes exhibited some paradoxical associations. Although there was no interaction of leader humility and narcissism in jointly predicting any outcomes, leaders’ self-rated humility was tied to higher worker flourishing, whereas leaders’ self-rated narcissism was tied to lower worker PTSD and depression symptoms. It may be that in humanitarian aid work, certain humble or narcissistic leader behaviors might be health-enhancing in some situations and cultural contexts but health-undermining in others. It may also be that the interactions between leader narcissism and humility are more culturally bound and complex than existing theories and measures of these constructs suggest (Wetzel et al., 2021). Cross-cultural studies are vitally needed to explore these nuances and complexities.

Finally, it is worth highlighting that around 20% of variation in workers’ organizational and psychological outcomes was due to team−/leader-level factors (the L2 row in Table 7). That contribution is quite meaningful and underscores the need for multilevel research. Studies that only look at the individual level of analysis (L1) will not provide a full enough picture of how leader, worker, and team factors interact in predicting outcomes among humanitarian personnel.

## Study 3: the longitudinal consequences of leader and team humility

Study 1 examined the correlates of leader and team humility, and Study 2 explored their multilevel contributions to several worker and leader outcomes. Study 3 relied on a subsample to test what kind of consequences leader and team humility might have on those same outcomes. Study 2 had focused on two levels of analysis (individual and team), but Study 3 mirrored Study 1 in the sense that we returned to focusing solely on the individual level of analysis—first at workers’ ratings of all variables and then at leaders’ ratings of all variables.

### Method

The Study 3 subsample consisted of the 50 workers and 34 leaders who completed the same survey at T1 and T2. Eleven of them completed both versions, so the Study 3 response rate was 73/644 (11.34%). See Table 8 for demographics of workers (*M*_age_ = 35.30, *SD* = 9.27) and leaders (*M*_age_ = 37.97, *SD* = 11.52). Study 3 used the same measures as Studies 1 and 2.

**Table 8:** Demographic characteristics of the study 3 sample at time 1.

Characteristic	Leaders	Workers
	*n* (%)	*n* (%)
Gender identity		
Male	18 (52.9)	22 (44.0)
Female	16 (47.1)	28 (56.0)
Nationality		
Afghan	2 (5.9)	2 (4.0)
American	5 (14.7)	3 (6.0)
British	4 (11.8)	3 (6.0)
Canadian	2 (5.9)	4 (8.0)
Congolese	1 (2.9)	0 (0.0)
Dutch	4 (11.8)	4 (8.0)
French	1 (2.9)	1 (2.0)
German	1 (2.9)	1 (2.0)
Iraqi	4 (11.8)	7 (14.0)
Jordanian	0 (0.0)	0 (0.0)
Kenyan	1 (2.9)	0 (0.0)
Lebanese	2 (5.9)	6 (12.0)
Somalian	1 (2.9)	9 (18.0)
South Sudanese	0 (0.0)	3 (6.0)
Swiss	2 (5.9)	4 (8.0)
Other	4 (11.8)	3 (6.0)
Length of time at organization		
Under 6 months	1 (2.9)	5 (10.0)
Between 6 months and 1 year	4 (11.8)	11 (22.0)
Between 1 and 2 years	8 (23.5)	10 (20.0)
Between 2 and 3 years	8 (23.5)	11 (22.0)
Between 3 and 4 years	3 (8.8)	5 (10.0)
Between 4 and 5 years	2 (5.9)	4 (8.0)
Between 5 and 10 years	7 (20.6)	4 (8.0)
Over 10 years	1 (2.9)	0 (0.0)
Length of time in current leader role		
Under 6 months	7 (20.6)	
Between 6 months and 1 year	8 (23.5)	
Between 1 and 2 years	7 (20.6)	
Between 2 and 3 years	7 (20.6)	
Between 3 and 4 years	2 (5.9)	
Between 4 and 5 years	1 (2.9)	
Between 5 and 10 years	1 (2.9)	
Over 10 years	1 (2.9)	
Length of time under current leader		
Under 6 months		11 (22.0)
Between 6 months and 1 year		18 (36.0)
Between 1 and 2 years		19 (38.0)
Between 2 and 3 years		1 (2.0)
Between 3 and 4 years		0 (0.0)
Between 4 and 5 years		1 (2.0)
Between 5 and 10 years		0 (0.0)
Over 10 years		0 (0.0)
Personnel category in organization		
Nationally recruited personnel	12 (35.3)	23 (46.0)
Internationally recruited personnel	15 (44.1)	9 (18.0)
Headquarters personnel	7 (20.6)	18 (36.0)
Organizational group		
Country programs	25 (73.5)	23 (46.0)
*Africa region*	8 (23.5)	13 (26.0)
*Asia region*	3 (8.8)	3 (6.0)
*Middle East region*	14 (41.2)	7 (14.0)
*Other*	0 (0.0)	0 (0.0)
Affiliate offices	2 (5.9)	12 (24.0)
Headquarters	7 (20.6)	15 (30.0)

Leader subsample *n* = 34; worker subsample *n* = 50.

Study 3 tested Hypotheses 3 and 4. To do so, we used outcome-wide analyses (VanderWeele et al., 2020) and regression-based moderation analyses (Hayes, 2022).

### Results

Results of outcome-wide longitudinal analyses for Study 3 leaders (above the diagonal) and workers (below the diagonal) are presented in Table 9. For each partial correlation, we controlled for T1 scores on the applicable outcome and for applicable covariates (team cohesion, team psychological safety, and worker and leader sociodemographics), because doing so helped rigorously isolate the contribution of the T1 humility variable to the respective T2 outcome. Given the low sample size and rigorous control of covariates, we used a significance level of *p* < 0.05 for all analyses. Among workers, T1 worker-rated leader humility was associated with workers’ higher job satisfaction, lower secondary traumatic stress, and (unexpectedly) lower job engagement 6 months later. Workers’ self-rated relational humility was also associated with higher job satisfaction at T2, and worker-rated team collective humility was linked to higher worker job engagement and psychological flourishing at T2. Among leaders, leaders’ self-rated general humility at T1 was associated with higher leader job engagement at T2, and leader-rated team collective humility at T1 was related to higher leader-rated team performance 6 months later (T2). Nonetheless, leaders’ self-rated relational humility at T1 was related unexpectedly to lower compassion satisfaction at T2. Taken together, results partially supported Hypothesis 3.

**Table 9:** Partial correlations between T1 humility variables and T2 outcomes, controlling for t1 outcome scores and covariates.

Variable	1	2	3	4	5	6	7	8	9	10	11	12	13	14	15	16	17
1. T1 leader general humility	--	--	--	--	--	0.47^*^	−0.09	0.18	0.31†	−0.06	−0.03	0.08	0.25	0.27†	0.24	0.28†	0.08
2. T1 leader relational humility	--	--	--	--	--	0.00	0.11	−0.04	0.08	0.09	−0.28†	−0.03	−0.04	0.07	−0.33^*^	−0.05	−0.24
3. T1 worker general humility	--	--	--	--	--	--	--	--	--	--	--	--	--	--	--	--	--
4. T1 worker relational humility	--	--	--	--	--	--	--	--	--	--	--	--	--	--	--	--	--
5. T1 team collective humility	--	--	--	--	--	0.14	−0.03	0.30†	0.40^*^	−0.01	0.00	−0.02	0.18	0.07	0.18	−0.13	0.29†
6. T2 job engagement	−0.26^*^	−0.30^*^	0.11	0.01	0.26^*^												
7. T2 job satisfaction	0.34^*^	0.37^*^	−0.07	0.27^*^	−0.03												
8. T2 worker performance	--	--	--	--	--												
9. T2 team performance	0.23†	0.18	0.17	0.24†	−0.13												
10. T2 depression symptoms	−0.06	−0.02	−0.01	−0.01	0.02												
11. T2 anxiety symptoms	0.23†	0.18	0.08	0.12	0.04												
12. T2 PTSD symptoms	−0.06	0.02	0.15	0.01	0.14												
13. T2 secondary trauma	−0.45^*^	−0.33^*^	0.07	−0.13	−0.09												
14. T2 burnout	−0.14	−0.04	0.03	−0.09	−0.19												
15. T2 compassion satisfaction	−0.15	−0.20	0.21†	−0.01	0.18												
16. T2 perceived PTG	−0.05	0.06	0.04	0.12	0.19												
17. T2 flourishing	−0.10	−0.15	0.11	0.12	0.26^*^												

T1, Time 1; T2, Time 2; PTSD, post-traumatic stress disorder; PTG, posttraumatic growth. Partial correlations for leaders (*N* = 34) are presented above the diagonal and for workers (*N* = 50) are presented below the diagonal. Partial correlations control for T1 scores on the applicable organizational or psychological outcome, as well as T1 scores on the applicable covariates (team cohesion, team psychological safety, and worker and leader sociodemographics). All partial correlation coefficients that are significant at *p < 0.05 (one-tailed) are presented in boldface and italicized type.

Moderation analyses revealed no significant interaction effects based on leader narcissism, failing to support Hypothesis 4. That replicated the null interaction effects in Study 2.

### Discussion

Study 3 builds on Studies 1 and 2 in important ways. First, it gives initial longitudinal evidence that leader, worker, and team humility may have some occupational and psychological effects. For example, when aid workers perceive their leader and themselves as humble, they might report higher job satisfaction over time. Aid workers who view their leader as humble may struggle a bit to maintain high job engagement, perhaps because they mistake their leader’s humility as a license for them not to have to work as hard. However, if they perceive their team as collectively humble, it might help workers stay engaged with their job and experience enhanced psychological flourishing over time. In addition, leader humility may enhance aid worker psychological well-being indirectly by helping buffer against secondary traumatic stress.

Results for leaders were more mixed. Aid leaders who work on humble teams may have teams that exhibit better performance over time, perhaps because their team’s other-orientedness and teachability enhances their aid effectiveness. Similarly, leaders who view themselves as humble seem especially able to maintain high job engagement over time, maybe partly because of how outward- and other-oriented they are. At the same time, this other-orientedness might place humble aid leaders at risk for experiencing decreased compassion satisfaction over time.

## General discussion

Taken together, results from these three studies suggest leader and team humility contribute to several organizational and psychological outcomes among humanitarian aid workers and leaders. Leader and team humility particularly contribute to aid workers’ job satisfaction, which is a finding that has emerged consistently across other organizational contexts (Owens et al., 2013; Ou et al., 2017; Zhong et al., 2020). Aid workers may appreciate and benefit from the other-orientedness that humble leaders and teams embody, and this other-orientedness might help bolster their job satisfaction amid the stresses and challenges of humanitarian work. In the normatively difficult aid context, other-oriented leaders and teams may help offer vitally needed social and organizational support that can prevent mental health problems that are common among humanitarian aid personnel (Eriksson et al., 2009; Prati and Pietrantoni, 2010).

Leader and team humility were also consistently tied to a sense of team cohesion, safety, and collective promotion focus (growth-mindedness). Humble humanitarian aid leaders and teams may be uniquely skilled at providing effective humanitarian aid because they are so other-oriented and teachable. Humble aid leaders and teams might routinely look outwardly for ways to serve more effectively and routinely look inwardly for ways to grow and improve. This combination of inward-facing growth-mindedness and outward-facing teachability and generosity may play a crucial role in effective humanitarian aid. If so, then leaders and teams who foster this combination of humble skills and attitudes can enhance the effectiveness of their humanitarian assistance, as humanitarian exemplars (Shannonhouse et al., 2019) and public health experts (Harvard School of Public Health, 2012) suggest. Future research could examine this possibility, ideally measuring both objective and subjective multilevel outcomes (U.S. Department of State, 2009).

One key finding from these studies was the positive attribution (Qin et al., 2020) and social contagion hypotheses (Owens and Hekman, 2016) of leader humility were robustly supported. For aid workers, perceived leader humility was reliably associated with positive attributions about their leader, team, and themselves. This finding emerged at the individual and aggregated team levels. It also emerged from leaders’ perspective in that leaders’ self-rated humility was consistently tied both to leaders’ positive attributions about their team and to leaders’ own occupational and psychological health. Taken together, it seems humility might operate mainly as an attributional social contagion that spreads among workers, teams, and leaders, and any health benefits leader humility has may primarily occur indirectly via this multilevel positive attributional style that it fosters. These multilevel attributions may become shared mental models (Li et al., 2019) that aid workers, teams, and leaders use to define themselves, their team, and their work. Team members may come to define themselves as humanitarian aid personnel who are virtue-driven and serve in virtue-driven teams led by virtue-driven leaders who work at virtue-driven aid organizations. It is possible the same types of interrelated virtue profiles that characterize the most virtuous and flourishing individuals (Moreira et al., 2021) come to characterize the most virtuous, flourishing, and effective humanitarian aid teams and organizations. Perhaps the most flourishing and effective humanitarian teams are characterized by a shared humility and a broader shared virtuousness, cohesiveness, and growth-mindedness that inspires and motivates how they relate to each other, approach their aid work, and define themselves collectively and individually. Future studies with larger samples and more complex longitudinal designs could test this idea.

In the meantime, our Study 3 results offer initial longitudinal evidence that, within humanitarian aid organizations, teams’ collective humility may lead to improved team performance over time, consistent with evidence from traditional organizational contexts (Ou et al., 2015). The communal embodiment of humility may cultivate a type of relational harmony, distributed responsibility, and other-oriented benevolence that enhances the performance and flourishing of humanitarian aid teams and organizations. Such a possibility is convergent with prior research on romantic couples, which has found that humility was positively related to psychological and physical health after a stressful life event but only when one’s partner was also high in humility (Van Tongeren et al., 2019b). The benefits of humility are likely richest in relationships in which all members are humble (Worthington et al., 2017), and this is probably true in humanitarian aid teams and organizations as well. Even as individuals who exhibit the highest levels of integration among their temperament, character, and virtues may be the healthiest and most flourishing (Moreira et al., 2021), the humanitarian teams that exhibit the highest integration of humility and other virtues at the leader, worker, and team levels might flourish and function the best.

### Strengths and limitations

Collectively, these studies extend extant research by using a multimethod and multilevel strategy to assess humility (i.e., a combination of self-report, informant report, and aggregate reports) and by using a diverse international sample and longitudinal design. These studies are among the first studies of leader humility conducted in Africa and the Middle East and among the first studies of humility conducted in the humanitarian aid sector. In addition, these studies introduced Arabic- and French-language versions of 38 well-utilized English-language measures.^8^

At the same time, these studies have noteworthy limitations, and their results should be interpreted accordingly. First, great care was taken to measure all constructs validly, but the assessment of humility can be plagued by response biases. For instance, people who are more other-focused (i.e., humble) may rate themselves as lower in humility, whereas people who are more self-focused may tend to overestimate their humility (Worthington et al., 2017). Although we included both self- and other-report ratings of leader humility, future research could evaluate other-reported humility more robustly by assessing the same leader from several workers, which was not as possible in these studies due to the relatively few workers that each leader supervised. Similarly, we used mostly self-report measures of organizational and psychological outcomes. Naturally, these measures can also be plagued by response biases (e.g., under- or overreporting), and their measured constructs can be influenced by numerous confounding variables (biological, personality, social, and cultural contributors). To address omitted variable bias, we controlled for several potential confounds, but there of course are others. For example, future research could use the self- and peer-report HEXACO Personality Inventory (Lee and Ashton, 2008) to assess the nomologically related traits of honesty–humility and agreeableness (Chandler et al., 2023).

Low response rates were another significant limitation of this project. Although Study 1 had a 65% response rate, the response rates for Study 2 (44%) and Study 3 (11%) were much closer to what is typical in humanitarian aid research (~10 to 20%; Ehrenreich and Elliott, 2004; Strohmeier et al., 2018). Footnote 2 suggests many plausible reasons for that. To increase survey responsiveness, future researchers can work with humanitarian aid agencies to communicate with their personnel about compelling, concrete ways the survey will benefit the aid organization, its personnel, and its aid beneficiaries. They can produce introductory materials that inspiringly convey the survey’s value for advancing the organization’s mission and for enhancing its humanitarian effectiveness. They also can take time during scheduled meetings to have personnel complete the study surveys (to communicate the organization places a high value on survey participation) and then use subsequent meetings to present and discuss the survey results and their applications.

In addition, future longitudinal research on humanitarian leader humility could gather more waves, sources, and types of data, perhaps even utilizing experience sampling methodology that would more closely link leader and team humility with real-time organizational and psychological outcomes. Linking leader, worker, and team humility with beneficiary outcomes is another promising avenue. Finally, because the current study was only conducted in Africa, the Middle East, and Asia, humility studies in other cultural contexts of humanitarian aid are needed.

## Conclusion

For decades, scientific research has supported the benefits of humility generally (Worthington et al., 2017; Van Tongeren et al., 2019a) and leader humility specifically (Appendix S1 and Supplementary Table S1, S2; see also Chandler et al., 2023). Our findings advance this research and suggest leader and team humility may play a meaningful role in humanitarian aid organizations as well. These studies indicate that leader and team humility may facilitate the optimal functioning and flourishing of humanitarian aid workers, teams, and leaders. It seems to play a particularly strong role in fostering a positive attributional style, a sense of team cohesion, and a collective growth-mindedness. Leader humility may catalyze not only this attributional social contagion but also a motivational social contagion of virtuousness and growth-mindedness. This constellation of virtuous habits, goals, and values may help humanitarian aid personnel flourish even amidst extremely challenging humanitarian situations. Humility and other virtues can help them nourish a sense of shared meaning, purpose, and vision as they work to meet urgent humanitarian needs.

Given the practical import of this relational virtue, we hope that humanitarian aid organizations and their leaders will increasingly prioritize humility and character virtue development across organizational levels. We also hope that humanitarian organizations worldwide will place humility and virtuousness at the center of how aid leaders and workers are selected, trained, and supported. In doing so, hopefully the lives of the growing portion of global population who need humanitarian assistance will be improved.

## Data availability statement

This study was preregistered with the Open Science Framework (https://osf.io/gtu9h/), and the dataset and materials are openly available at this link.

## Ethics statement

The studies involving humans were approved by the Wheaton College Institutional Review Board. The studies were conducted in accordance with the local legislation and institutional requirements. The participants provided their written informed consent to participate in this study.

## Author contributions

ED: conceptualization, data curation, formal analysis, funding acquisition, investigation, methodology, project administration, resources, supervision, validation, visualization, writing – original draft, and writing – review & editing. KB: investigation, methodology, project administration, resources, supervision, writing – original draft, and writing – review & editing. JA: conceptualization, funding acquisition, investigation, methodology, writing – original draft, and writing – review & editing. LS: conceptualization, funding acquisition, investigation, methodology, writing – original draft, and writing – review & editing. DW: conceptualization, funding acquisition, formal analysis, methodology, writing – original draft, and writing – review & editing. DT: conceptualization, funding acquisition, investigation, methodology, writing – original draft, and writing – review & editing. DD: conceptualization, funding acquisition, investigation, methodology, writing – original draft, and writing–review & editing. JH: conceptualization, funding acquisition, investigation, methodology, writing – original draft, and writing–review & editing. ZC and GL: formal analysis, methodology, writing – original draft, and writing – review & editing. SM-H: methodology, writing – original draft, and writing – review & editing. EE: data curation, formal analysis, writing – original draft, and writing – review & editing. LG: data curation, writing – review & editing, and project administration. EL: data curation, writing – original draft, writing – review & editing, and project administration. TB: data curation, writing – review & editing, and project administration. PS, SO, and KS: writing – review & editing. All authors contributed to the article and approved the submitted version.

## Footnotes

^1^Nationally recruited humanitarian aid personnel are work-eligible personnel who are recruited from the country where the response is taking place and are a citizen or permanent resident of that country. Internationally recruited personnel are work-eligible personnel recruited from outside the country where the response is taking place and who are a citizen or permanent resident of another country.

^2^ https://osf.io/cvydq/files/osfstorage

^3^There are many reasons why conducting research in humanitarian contexts is often challenging. For example, the nature of humanitarian assistance involves saving lives (by enhancing food security, water access, water sanitation, medical care, etc.), alleviating suffering (by helping people escape or navigate harsh conditions such as persecution, war, disasters, crises, etc.), and promoting human dignity (by offering education, disaster spiritual and emotional care, etc.). These contexts can be demanding and draining, often leading to high rates of overworking, role overfunctioning, burnout, and turnover. Aid personnel frequently feel they are stretched thinly and falling short (Shannonhouse et al., 2019), so they may often feel that investing time and energy into research participation is not worth the cost of drawing them away from their direct aid work, especially considering their already-limited resources. It may be difficult to recognize how research can be harnessed to enhance their aid work’s effectiveness.

^4^It is worth noting that all this study’s outcomes could of course be influenced by other important factors that are unrelated to humanitarian aid work. Such factors include the respondent’s mental health history (e.g., previous depressive and anxious episodes), history of exposure to potentially traumatic events unrelated to humanitarian aid work, and genetically and neurobiologically based temperament (Cloninger et al., 1993, 2019).

^5^*Narcissism* is a personality trait characterized by “‘a grandiose yet fragile sense of self as well as a preoccupation with success and demands for admiration’ (Ames et al., 2006, p. 441) and… self-absorption, extreme confidence or superiority, exploitiveness/entitlement, and a strong drive to lead” (Owens et al., 2015, p. 1203). Other traits such as leader cooperativeness, self-transcendence, spirituality, or empathy can interact with leader humility and its associated outcomes (Chandler et al., 2023). We focus on narcissism because of replicated evidence supporting the leader humility–narcissism paradox (Owens et al., 2015; Swain and Korenman, 2018).

^6^Of course, given the cross-sectional nature of the data, it is also possible experiencing secondary traumatic stress may contribute to leaders viewing themselves more humbly, or a third variable may account for this relationship.

^7^We also explored if rate frequencies differed based on (a) leader status (leader or worker), (b) gender identity (male or female), (c) personnel category (nationally recruited, internationally recruited, or headquarters), or (d) region of fieldwork (Africa, Asia, or the Middle East; see Supplementary Table S7). No chi-squared tests were significant at *p* < 0.05.

^8^Although these Arabic- and French-language versions were meticulously translated, back-translated, and field-checked, they only have been introduced here in this article. These versions have not yet been specifically validated because of how few participants chose to use them in this series of studies. Therefore, future studies are needed to investigate evidence of these measures’ reliability, validity, measurement invariance, and cross-cultural equivalence.
