# Supplementary material for: The multilevel correlates, contributions, and consequences of leader humility in humanitarian aid work
Source: Front Psychol. 2023 Dec 13;14:1188109. doi: 10.3389/fpsyg.2023.1188109 (PMC10751791; doi:10.3389/fpsyg.2023.1188109)
Supplement: Supplementary file 1 [file Data_Sheet_1.PDF]

The Multilevel Correlates, Contributions, and Consequences of Leader Humility in Humanitarian Aid Work

Edward B. Davis[1*], Kelly Barneche[2], Jamie D. Aten[3], Laura R. Shannonhouse[4], David C. Wang[5], Daryl R. Van Tongeren[6], Don E. Davis[4], Joshua N. Hook[7], Zhuo Job Chen[8], G. Tyler Lefevor[9], Stacey E. McElroy-Heltzel[10], Emilie L. Elick[1], Leif Van Grinsven[1], Ethan K. Lacey[1], Tyler R. Brandys[1], Philip K. Sarpong[1], Sophia A. Osteen[1], and Kati Shepardson[1]

Author Note

[1]School of Psychology, Counseling, and Family Therapy, Wheaton College, Wheaton, IL, USA
[2]Medair, Ecublens, Switzerland
[3]Humanitarian Disaster Institute, Wheaton College, Wheaton, IL, USA
[4]Department of Counseling and Psychological Services, Georgia State University, Atlanta, GA, USA
[5]School of Psychology and Marriage and Family Therapy, Fuller Theological Seminary, Pasadena, CA, USA
[6]Psychology Department, Hope College, Holland, MI, USA
[7]Psychology Department, University of North Texas, Denton, TX, USA
[8]School of Nursing, University of North Carolina at Charlotte, Charlotte, NC, USA
[9]Department of Psychology, Utah State University, Logan, UT, USA
[10]Department of Psychological and Quantitative Foundations, University of Iowa, Iowa City, IA, USA

This study was registered with the Open Science Framework (https://osf.io/gtu9h/); the dataset and materials are openly available at this link. This research was funded by Grants #60622 and #61865 from the John Templeton Foundation. The opinions expressed in this manuscript are those of the authors and do not necessarily reflect the views of the John Templeton Foundation. The authors declared no potential conflicts of interest with respect to the research, authorship, and publication of this manuscript.

*Correspondence concerning this manuscript should be addressed to Edward B. Davis, School of Psychology, Counseling, and Family Therapy, Wheaton College, 501 E College Ave, BGC M242, Wheaton, IL 60187. E-mail: ward.davis@wheaton.edu

Appendix S1

A Brief Systematic Review of the Existing Empirical Research on Leader Humility

Scholarly interest in leader humility has accelerated over the last decade, as evidence of its benefits has accrued. To summarize these benefits, we conducted a systematic review of the existing empirical research on leader humility. On June 27, 2022, we conducted a search in the APA PsycInfo database, using the search terms "leader humility" OR "expressed humility" and the limiters "Academic Journals" and "Empirical Study." This search yielded 65 peer-reviewed empirical articles (95 separate studies) on leader humility (for the list, see https://osf.io/gtu9h/).

The earliest of these was Owens and Hekman's (2012) seminal qualitative study that developed a theory of the behaviors, contingencies, mechanisms, and outcomes of humble leadership. Based on thematic analysis of interviews with 55 leaders from several organizational contexts (e.g., mortgage banking, information technology, healthcare, religious communities, and the military), this sample of leaders indicated the main outcomes of leader humility were increased (a) relational trust, loyalty, and satisfaction; (b) psychological freedom; (c) job engagement; (d) preference for small, continuous organizational change; and (e) fluid organizing (i.e., "ease and swiftness in transitioning to different ways of functioning," p. 802). Over the past decade, the 64 other peer-reviewed leader-humility articles we found (summarized in Table S1) have largely supported Owens and Hekman's (2012) model, even as many additional outcomes linked to leader humility[1] have been identified. These outcomes generally fall into three categories: organizational, team, and psychological outcomes (see Table S2 for a summary).

---

[1] Most of the 95 empirical studies of leader humility have been time-lagged (k = 40, 42.1%) or cross-sectional (k = 29, 30.5%). Only 21 studies (22.1%) have been experimental (most with online-crowdsourced samples or student samples), and just 5 studies (5.3%) have been longitudinal, three of them with undergraduate samples (Krumrei-Mancuso, 2018; Owens et al., 2013; Owens & Hekman, 2016, Study 2), one with a sample of religious leaders (Cuthbert et al., 2018), and one with a sample of employees in a multinational health services organization (Owens & Hekman, 2016, Study 3). Hence, it perhaps is most appropriate to summarize the existing research as indicating what outcomes are *correlated* with leader humility, rather than what outcomes are *caused* by leader humility.

Organizational Outcomes

Leader humility is related to better follower job performance (Bin et al., 2021; Diao et al., 2019; L. Wang et al., 2018), engagement (Diao et al., 2019; X. Li et al., 2021; Nguyen et al., 2020), and satisfaction (Ou et al., 2017; Zhong et al., 2020). It is also linked to (a) lower turnover (Ou et al., 2017; Owens et al., 2013); (b) higher follower organizational-citizenship (adaptive extrarole behavior; Nguyen et al., 2020), prosocial (Carnevale et al., 2019; Owens et al., 2019), and ethical behaviors (Naseer et al., 2020; Owens et al., 2019); and (c) higher follower commitment to their organization (X. Wang et al., 2022) and perceived organizational support (Yuan et al., 2018). Followers identify more strongly with leaders they think are humble (Carnevale et al., 2019) and view humble leaders as more competent (Cojuharenco & Karelaia, 2020), capable (Swain & Korenman, 2018), effective (Owens et al., 2013), impactful (Rego et al., 2018), innovative (Yuan et al., 2018), benevolent (Krumrei-Mancuso, 2018; X. Wang et al., 2022), trustworthy (T. Chiu & Hung, 2022), and authentic (Ma et al., 2020; Oc et al., 2020).

Team Outcomes

Leader humility is also linked to several positive team outcomes. For example, research supports what has been called the *social contagion hypothesis* of leader humility (Owens & Hekman, 2016), which posits that "leader behavior can spread via social contagion to followers, producing an emergent state that ultimately affects team performance" (p. 1088). Leader humility particularly contributes to two emergent states—higher team collective humility (Owens & Hekman, 2016; Rego et al., 2017; Ye et al., 2020) and *collective promotion focus* (M. Li et al., 2019; X. Li et al., 2019; Owens & Hekman, 2016), defined as "a collective team focus on progressively striving toward achieving the team's highest potential" (Owens & Hekman, 2016, p. 1089). Leader humility is also linked to more friendly and less conflictual relationships

among team members (C. Chiu et al., 2022), supporting the *social functioning hypothesis* of humility (Worthington et al., 2017), which posits that humility strengthens social bonds (*social bonds hypothesis*; Davis et al., 2013) and helps prevent and repair relational strains (*social oil hypothesis*; McElroy et al., 2014). In addition, leader humility is linked to higher team creativity (X. Wang et al., 2019), innovation (Liu et al., 2017; Mallen et al., 2020), psychological safety (Rego et al., 2021; Swain, 2018), and spirituality (Naseer et al., 2020). Furthermore, it contributes to better team performance (C. Chiu et al., 2016; Owens & Hekman, 2016; Rego et al., 2019), performance capacity (C. Chiu et al., 2022), and *psychological capital* (PsyCap; Rego et al., 2017, 2019), defined as a "team's shared positive appraisal of their circumstances and probability for success under those circumstances based on their combined motivated effort and perseverance" (Peterson & Zhang, 2011, p. 134). In sum, these findings are consistent with the *positive attribution hypothesis of leader humility* (Qin et al., 2020), which asserts that positive attributions of leader humility are what lead to its positive attitudinal and behavioral outcomes.

Psychological Outcomes

In addition, leader humility is linked to positive psychological outcomes, especially as it pertains to followers who work under humble leaders. Consistent with the social contagion (Owens & Hekman, 2016) and positive attribution hypotheses (Qin et al., 2020), leader humility is related to higher follower self-rated humility (Diao et al., 2019; Zhong et al., 2020), PsyCap virtues (hope, optimism, and resilience; Bin et al., 2021), and other virtues (e.g., empathy, gratitude, and authenticity; Naseer et al., 2020; Oc et al., 2020; J. Wang et al., 2017), as well as lower follower burnout (Zhong et al., 2020) and higher follower creativity (J. Wang et al., 2017), proactive behavior (Y. Chen et al., 2018; Zhang & Liu, 2019), and psychological empowerment (Y. Chen et al., 2018; Jeung & Yoon, 2016). Leader humility also contributes to attachment

security (Bharanitharan et al., 2019) and relational closeness (Mao et al., 2017) between followers and leaders, supporting the social functioning hypothesis (Worthington et al., 2017). Among leaders themselves, self-rated humility is related to better mental and spiritual health (Jankowski et al., 2019; Ruffing et al., 2021), supporting the *humility–health hypothesis*, which posits that humility enhances people’s holistic health and well-being (Toussaint & Webb, 2017).

Cultural and Organizational Contexts in Which Leader Humility Has Been Studied

As shown in Table S1, leader humility has been studied in many cultural contexts. Yet, of the 95 distinct studies identified in this systematic review, 51 (53.7%) were conducted in Asia (38 in China; 4 in Taiwan; 2 each in India, Vietnam, Singapore, and South Korea; and 1 in Pakistan), and 34 (35.8%) in North America (all in the U.S.). Only five (5.3%) were conducted in Europe (4 in Portugal and 1 in Spain), one (1.1%) in Australia, and four (4.2%) in multiple continents (2 in Europe and North America; 1 in Europe and Asia; and 1 in all six habitable continents). Almost no studies have been conducted South America, Africa, or the Middle East.

Regarding organizational contexts, 41 (43.2%) of the 95 studies were conducted in varied or multiple sectors (manufacturing, banking/finance, hospitality/tourism, sales/retail, health services/insurance, technology/information-technology). Most other studies were done in a single sector, such as technology/information-technology (k = 10, 10.5%), hospitality/tourism (k = 6, 6.3%), health services/insurance (k = 5, 5.3%), military (k = 4, 4.2%), manufacturing (k = 3, 3.2%), or other sectors (k = 10, 10.5%; banking, telecommunications, pharmaceutical, automobile, religious communities, faith-based higher education, or faith-based humanitarian aid; 1 or 2 each). The remaining studies were conducted with MTurk workers (k = 6, 6.3%) or with business/management students (k = 10, 10.5%) exposed to fictional or simulated scenarios.[2]

[2] We conducted this systematic review and posted the following summary Tables S1 and S2 at https://osf.io/gtu9h/ on July 15, 2022. For a more recent and comprehensive systematic and meta-analysis, see Chandler et al. (2023).

Table S1

*Summary of Features and Findings From Peer-Reviewed Empirical Articles on Leader Humility, 2012 to 2022 (*k *= 65)*

Citation	Sample(s)	Design	Organizational sector(s)	Country	Analytic approach	Key finding(s)
Bharanitharan et al. (2019)	57 leaders, 257 followers	Time-lagged (2 waves, separated by 2 weeks)	Information technology	India	Multilevel modeling	Perceived leader humility enhances followers' sense of attachment security by helping them feel trusted and self-efficacious about voicing their opinions. In turn, this enhanced attachment security contributes to followers paradoxically both advocating for change (engaging in challenging voice behavior) and resisting change (engaging in defensive voice behavior).
Bin et al. (2021)	273 followers	Cross-sectional	Hospitality	U.S.	Hierarchical regression analyses	There was a curvilinear effect between leader humility and employee performance, such that leader humility predicted higher employee performance up to a "tipping" point, beyond which employee performance declined. This curvilinear relationship was attenuated when employees exhibited high *PsyCap* (the psychological resources of hope, efficacy, resilience, and optimism [HERO] that promote positive organizational behavior; Luthans & Youssef-Morgan, 2017; Youssef-Morgan & Luthans, 2015). Cultivation of PsyCap may help employees' performance become less dependent on leaders.

Citation	Sample(s)	Design	Organizational sector(s)	Country	Analytic approach	Key finding(s)
Carnevale et al. (2019)	45 leaders, 233 followers	Time-lagged (3 waves, separated by 3 weeks)	Information technology	China	Multilevel modeling	Leader humility contributes to greater follower identification with the leader, which in turn contributes to higher prosocial behavior among followers. This positive indirect effect only occurs when there is high *leader–member exchange differentiation* (a highly personalized relationship between the leader and follower).
C. Chen et al. (2021)	59 leaders and teams, 286 followers	Time-lagged (2 waves, separated by 2 months)	Information technology	China	Regression-based moderation and conditional-process analyses	For leaders with high *leader–leader exchange* (a high-quality relationship with their own immediate supervisor), perceived leader humility was associated with higher *team job crafting* (“extent to which team members combine efforts to increase structural and social resources at work to cope with challenging job demands…, stimulate initiative…, and promote creativity,” p. 328). However, for leaders with low leader–leader exchange (a poor relationship with their own supervisor), perceived leader humility was associated with *lower* team job crafting.<br>The positive impact of leader humility on team creativity was explained by higher team job crafting but only was present when leaders were high in leader–leader exchange.

Citation	Sample(s)	Design	Organizational sector(s)	Country	Analytic approach	Key finding(s)
L. Chen (2018)	248 female leaders, 534 followers	Cross-sectional	Varied/multiple sectors (8 firms [unspecified sectors])	China	Regression-based moderation analyses	Leaders' self-rated trait narcissism was associated with higher *gender–leader identity integration* ("individual differences in the degree to which one's gender and leader identity are perceived as compatible with or in opposition to each other" (p. 339) but only when perceived leader humility was high. This study supported the counterbalancing effect of leader narcissism and humility (see also Owens et al., 2015).
Y. Chen et al. (2018)	51 leaders, 321 followers, 286 leader–follower dyads	Cross-sectional	Varied/multiple sectors (human resource management, research and development, finance, and sales)	China	Hierarchical linear modeling	Leader humility was associated with higher levels of followers' psychological empowerment and proactive behavior. These relationships were stronger when the follower reported high (vs. low) identification with the leader.
Cheung et al. (2020)	Study 1: 80 leaders, 280 followers<br><br>Study 2: 52 leaders, 282 followers	Study 1: Time-lagged (3 waves, separated by 1 month)<br><br>Study 2: Time-lagged (2 waves, separated by 1 month)	Study 1: 1 large automobile company<br><br>Study 2: 3 large manufacturing companies	Study 1: China<br><br>Study 2: China	Study 1: Multilevel modeling<br><br>Study 2: Multilevel modeling	Study 1 indicated employees' trait mindfulness led to higher creative process engagement, which in turn led to higher supervisor-rated creativity. This indirect effect was replicated in Study 2 but was only present when the leader was perceived as high in humility. Followers' mindfulness only contributed to enhanced follower creativity when they perceived their leader as highly humble.

Citation	Sample(s)	Design	Organizational sector(s)	Country	Analytic approach	Key finding(s)
C. Chiu et al. (2016)	62 leaders and teams, 272 followers	Time-lagged (2 waves, separated by 5 months)	Varied/multiple sectors (5 public relations firms, 1 semi-conductor solution provider company, and 1 large private university)	Taiwan	Regression-based moderation and conditional-process analyses	Leader humility was associated with higher team task performance and higher *shared leadership* (“a group-level phenomenon generated from reciprocal reliance and shared influence among team members so as to achieve team goals,” p. 1705). Leader humility facilitated shared leadership by promoting leadership-claiming and leadership-granting interactions among team members. The impact of perceived leader humility on team shared leadership was even stronger when team members were high in trait-based proactivity. Additionally, when teams were comprised of highly capable people, the effect of shared leadership on team performance was enhanced. Moreover, leader humility led to enhanced shared leadership when team members were high in both trait-based proactivity and performance capability. In sum, teams with humble leaders perform better under shared leadership structures when the followers are both highly proactive and highly competent.

Citation	Sample(s)	Design	Organizational sector(s)	Country	Analytic approach	Key finding(s)
C. Chiu et al. (2021)	83 leaders and teams, 302 followers	Time-lagged (2 waves, separated by 2 weeks)	Varied/multiple sectors (50 telecommunications retail stores and 55 security teams)	China	Regression-based mediation and moderated-mediation analyses, as well as multilevel path modeling	This study found support for the social oil hypothesis of humility at the team level. Team collective humility was associated with lower group experienced incivility (workplace incivility). This effect was especially strong when teams were low in diversity among (a) team members’ humility levels (i.e., when most team members were similar in their individual degree of general humility) and (b) team members’ exposure to incivility (i.e., when individual members experienced similar levels of workplace incivility).
C. Chiu et al. (2022)	Study 1:<br>70 leaders and teams, 298 followers	Study 1:<br>Time-lagged (2 waves, separated by 5 months)	Study 1:<br>Varied/multiple sectors (7 public relations firms, 1 information technology company, and 1 large private university)	Study 1:<br>Taiwan	Study 1:<br>Structural equation modeling	In Study 1, leader humility was associated with lower density of hindrance ties (negative relationships) and higher density of friendship ties (positive relationships) among team members. It also was associated with higher (a) team-helping norms, (b) *team viability* (“collective capacity for long-term growth and future success,” p. 504), and (c) team performance. Leader humility led to less negative (hindering) relationships among team members, which led to stronger team-helping norms (prosocial) and thus to better team viability and team performance.

Citation	Sample(s)	Design	Organizational sector(s)	Country	Analytic approach	Key finding(s)
C. Chiu et al. (2022) (cont’d)	Study 2: 50 leaders and teams, 197 followers	Study 2: Time-lagged (2 waves, separated by 2 weeks)	Study 2: 1 large telecommunications company	Study 2: Taiwan	Study 2: Structural equation modeling	In Study 2, after controlling for transformational leadership, leader humility was unassociated with friendship-tie density but was still associated with lower hindrance-tie density. Moreover, even after controlling for transformational leadership, leader humility led to increased team-helping behavior by decreasing hindrance-tie density, replicating Study 1 findings.
T. Chiu & Hung (2022)	33 leaders, 187 followers	Cross-sectional	Varied/multiple sectors (6 private and 5 public organizations [unspecified sectors])	Taiwan	Regression-based moderation analyses	Leader humility had differential impacts on follower compliance, based on the leader’s levels of perceived trustworthiness and authority. High leader humility was associated with high employee compliance when the leader was perceived as high in both trustworthiness and authority. It was associated with low compliance when the leader was perceived as low in both trustworthiness and authority.
Cojuharenco & Karelaia (2020)	Study 1: 281 leaders who were alumni from a prestigious business school	Study 1: Cross-sectional	Study 1: Varied/multiple sectors (management, business and financial operations, sales, healthcare, and consulting)	Study 1: Multinational (6 continents, 52 countries)	Study 1: Descriptive statistics and logistic regression analyses	In Study 1, most leaders believed asking questions of followers (vs. offering their own conclusions and asking for input) was a better way to seek input, exhibit humility, and evoke trust. They were less likely to ask questions if they thought followers doubted their competence, however.

Citation	Sample(s)	Design	Organizational sector(s)	Country	Analytic approach	Key finding(s)
Cojuharenco & Karelaia (2020) (cont'd)	Study 2: 310 U.S. adults (MTurk)	Study 2: Experimental	Study 2: Aerospace company (fictional scenario)	Study 2: U.S.	Study 2: Analysis of variance (ANOVA)	In Study 2, for leaders whose competence and credentials were doubted beforehand, there was a further competence penalty if they asked questions of followers. For leaders who were already perceived as competent beforehand, there was no such penalty for asking questions.
	Study 3: 329 U.S. adults (MTurk)	Study 3: Experimental	Study 3: Aerospace company (fictional scenario)	Study 3: U.S.	Study 3: ANOVA	In Study 3, leaders who already had a poor reputation for humility received a stronger humility premium/boost for asking questions than leaders who had a good reputation for humility. Yet even leaders with a good prior reputation received a humility boost for asking questions.
	Study 4: 353 U.S. adults (MTurk)	Study 4: Experimental	Study 4: Aerospace company (fictional scenario)	Study 4: U.S.	Study 4: ANOVA	In Study 4, only leaders whose competence was doubted received a competence penalty for asking questions, but this penalty's effects were offset by the humility boost leaders received for asking questions. For all leaders, asking questions led to a humility boost, which buffered against the negative effects of competence penalties on trust and helping intentions. Penalties were higher if leaders asked questions after admitting not knowing something.

Citation	Sample(s)	Design	Organizational sector(s)	Country	Analytic approach	Key finding(s)
Cojuharenco & Karelaia (2020) (cont'd)	Study 5: 502 U.S. adults (MTurk)	Study 5: Experimental	Study 5: Medical device manufacturing company (fictional)	Study 5: U.S.	Study 5: ANOVA	There was no competence penalty of question asking for leaders perceived as nonhumble beforehand, suggesting question asking mainly has a competence penalty for leaders whose followers already doubt their competence—not their humility. For all leaders, there was a humility boost for asking questions, and this boost led to increased trust and helping intentions toward the leader. The competence penalty on trust and helping intentions was only present for leaders whose followers doubted their competence but not their humility.
Cuthbert et al. (2018)	71 religious leaders and their close friends or family members	Longitudinal and quasi-experimental (2 waves, separated by 4 weeks)	Religious communities (Christian churches)	U.S.	Correlational analyses and ANOVAs	There was no evidence that a partner-based humility intervention led to increases in self-rated or observer-rated leader general, relational, spiritual, or intellectual humility. However, there was greater consistency between self- and observer-ratings of leaders' relational humility over time, suggesting leaders may have become more "accurate" in their perceptions of their own relational humility, after participating in a 2-month intervention that focused on cultivating humility.

Citation	Sample(s)	Design	Organizational sector(s)	Country	Analytic approach	Key finding(s)
Diao et al. (2019)	50 leaders, 200 followers	Time-lagged (2 waves, separated by 1 month)	Information technology	China	Regression-based moderation and conditional-process analyses	Leader humility led to increased follower job engagement (work-related passion), which in turn led to higher follower job performance (leader-rated). These direct and indirect effects were even stronger when followers' self-rated humility was high. In addition, followers' self-rated humility was related to their perceptions of their leader's humility, their job engagement (harmonious passion), and their leader-rated job performance.
Hu et al. (2018)	72 leaders and teams, 354 followers	Time-lagged (3 waves, separated by 3 months)	11 information technology and technology companies	China	Path analyses	Leader humility was only linked to *higher* team information sharing when team power distance ("extent to which members see the distance between leaders and themselves as legitimate," p. 314) was low and linked to *lower* team psychological safety when team power distance was high. Leader humility only had a positive effect on team creativity via increased information sharing when team power distance was low and only had a negative effect on team creativity via decreased team psychological safety when team power distance was high. Leader humility may benefit teams with low power distance more than teams with high power distance.

Citation	Sample(s)	Design	Organizational sector(s)	Country	Analytic approach	Key finding(s)
Jankowski et al. (2019)	258 religious leaders	Cross-sectional	Religious communities	U.S.	Regression-based mediation and moderated-mediation (conditional process) analyses	Leader humility among religious leaders was associated with lower insecure attachment to God, higher positive mental health, lower negative mental health, and lower trait narcissism. However, leaders’ religion-specific intellectual humility had the opposite effects.
Jeung & Yoon (2016)	294 followers	Cross-sectional	Varied/multiple sectors (a Korean business conglomerate that mainly consists of information technology and sales/marketing companies)	South Korea	Regression-based moderation analyses	Leader humility predicted higher employee psychological empowerment. This effect was strongest when high leader humility was combined with high *power distance orientation* (“extent to which an individual is oriented to power distance as a cultural value,” p. 1127) and *hierarchical distance* (“the difference in hierarchy level between followers and their direct supervisors,” p. 1128).
Jeung & Yoon (2018)	306 followers	Cross-sectional	Varied/multiple sectors (a Korean business conglomerate that mainly consists of information technology and sales/marketing companies)	South Korea	Regression-based moderation and conditional-process analyses	Leader humility was associated with higher psychological empowerment, which in turn was associated with higher employee *prosocial voice* (“discretionary upward communication behavior that emphasizes expression of a constructive challenge,” p. 42). This indirect effect was strongest when followers were high in power distance orientation.

Citation	Sample(s)	Design	Organizational sector(s)	Country	Analytic approach	Key finding(s)
Krumrei-Mancuso (2018)	29 college student leaders, 29 followers	Longitudinal (2 waves, separated by 6 weeks)	Faith-based higher education (1 Christian college)	U.S.	Hierarchical regression analyses	Student leaders’ increases in relational humility and intellectual humility were predictive of their followers’ ratings of the leader’s servant leadership behaviors, kindness toward followers, and trait empathy.
M. Li et al. (2019)	89 teams, 305 followers	Time-lagged (3 waves, separated by 1 month)	Information technology (8 companies in the internet service industry)	China	Regression-based moderation and conditional-process analyses	Leader humility was associated with higher shared mental models (“tacit agreements that exist among team members [and reflect] teams’ shared cognition,” p. 654), which in turn were associated with higher team learning. The indirect effect of leader humility on team learning via shared mental models was strongest when teams had a high *collective promotion focus* (“team focus on giving efforts related to approaching opportunities to achieve the team’s highest potential, motivating team members to pay attention to what they want to achieve rather than what could go wrong,” p. 658).
X. Li et al. (2019)	54 leaders, 230 followers	Cross-sectional	Varied/multiple sectors (manufacturing, real estate, information technology, and research and design)	China	Hierarchical linear modeling	Leader humility was associated with higher employee *voice* (“promotive behavior that emphasizes the expression of constructive challenge,” Van Dyne & LePine, 1998, p. 109) via *voice–role conception* (how much they see voice as job-relevant).

Citation	Sample(s)	Design	Organizational sector(s)	Country	Analytic approach	Key finding(s)
X. Li et al. (2019) (cont’d)						The indirect effect of leader humility on voice via voice–role conception was strongest when teams had a high collective promotion focus.
X. Li et al. (2021)	41 leaders, 237 followers	Cross-sectional	15 technology firms	China	Regression-based mediation analysis	Follower-rated leader humility was associated with higher follower job engagement (self-rated), which in turn was associated with higher leader-rated creative performance. Leader humility was associated with follower creative performance both directly and indirectly (via job engagement).
Lie et al. (2016)	50 graduate student leaders	Cross-sectional (qualitative)	1 student-run healthcare clinic (providing medical, physician assistant, occupational therapy, and pharmaceutical services in an urban setting)	U.S.	Constant comparison analysis	When asked what they learned through their process of providing healthcare services in an urban student-run clinic, “humility and responsible leadership” were two themes that were uniquely mentioned by medical school student leaders. These themes were not mentioned by student leaders from the other disciplines.
Lin et al. (2019)	48 leaders, 152 followers	Time-lagged (2 waves, separated by 2 weeks)	1 large pharmaceutical company	China	Regression-based mediation and conditional-process analyses	Leader humility had a positive indirect effect on followers’ voice via enhancing followers’ personal sense of power, but this indirect effect was only present among employees with low power distance orientation (low cultural valuing of the power distance between leaders and followers).

Citation	Sample(s)	Design	Organizational sector(s)	Country	Analytic approach	Key finding(s)
Liu et al. (2017)	90 leaders and teams, 307 followers	Cross-sectional	Varied/multiple sectors (research and development, production, and sales)	China	Regression-based mediation and conditional-process analyses	Leader humility was related to higher *team voice climate* ("a shared belief that speaking up is safe and efficient," p. 2), team innovation, and task interdependence. Leader humility also had an indirect effect on team innovation via enhancing team voice climate, but this effect was only present when task interdependence was low.
Luu (2020)	82 leaders and teams, 529 followers	Time-lagged (2 waves, separated by 2 months)	Retail shops of 1 large retail company in the information technology sector	Vietnam	Multilevel structural equation modeling	Leader humility was linked to followers' and leaders' *adaptive selling behavior* ("behavior to adapt sales presentations to meet specific demands of specific types of customers," p. 1292). Leader humility was also linked to higher levels of employees' (a) *customer knowledge* (knowledge "regarding customer attributes and strategies for addressing varying customer expectations and needs," p. 1293), (b) *adaptive self-efficacy* ("belief that he or she is competent to adapt to new work aspects or master new task demands," p. 1293), and (c) *customer-oriented harmonious passion* ("a positive affective response leading to the internalization of customer-oriented value and acceptance of customer service as vital, p. 1293).

Citation	Sample(s)	Design	Organizational sector(s)	Country	Analytic approach	Key finding(s)
Luu (2020) (cont’d)						All three of these factors mediated the effect of leader humility on employees’ adaptive selling behavior. The combination of high leader humility and leader adaptive selling behavior was also associated with higher adaptive self-efficacy and customer-oriented harmonious passion.
Ma et al. (2020)	Study 1:<br>88 leaders,<br>449 followers<br><br>Study 2:<br>50 leaders,<br>185 followers	Study 1:<br>Time-lagged (3 waves, separated by 2 weeks)<br><br>Study 2:<br>Time-lagged (3 waves, separated by 2 weeks)	Study 1:<br>1 large pharmaceutical company<br><br>Study 2:<br>1 large manufacturing company	Study 1:<br>China<br><br>Study 2:<br>China	Study 1:<br>Multilevel modeling<br><br>Study 2:<br>Multilevel modeling	In Study 1, leader humility led to increased employee relational energy, which led to increased constructive voice behavior.<br><br>In Study 2, the indirect effect of leader humility on employee constructive voice behavior via enhanced relational energy was replicated. However, the indirect effect was only present when followers perceived their leader’s sincerity (authenticity) as high. In other words, employees were only energized and motivated toward proactive engagement by humble leaders they perceived as sincere.
Mallen et al. (2020)	568 leaders	Cross-sectional	Varied/multiple sectors (284 companies [unspecified sectors])	Spain	Structural equation modeling	Leaders’ self-reported humility was associated with higher ratings of their own altruism (prosocial behaviors) and their firm’s innovativeness. Leader humility had an indirect effect on firm innovativeness via leader altruism.

Citation	Sample(s)	Design	Organizational sector(s)	Country	Analytic approach	Key finding(s)
Mao et al. (2017)	295 leaders, 295 followers	Time-lagged (2 waves, separated by 7 weeks)	Varied/multiple sectors (13 companies [unspecified sectors])	China	Regression-based mediation and conditional-process analyses	Leader humility was associated with higher relational closeness between followers and leaders. It also was associated with increased employee voice and helping behaviors, and relational closeness explained these links.
Mao et al. (2019)	57 leaders, 256 followers	Time-lagged (2 waves, separated by 6 weeks)	Varied/multiple sectors (manufacturing, real estate, and trading)	China	Structural equation modeling	Leader humility was associated with higher employee self-expansion, which in turn was associated with enhanced follower self-efficacy and leader-rated job performance. This indirect effect of leader humility on job performance via self-expansion and self-efficacy was only present when followers and leaders were similar in age or gender.
Naseer et al. (2020)	286 followers and their peer colleagues	Time-lagged (3 waves, separated by 1 month)	Varied/multiple sectors (9 companies in the telecommunications, education, and industrial sectors)	Pakistan	Structural equation modeling	Leader humility was associated with higher workplace spirituality as well as higher follower empathy, gratitude, and peer-reported ethical behavior. Workplace spirituality mediated (causally explained) the effect of leader humility on all three of these follower outcomes. That is, leader humility led to higher workplace spirituality, which in turn led to higher follower empathy, gratitude, and ethical behavior.

Citation	Sample(s)	Design	Organizational sector(s)	Country	Analytic approach	Key finding(s)
Nguyen et al. (2020)	252 followers	Time-lagged (2 waves, separated by 1 month)	Varied/multiple sectors (mostly "health care and social assistance, scientific and technical services, education and training, and administrative support services," p. 5)	Australia	Structural equation modeling	Leader humility was linked to followers' higher affective trust in their supervisor, job engagement, organizational citizenship behavior toward coworkers, and knowledge sharing intention. Leader humility led to affective trust, then job engagement, then organizational citizenship behavior, and finally knowledge sharing intention. Leader humility also led to job engagement, then organizational citizenship, and last knowledge sharing intention.
Oc et al. (2015)	Study 1:<br>20 leaders, 5 followers<br><br>Study 2:<br>307 leaders	Study 1:<br>Cross-sectional (qualitative)<br><br>Study 2:<br>Cross-sectional (qualitative)	Study 1:<br>Varied/multiple sectors (government, service, manufacturing, financial, education, and transportation)<br><br>Study 2:<br>Varied/multiple sectors (service, financial, government, manufacturing, human services, transportation, and other industries)	Study 1:<br>Singapore<br><br>Study 2:<br>Singapore	Study 1:<br>Inductive (conventional) content analysis<br><br>Study 2:<br>Inductive (conventional) content analysis	In Study 1 and Study 2, nine dimensions of humble leadership in a Singaporean context emerged. Four dimensions resonated with extant Western theories of leader humility: "having an accurate view of self, recognizing follower strengths and achievements, modeling teachability and being correctable, and showing mutual respect and fairness" (p. 76). Five others were culturally distinct: "leading by example, showing modesty, working together for the collective good, empathy and approachability, and mentoring and coaching" (p. 76). Leader humility may have both culturally unique dimensions and cross-culturally universal dimensions.

Citation	Sample(s)	Design	Organizational sector(s)	Country	Analytic approach	Key finding(s)
Oc et al. (2020)	Study 1: 258 leaders, 258 followers	Study 1: Time-lagged (2 waves, separated by 1 month)	Study 1: Varied/multiple sectors (technology, manufacturing, service, finance, pharmaceutical, and other industries)	Study 1: India	Study 1: Structural equation modeling	In Study 1, there was an indirect effect of leader humility on followers’ felt authenticity via decreased sense of vulnerability, but this effect was only present when followers perceived their leader’s humility to be authentic.
	Study 2: 325 followers (ClearVoice Research)	Study 2: Experimental	Study 2: Varied/multiple sectors (unspecified)	Study 2: U.S.	Study 2: Hierarchical regression analyses	In Study 2, leader humility was causally related to followers’ decreased sense of vulnerability.
	Study 3: 103 followers (MTurk)	Study 3: Experimental	Study 3: Varied/multiple sectors (unspecified)	Study 3: U.S.	Study 3: Hierarchical regression analyses	In Study 3, feelings of perceived vulnerability to one’s supervisor were causally related to decreased felt authenticity.
	Study 4: 207 followers (ClearVoice Research)	Study 4: Experimental	Study 4: Varied/multiple sectors (service, finance, manufacturing, government, transportation, human services, and other industries)	Study 4: U.S.	Study 4: Structural equation modeling	Study 4 replicated the findings of Study 1 but with evidence that more definitively supported causal inference. Leader humility led directly to increased felt authenticity. It also led indirectly to increased felt authenticity by leading to decreased feelings of vulnerability. However, this indirect effect was only present when followers perceived the leader’s humility to be authentic (see also Ma et al., 2020; Owens & Hekman, 2012).

Citation	Sample(s)	Design	Organizational sector(s)	Country	Analytic approach	Key finding(s)
Ou et al. (2017)	43 leaders’ teams (top management teams), 313 leaders (top executives who were members of the top management teams), 502 followers (middle managers)	Time-lagged (3 waves: baseline, 2-weeks postbaseline, and 1-year postbaseline)	Varied/multiple sectors (companies in the manufacturing, service, and trading sectors)	China	Hierarchical linear modeling	The positive impact of leader humility on follower job satisfaction was only present when leaders’ management team was characterized by a low degree of *faultlines* (“hypothetical dividing lines that split a [team] into homogeneous subgroups based on members’ alignment along one or more attributes,” p. 1916). That is, when management teams were characterized by low diversity (in terms of “age, gender, education level, education specialization, company tenure, and team tenure,” p. 1920), leader humility had a positive impact on follower job satisfaction. This effect was not present when leaders’ management team was characterized by high diversity (faultlines). Similarly, there was an indirect effect of leader humility on followers’ decreased voluntary turnover via followers’ increased job satisfaction, but this indirect effect was again only present when leaders’ teams were characterized by low diversity (faultlines). These direct and indirect effects emerged both for social-categorical and task-related faultlines (surface- and deep-level team diversity; Davis et al., 2021).

Citation	Sample(s)	Design	Organizational sector(s)	Country	Analytic approach	Key finding(s)
Owens & Hekman (2012)	55 leaders	Cross-sectional (qualitative)	Varied/multiple sectors (mortgage banking, information technology, hospitals and healthcare, financial services, and retail/sales, religious, manufacturing, industrial, and military)	U.S.	Thematic analysis	This study involved developing a theoretical model of the behaviors, contingencies, mechanisms, and outcomes of humble leadership. Results indicated three key humble leader behaviors: (a) acknowledgement of personal limitations, faults, and mistakes; (b) spotlighting of followers’ strengths and contributions; and (c) modeling of teachability and growth-mindset. There are contingencies to these behaviors in that the leader needs to be perceived as competent and authentic, and these behaviors are *more* effective and appropriate in organizations that have a learning-/growth-oriented culture and are *less* effective and appropriate in organizations that have a hierarchical culture or are facing extreme threat or time pressure. The main outcomes produced by humble leader behaviors are increased (a) relational trust, loyalty, and satisfaction; (b) psychological freedom; (c) job engagement; (d) preference for small, continuous organizational change; and (e) *fluid organizing* (“ease and swiftness in transitioning to different ways of functioning,” p. 802).

Citation	Sample(s)	Design	Organizational sector(s)	Country	Analytic approach	Key finding(s)
Owens & Hekman (2012) (cont’d)						These outcomes emerge because the leader’s humble behaviors legitimize and validate followers’ (a) developmental journey (efforts to grow) and (b) feelings of uncertainty (due to the dynamics of organizational change/fluidity).
Owens & Hekman (2016)	Study 1:<br>89 undergraduate business students in 31 teams (16 in humble leader condition; 15 in nonhumble leader condition)<br><br>Study 2:<br>192 undergraduate business students in 53 teams	Study 1:<br>Experimental<br><br>Study 2:<br>Longitudinal (2 waves, separated by 6 weeks)	Study 1:<br>Simulated team task in a laboratory (for a fictional hardware chain company)<br><br>Study 2:<br>Computer-simulated team task to compete for market share and stock value in the automobile manufacturing market	Study 1:<br>U.S.<br><br>Study 2:<br>U.S.	Study 1:<br>Structural equation modeling and regression-based path analyses<br><br>Study 2:<br>Structural equation modeling and regression-based path analyses	In Study 1, leader humility (a team input) caused increased *collective humility* (“a group tendency toward owning limitations and mistakes, appreciating group members’ strengths, and being teachable,” p. 1089; an interpersonal team process), which in turn led to increased *collective promotion focus* (“a collective team focus on progressively striving toward achieving the team’s highest potential,” p. 1089; a shared team motivation).<br><br>In Study 2, collective humility led to improved team performance by leading to increased collective promotion focus, even when controlling for team cohesion and psychological safety. In other words, a team’s collective promotion focus is the mediator (causal mechanism) that explains the impact of team humility on improved team performance.

Citation	Sample(s)	Design	Organizational sector(s)	Country	Analytic approach	Key finding(s)
Owens & Hekman (2016) (cont'd)	Study 3:<br>326 employees<br>(77 work teams)	Study 3:<br>Longitudinal (2 waves, separated by 1 month)	Study 3:<br>1 large multinational health services organization	Study 3:<br>Multinational (U.S., Puerto Rico, and U.K.)	Study 3:<br>Structural equation modeling and regression-based path analyses	In Study 3 (a longitudinal field study), leader humility influenced team performance by leading to higher collective humility and then higher collective promotion focus. This serial mediation effect supported the *social contagion hypothesis* of leader humility, which posits that "leader behavior can spread via social contagion to followers, producing an emergent state that ultimately affects team performance" (p. 1088).
Owens et al. (2013)	Study 1:<br>524 undergraduate business students (Studies 1a to 1c), 774 employees (Studies 1d and 1e)<br><br>Study 2:<br>144 undergraduate business students	Study 1:<br>Cross-sectional<br><br>Study 2:<br>Longitudinal (3 waves, separated by 5 weeks)	Study 1:<br>Varied/multiple sectors (1a and 1b: someone students knew well; 1c: students' fellow project team member; 1d and 1e: employees' immediate supervisor)<br><br>Study 2:<br>Undergraduate management class	Study 1:<br>U.S.<br><br>Study 2:<br>U.S.	Study 1:<br>Factor analyses and nomological network analysis<br><br>Study 2:<br>Hierarchical regression analyses	Study 1 involved the development and initial validation of the Expressed Humility Scale, an observer-report measure of *expressed humility* ("an interpersonal characteristic that emerges in social contexts [and] connotes (a) a manifested willingness to view oneself accurately, (b) a displayed appreciation of others' strengths and contributions, and (c) teachability," p. 1518).<br><br>In Study 2, other-rated expressed humility predicted other-rated (a) team contribution and (b) individual performance. It had an especially strong predictive effects among team members with lower general mental ability.

Citation	Sample(s)	Design	Organizational sector(s)	Country	Analytic approach	Key finding(s)
Owens et al. (2013) (cont’d)	Study 3: 704 followers	Study 3: Time-lagged (3 waves: baseline, 1-month postbaseline, and 5-months postbaseline)	Study 3: 1 large health services organization	Study 3: U.S.	Study 3: Hierarchical regression analyses and hierarchical linear modeling	In Study 3, leader humility was linked to higher job engagement, job satisfaction, and team *learning goal orientation* (“an adaptive approach to task situations associated with the motivation to understand and master the task rather than to display or prove competence,” p. 1521; growth-mindset). It also was associated with lower voluntary turnover. Leader humility had an indirect impact on followers’ job engagement by improving team learning goal orientation (growth mindset). It also led to decreased follower voluntary turnover by enhancing their job satisfaction.
Owens et al. (2015)	78 leaders, 138 teams, 230 followers	Time-lagged (3 waves, separated by 1 month)	1 large health insurance organization	Multinational (U.S., Puerto Rico, and Western European countries)	Hierarchical regression analyses and hierarchical linear modeling	Leaders who exhibited high humility were perceived as more effective, and leaders who exhibited both high narcissism and high humility were perceived as more effective also. Leaders who exhibited higher narcissism and humility had followers who reported better job engagement and exhibited better objective and leader-rated job performance. Narcissistic leaders may enhance their employees’ job engagement and performance if they temper or counterbalance their narcissism with humble leader behaviors.

Citation	Sample(s)	Design	Organizational sector(s)	Country	Analytic approach	Key finding(s)
Owens et al. (2019)	Study 1:<br>64 leaders,<br>295 followers<br><br>Study 2:<br>250 employees (recruited through Qualtrics)	Study 1:<br>Time-lagged (2 waves, separated by 7 weeks)<br><br>Study 2:<br>Time-lagged (3 waves, separated by 2 weeks)	Study 1:<br>Varied/multiple sectors (manufacturing, real estate, and technology organizations)<br><br>Study 2:<br>Varied/multiple sectors (unspecified)	Study 1:<br>China<br><br>Study 2:<br>U.S.	Study 1:<br>Hierarchical linear modeling and regression-based mediation and conditional-process analyses<br><br>Study 2:<br>Hierarchical linear modeling and regression-based mediation and conditional-process analyses	Both Study 1 and Study 2 focused on *leader moral humility* ("a leader's propensity to show humility in the context of dealing with a moral issue as reflected by (a) a willingness to view his or her moral competence accurately, (b) an appreciation of others' moral strengths and behaviors, and (c) moral teachability," p. 147) and followers' *moral self-efficacy* ("belief in his/her capabilities to organize and mobilize the motivation, cognitive resources, means, and courses of action needed to attain moral performance," Hannah et al., 2011, p. 675). In both Study 1 (an Eastern context) and Study 2, (a Western context), leader moral humility predicted followers' moral self-efficacy, which in turn predicted higher follower prosocial behavior and lower follower unethical behavior. These indirect effects were stronger among followers with an implicit growth mindset toward morality (an "incremental theory" that morality is malleable), relative to those with an implicit fixed mindset (an "entity theory" that morality is innate and stable).

Citation	Sample(s)	Design	Organizational sector(s)	Country	Analytic approach	Key finding(s)
Peng et al. (2020)	128 leaders and teams, 380 followers	Time-lagged (2 waves, separated by 1 month)	Varied/multiple sectors (unspecified)	China	Structural equation modeling	This study examined a "trickle-down" theory of leader humility. *Skip-level leader humility* (humility in leaders at least two organizational-hierarchy levels above the focal employee) was associated with higher team-leader humility, which was associated with better team performance. Skip-level leader humility strengthened the positive impact of team-leader humility on team performance. Moreover, in organizations with high authority centralization (hierarchically oriented companies), the impact of skip-level leader humility on team-leader humility was strongest and so was the indirect effect of skip-level leader humility on team performance via team-leader humility.
Qian et al. (2018)	57 leaders, 248 workers	Cross-sectional	Hospitality (a hotel group)	China	Regression-based mediation and moderated-mediation (conditional process) analyses	Leader humility was directly associated with higher levels of follower feedback-seeking behavior. It also was associated with higher feedback-seeking behavior indirectly, via lower follower perceptions of *image costs* ("potential costs for asking for feedback," p. 2). This indirect effect was strongest for followers with low power distance orientation.

Citation	Sample(s)	Design	Organizational sector(s)	Country	Analytic approach	Key finding(s)
Qin et al. (2020)	Study 1: 275 followers (recruited via WeChat)<br><br>Study 2: 161 followers	Study 1: Time-lagged (3 waves, separated by 2 weeks)<br><br>Study 2: Experimental	Study 1: Varied/multiple sectors (manufacturing, service, finance, technology, and other)<br><br>Study 2: Varied/multiple sectors (manufacturing, real estate, technology, service, and others)	Study 1: China<br><br>Study 2: China	Study 1: Regression-based mediation and conditional-process analyses<br><br>Study 2: Regression-based mediation and conditional-process analyses	In both Study 1 and 2, perceived leader humility led to higher follower workplace deviance via higher follower psychological entitlement, but this indirect effect was only present when followers made self-serving attributions for the leader's humility (e.g., "I have more knowledge/skills than my leader," "My leader needs my advice and can learn from me,").
Qin et al. (2021)	54 leaders, 433 followers	Time-lagged (2 waves, separated by 1 month)	Banking	China	Cross-level polynomial regression analyses and response surface modeling	Congruence between leader and follower humility levels was associated with lower negative affect toward one's supervisor. This effect was stronger in leader–follower dyads when both people were high in humility, relative to dyads when both people were low in humility. When followers' humility was lower than their leader's, followers experienced more negative affect toward their supervisor, which led followers to exhibit higher organizational deviance and lower organizational citizenship behaviors.
Qiuyun et al. (2020)	186 followers	Time-lagged (2 waves, separated by 2 weeks)	1 information technology company	China	Regression-based mediation and conditional-process analyses	Leader humility predicted higher follower sense of power, which predicted followers' lower organizationally deviant behavior. This indirect effect was only present among followers with low organizational identification.

Citation	Sample(s)	Design	Organizational sector(s)	Country	Analytic approach	Key finding(s)
Rego et al. (2017)	82 leaders and teams, 332 followers	Cross-sectional	Varied/multiple sectors (retail/sales, financial/accounting, government, and healthcare)	Portugal	Regression-based mediation and conditional-process analyses	There was an indirect effect of leader humility on team PsyCap via collective humility (team humility), supporting the social contagion hypothesis of leader humility (Owens & Hekman, 2016). This effect was stronger on teams in which there was greater consensus that the leader expressed humility consistently across team members.
Rego et al. (2018)	96 leaders, 307 followers, 656 of leaders’ peers, 96 of leaders’ supervisors (skip-level leaders)	Cross-sectional	Varied/multiple sectors (energy, consulting, software, telecommunications, electronics, pharmaceutical, healthcare, banking/finance, retail/sales, and information technology)	Portugal	Path analysis	Leaders’ self-reported humility linked to ratings of their humility by their peers and supervisors (but not by their followers). The leader’s perceived impact on team effectiveness was related both to follower-rated leader humility and leaders’ self-rated humility. In addition, follower-rated leader humility was associated with follower-rated leader trustworthiness. There was an indirect effect of peer-rated leader humility on perceived leader impact on team effectiveness via peer-rated *balanced processing* (“the degree to which a leader objectively analyzes all relevant data before making decisions [including views] that challenge deeply held personal beliefs [and] information that contradicts an initial viewpoint,” p. 207).

Citation	Sample(s)	Design	Organizational sector(s)	Country	Analytic approach	Key finding(s)
Rego et al. (2018) (cont'd)						This indirect effect did not emerge for follower-rated leader humility or for leader's self-rated humility. Overall, there was low agreement between self- and other-reported measures of leader humility.
Rego et al. (2019)	Study 1: 97 teams, 326 followers (business school students)	Study 1: Experimental	Study 1: Unspecified (fictional scenario given to student-team workgroups)	Study 1: Multinational (Portugal and Singapore)	Study 1: Independent-samples *t*-test	In Study 1, leader humility caused increased expectations of team psychological capital (*PsyCap*: sense of hope, efficacy, resilience, and optimism [HERO within]).
	Study 2: 70 teams, 282 followers	Study 2: Cross-sectional	Study 2: Varied/multiple sectors (healthcare, consulting, retail/sales, hospitality, insurance, automobile, banking/finance, telecommunications)	Study 2: Portugal	Study 2: Regression-based mediation analyses	In Study 2, follower-rated leader humility had an indirect effect on leader-rated team performance via follower-rated team PsyCap. That is, leader humility was associated with better team performance by its enhancing of team PsyCap.
	Study 3: 53 leaders and teams, 203 followers	Study 3: Time-lagged (3 waves, separated by 2 weeks)	Study 3: Varied/multiple sectors (sales, research and development, and human resource)	Study 3: China	Study 3: Regression-based mediation analyses	In Study 3, follower-rated leader humility had an indirect effect on leader-rated team performance via follower-rated team PsyCap and task-allocation effectiveness.
Rego et al. (2021)	85 teams, 354 followers	Cross-sectional	Varied/multiple sectors (35 organizations from unspecified but numerous sectors)	Portugal	Cross-level polynomial regression analyses and response surface modeling	Leader humility predicted higher team psychological safety, and this effect was strongest when there was high team consensus the leader expressed humility consistently across members. Results also suggested leaders may overestimate their humility, relative to followers' perceptions.

Citation	Sample(s)	Design	Organizational sector(s)	Country	Analytic approach	Key finding(s)
Ruffing et al. (2021)	111 religious leaders (seminary students)	Cross-sectional	Faith-based higher education (seminary)	U.S.	Correlational and hierarchical regression analyses	Among religious leaders, leader humility and humility-cultivating practices predicted leaders’ positive mental health, even after accounting for the influence of relational spirituality (attachment to God, differentiation of self, and quest religious orientation).
Shannonhouse et al. (2019)	13 leaders in faith-based humanitarian aid (peer-nominated exemplars of humble leadership)	Cross-sectional (qualitative)	Faith-based humanitarian aid	U.S.	Consensual qualitative research	In a humanitarian aid context, the main identified *humble leader characteristics* were (a) intrinsic religiousness (they are faith-focused), (b) virtuous character (they are encouraging, empowering, intentional, generous, grateful, courageous, respectful, responsible, patient, wise, honest, and resilient), (c) self-awareness (of their strengths and shortcomings), (d) social/emotional intelligence, (e) openness and teachability, (f) broad-mindedness and mission-focus, and (g) social connectedness and interdependence. The main identified *behaviors of humble leaders* were (a) being responsive and collaborative, (b) listening and communicating well, (c) being present and available, (d) acknowledging and handling mistakes, and (e) being other-focused servant leaders.

Citation	Sample(s)	Design	Organizational sector(s)	Country	Analytic approach	Key finding(s)
Shannonhouse et al. (2019) (cont’d)						Leader humility had *benefits* for leaders (work is more meaningful; helps develop other virtues) and their associates (empowerment and engagement of workers and local partners), organizations (lower burnout and politics; better effectiveness, productivity, cohesion), and humanitarian aid beneficiaries (feel valued, understood, sacred). However, some *barriers to leader humility* were identified, including barriers at the individual level (leader pride/arrogance, feeling overburdened/overstressed, and having problematic employees) and at the systemic level (humanitarian aid is ongoing and demanding/stressful and involves large organizations and complex systems; challenges related to marketing and donor fundraising).
Swain (2018)	Study 1: 320 followers (MTurk)<br><br>Study 2: 314 followers (MTurk)	Study 1: Experimental (fictional scenario presented online)<br><br>Study 2: Experimental (fictional scenario presented online)	Study 1: Unspecified-sector organization (fictional scenario before joining a virtual team)<br><br>Study 2: Unspecified-sector organization (fictional scenario imagining being on a virtual team)	Study 1: U.S.<br><br>Study 2: U.S.	Study 1: Regression-based mediation analyses<br><br>Study 2: Regression-based mediation analyses	In Study 1 and Study 2, leader humility increased psychological safety and liking of the leader, which enhanced followers’ intention to engage in information flow behavior within their team.

Citation	Sample(s)	Design	Organizational sector(s)	Country	Analytic approach	Key finding(s)
Swain (2018) (cont’d)	Study 3: 147 followers (community members)	Study 3: Experimental (online chat session in a lab)	Study 3: Virtual teams (fictional scenario)	Study 3: U.S.	Study 3: Multivariate analysis of variance (MANOVA) and logistic regression	In Study 3, participants worked on a virtual team (online chat session) that was tasked with correctly solving who was at fault in a fictitious traffic accident. As in Study 1 and 2, leader humility increased psychological safety and liking of the leader. However, it did not predict actual (objective) information flow behavior or team performance in Study 3.
Swain & Korenman (2018)	Study 1: 143 Army officers<br><br>Study 2: 352 Army officers<br><br>Study 3: 152 Army officers<br><br>Study 4: 183 Army officers	Study 1: Cross-sectional<br><br>Study 2: Experimental<br><br>Study 3: Experimental<br><br>Study 4: Experimental	Study 1: Military<br><br>Study 2: Military<br><br>Study 3: Military<br><br>Study 4: Military	Study 1: U.S.<br><br>Study 2: U.S.<br><br>Study 3: U.S.<br><br>Study 4: U.S.	Study 1: Hierarchical regression analysis<br><br>Study 2: Two-way ANOVA<br><br>Study 3: Two-way ANOVA<br><br>Study 4: Two-way ANOVA	In Study 1, perceived leader humility predicted perceived leadership potential, and this effect was similar for rated men and women officers.<br><br>In Study 2, leader humility again predicted perceived leadership potential, with no gender effect.<br><br>In Study 3, leader humility and positively valenced leader narcissism had positive effects on perceived leadership potential.<br><br>Leader humility again predicted higher perceived leadership potential, but female leaders who expressed low humility were rated as having more leadership potential, whereas male leaders who expressed high humility were rated as having more potential.

Citation	Sample(s)	Design	Organizational sector(s)	Country	Analytic approach	Key finding(s)
Tuan et al. (2021)	136 leaders, 38 teams, 894 followers	Time-lagged (2 waves, separated by 2 months)	Tourism	Vietnam	Multilevel structural equation modeling	Leader humility had a direct effect on service-oriented organizational citizenship behaviors (exceptional service) and an indirect effect via enhanced employee job crafting (seeking challenges and seeking social and structural resources).
Walters & Diab (2016)	140 followers (MTurk)	Cross-sectional	Varied/multiple sectors (Unspecified)	U.S.	Regression-based mediation analysis	Leader humility had a direct effect on employee job engagement and an indirect effect via enhancing followers’ sense of psychological safety at their organization.
J. Wang et al. (2017)	129 leaders and teams, 451 followers	Cross-sectional	Healthcare (hospitals providing disaster relief following the 2008 Wenchuan earthquake)	China	Hierarchical linear modeling	Leader humility in a disaster context was linked to higher levels of followers’ self-reported *perspective taking* (“a cognitive process in which individuals adopt others’ viewpoints in an attempt to understand their preferences, values, and needs,” p. 6) and capacity for *cognitive reappraisal* (“cognitively transforming the situation so as to alter its emotional impact,” p. 14). It also was related to higher leader-reported employee creativity. Leader humility had an indirect effect on disaster workers’ creativity via enhancing their perspective taking, but this effect was only present among workers who were high (vs. low) in cognitive reappraisal capacity.

Citation	Sample(s)	Design	Organizational sector(s)	Country	Analytic approach	Key finding(s)
L. Wang et al. (2018)	Study 1:<br>211 leaders,<br>211 followers<br><br>Study 2:<br>201 leaders, 85 teams,<br>201 followers	Study 1:<br>Time-lagged (2 waves, separated by 3 weeks)<br><br>Study 2:<br>Time-lagged (3 waves, separated by 3 weeks)	Study 1:<br>Varied/multiple sectors (service, consulting, banking, and technology)<br><br>Study 2:<br>Engineers and technicians at a company that sets up and maintains monitoring systems for government and business institutions	Study 1:<br>China<br><br>Study 2:<br>China	Study 1:<br>Multilevel regression analyses and path analyses<br><br>Study 2:<br>Multilevel structural equation modeling	In Study 1, leader humility had an indirect effect on employee task performance via enhancing followers' relational energy and lessening their emotional exhaustion, but this effect was only present when leaders' perceived power was high. Study 1 findings were replicated in Study 2, which also examined antecedents of leader humility. Leader humility was predicted by leaders reporting higher *growth mindset* (incremental theory of self; "see their abilities, attributes, and character as malleable," 1024) and *relational identity* (defining oneself based on relational connections; Johnson et al., 2006).
X. Wang et al. (2019)	104 leaders and teams, 341 followers	Time-lagged (2 waves, separated by 1 month)	Varied/multiple sectors (sales, retail, service, and hotel/hospitality companies)	China	Multilevel structural equation modeling	Leader humility had a direct effect on team creativity and an indirect effect via teams' enhanced sense of creative efficacy. This indirect effect was stronger when leaders were high in conscientiousness. Team creativity also had a direct effect on team performance, and this effect was stronger when teams were high in future orientation. The serial mediation was strongest when leaders were high in conscientiousness and teams high in future orientation.

Citation	Sample(s)	Design	Organizational sector(s)	Country	Analytic approach	Key finding(s)
X. Wang et al. (2022)	Study 1:<br>330 followers<br><br>Study 2:<br>233 followers	Study 1:<br>Time-lagged (2 waves, separated by 6 weeks)<br><br>Study 2:<br>Time-lagged (3 waves, separated by 6 weeks)	Study 1:<br>Hospitality (hotel employees)<br><br>Study 2:<br>Hospitality (hotel employees)	Study 1:<br>China<br><br>Study 2:<br>China	Study 1:<br>Path analyses<br><br>Study 2:<br>Path analyses	In Study 1 and 2, leader humility had a direct effect on employees' *affective commitment* ("emotional attachment to, identification with, and involvement in the organization," Meyer et al., 2002, p. 21) and an indirect effect via enhanced perceptions of leader benevolence (good intentions and prosocial motives). This indirect effect was stronger when employees reported high *learning goal orientation* ("tendency to develop one's competence via engaging in challenging tasks and learning new skills," p. 4; Study 1 and 2) and *informational justice* ("perceptions of workplace information or explanations provided as clear, reasonable, and appropriately detailed," p. 5; Study 2).
Yang et al. (2019)	64 leaders,<br>278 followers	Time-lagged (2 waves, separated by 7 weeks)	Varied/multiple sectors (manufacturing, real estate, and technology)	China	Regression-based mediation and conditional-process analyses	Leaders were most likely to express humility with employees whom they perceived as highly capable. Leader-perceived employee capabilities had an indirect effect on follower trust in their leader via leader humility, but only when followers perceived their leader's expressed humility was unmotivated by impression management.

Citation	Sample(s)	Design	Organizational sector(s)	Country	Analytic approach	Key finding(s)
Ye et al. (2020)	137 leaders, 76 teams, 394 followers	Cross-sectional	Hospitality (hotel employees)	China	Hierarchical linear modeling	Leader humility was associated with higher collective humility (team humility), which was associated with higher employee creative performance (creativity). The effect of team humility on employee creative performance was stronger when teams were high in both competitive climate and *task interdependence* (“extent to which employees have to depend on their colleagues to complete tasks,” p. 2).
Yuan et al. (2018)	113 leaders, 515 followers	Time-lagged (2 waves, separated by 3 weeks)	Varied/multiple sectors (15 large companies [unspecified sectors])	China	Hierarchical regression analyses	Leader humility and employees’ creative process engagement exhibited a curvilinear relationship, such that leader humility enhanced creative process engagement up to a point, when excessive leader humility began contributing to lower employee creative process engagement (see Bin et al., 2021). Leader humility’s curvilinear effect on employee creative process engagement was mediated by *perceived organizational support* (“employees’ subjective perception of their organization’s support for their work, concern for their interests, and recognition of their value,” p. 471), but only when the leader’s perceived innovative competence was high.

Citation	Sample(s)	Design	Organizational sector(s)	Country	Analytic approach	Key finding(s)
Zapata & Hayes-Jones (2019)	Study 1:<br>326 undergraduate business students<br><br>Study 2:<br>318 undergraduate business students<br><br>Study 3:<br>219 undergraduate business students<br><br>Study 4:<br>168 undergraduate business students<br><br>Study 5:<br>289 undergraduate business students	Study 1:<br>Experimental<br><br>Study 2:<br>Experimental<br><br>Study 3:<br>Experimental<br><br>Study 4:<br>Experimental<br><br>Study 5:<br>Experimental	Study 1:<br>Controlled team performance task in a lab<br><br>Study 2:<br>Controlled team performance task in a lab<br><br>Study 3:<br>Fictional scenario<br><br>Study 4:<br>Fictional scenario<br><br>Study 5:<br>Fictional scenario	Study 1:<br>U.S.<br><br>Study 2:<br>U.S.<br><br>Study 3:<br>U.S.<br><br>Study 4:<br>U.S.<br><br>Study 5:<br>U.S.	Study 1:<br>Structural equation modeling<br><br>Study 2:<br>Structural equation modeling<br><br>Study 3:<br>Structural equation modeling<br><br>Study 4:<br>Structural equation modeling<br><br>Study 5:<br>Structural equation modeling	In Studies 1 and 2, leader humility led to lower perceptions of the leader's agentic characteristics, which contributed to lower perceived leader effectiveness and thereby to higher leader-undermining behavior.<br><br>This indirect effect of leader humility on decreased perceived leader effectiveness via lower perceived agentic characteristics was replicated in Studies 3, 4, and 5. However, in those studies, leader humility also contributed to higher perceptions of the leader's communal characteristics, which in turn contributed to higher leader effectiveness. In Study 5, the indirect effect on leader effectiveness via agentic characteristics only emerged in the context of leader's task success (a business acquisition).<br><br>In sum, these studies suggest leader humility may be a "double-edged sword" (p. 47) that has both costs (via lower perceptions of agentic characteristics) and benefits (via higher perceptions of communal characteristics). These costs and benefits can cancel each other out (cf. Owens et al., 2015).

Citation	Sample(s)	Design	Organizational sector(s)	Country	Analytic approach	Key finding(s)
Zhang & Liu (2019)	190 leaders, 190 followers	Time-lagged (2 waves, separated by 2 weeks)	Manufacturing	China	Regression-based mediation and conditional-process analyses	Leader humility had a direct effect on followers’ *taking-charge behavior* (“employees’ voluntary behavior that usually change[s] work processes, policies, and routines and aims at challenging current conditions,” p. 2) and an indirect effect on taking charge via enhanced *organization-based self-esteem* (“the extent to which organizational members recognize themselves as competent, important, and valuable,” p. 2). This indirect effect was stronger when followers perceived the leader as high in *leader prototypicality* (“degree to which leaders represent the group identity,” p. 4).
Zhong et al. (2020)	228 followers	Cross-sectional	Varied/multiple sectors (service, manufacturing, and government)	China	Regression-based mediation and conditional-process analyses	Leader humility had a direct positive effect on employee well-being (higher job satisfaction and job engagement). It also had an indirect effect on employee well-being via employees’ self-rated humility (follower humility). This indirect effect was stronger when followers perceived their leader as high (vs. low) in effectiveness. Leader humility also contributed to lower employee emotional exhaustion (burnout), but followers’ humility did not explain (mediate) that effect.

Table S2

Summary of Existing Correlates and Consequences of Leader Humility

Organizational outcomes	Team outcomes	Psychological outcomes
Higher follower job performance (Bin et al., 2021; Diao et al., 2019; Mao et al., 2019; Owens et al., 2013; Owens et al., 2015; Shannonhouse et al., 2019; L. Wang et al., 2018)	Higher team collective humility (Owens & Hekman, 2016; Rego et al., 2017; Ye et al., 2020)	Higher follower self-rated humility (Diao et al., 2019; Zhong et al., 2020)
Higher follower job engagement (Diao et al., 2019; X. Li et al., 2021; Luu, 2020; Nguyen et al., 2020; Owens & Hekman, 2012; Owens et al., 2013, 2015; Shannonhouse et al., 2019; Zhong et al., 2020)	Higher team performance (C. Chiu et al., 2016, 2022; Owens & Hekman, 2016; Peng et al., 2020; Rego et al., 2019; Shannonhouse et al., 2019; X. Wang et al., 2019)	Higher follower psychological capital (**h**ope, **e**fficacy, **r**esilience, **o**ptimism; “HERO” within; Bin et al., 2021)
Higher follower job satisfaction (Ou et al., 2017; Owens & Hekman, 2012; Owens et al., 2013; Zhong et al., 2020)	Higher team viability (performance capacity), team helping norms (prosocial behavior norms), and team performance (C. Chiu et al., 2022)	Higher follower self-expansion and self-efficacy (Mao et al., 2019)
Higher follower organizational citizenship (Nguyen et al., 2020; Tuan et al., 2021), prosocial (Carnevale et al., 2019; Mao et al., 2017; Owens et al., 2019), and ethical behaviors (Naseer et al., 2020; Owens et al., 2019) and higher leader altruism (Mallen et al., 2020)	Higher team member proactivity and competence (performance capacity; C. Chiu et al., 2016)	Higher follower creativity (Cheung et al., 2020; J. Wang et al., 2017), creative process engagement (Yuan et al., 2018), and creative performance (X. Li et al., 2021)
Lower follower workplace deviance (Quiyun et al., 2020) but higher follower workplace deviance when followers make self-serving attributions for their leader’s humility (Qin et al., 2020)	Higher team task interdependence (Liu et al., 2017)	Higher follower proactive behavior (Y. Chen et al., 2018; Ma et al., 2020; Zhang & Liu, 2019)
Higher follower compliance (T. Chiu & Hung, 2022) and feedback-seeking behavior (Qian et al., 2018)	Higher team task-allocation effectiveness (Rego et al., 2019)	Higher follower prosocial voice (Jeung & Yoon, 2018)
Higher follower commitment to the organization (X. Wang et al., 2022) and perceived organizational support (Yuan et al., 2018)	Higher team psychological safety (Hu et al., 2018; Rego et al., 2021; Swain, 2018; Walters & Diab, 2016)	Higher follower voice (X. Li et al., 2019; Lin et al., 2019; Ma et al., 2020; Mao et al., 2017)

Organizational outcomes	Team outcomes	Psychological outcomes
Higher follower job crafting (Tuan et al., 2021)	Higher team collective promotion focus (collective growth-mindedness; M. Li et al., 2019; X. Li et al., 2019; Owens & Hekman, 2016; X. Wang et al., 2022)	Higher attachment security (Bharanitharan et al., 2019) and relational closeness between followers and leaders (Mao et al., 2017)
Higher follower customer knowledge and adaptive self-efficacy (Luu, 2020)	Higher team cohesion (Shannonhouse et al., 2019)	Higher follower psychological entitlement (Qin et al., 2020)
Lower voluntary turnover (Ou et al., 2017; Owens et al., 2013)	Higher team creativity (C. Chen et al., 2021; X. Wang et al., 2019), sense of creative self-efficacy (X. Wang et al., 2019), creative performance (Ye et al., 2020), and innovation (Liu et al., 2017; Mallen et al., 2020)	Higher follower psychological empowerment (Y. Chen et al., 2018; Jeung & Yoon, 2016, 2018; Lin et al., 2019; Qiuyun et al., 2020; Shannonhouse et al., 2019)
Higher follower and leader adaptive selling behavior (Luu, 2020)	Higher team psychological capital (Rego et al., 2017, 2019)	Higher follower empathy, gratitude, perspective taking, and cognitive reappraisal (Naseer et al., 2020; J. Wang et al., 2017)
Higher follower identification with leader (Carnevale et al., 2019)	Higher shared leadership (C. Chiu et al., 2016)	Higher follower trait mindfulness (Cheung et al., 2020)
Higher perceived leader competence (Cojuharenco & Karelaia, 2020), effectiveness (Owens et al., 2015), and impact on team effectiveness (Rego et al., 2018)	Higher team job crafting (C. Chen et al., 2021)	Higher perceived meaningfulness of work (Shannonhouse et al., 2019)
Higher perceived leader innovative competence (Yuan et al., 2018)	Higher team learning (M. Li et al., 2019) and information sharing (Hu et al., 2018)	Higher follower authenticity (Oc et al., 2020)
Higher perceived leader potential (Swain & Korenman, 2018)	Higher team shared mental models (M. Li et al., 2019)	Higher follower organizational self-esteem (Zhang & Liu, 2019)
Higher perceived leader trustworthiness and perceived leader authority (T. Chiu & Hung, 2022; Rego et al., 2018)	Higher team voice climate (Liu et al., 2017)	Higher follower moral self-efficacy (Owens et al., 2019)

Organizational outcomes	Team outcomes	Psychological outcomes
Higher follower trust in leader (Cojuharenco & Karelaia, 2020; Nguyen et al., 2020; Owens & Hekman, 2012; Yang et al., 2019) and liking of the leader (Swain, 2018)	More positive relationships (friendships) among team members (C. Chiu et al., 2022)	Lower follower sense of vulnerability (Oc et al., 2020) and lower negative affect toward leader (Qin et al., 2021)
Higher perceived leader relational traits yet lower perceived leader agentic traits, which have counterbalancing effects on perceived leader competence (Zapata & Hayes-Jones, 2019)	Fewer negative relationships (conflictual) among team members (C. Chiu et al., 2022)	Higher follower relational energy (Ma et al., 2020; L. Wang et al., 2018) and lower follower emotional exhaustion (burnout; Shannonhouse et al., 2019; Zhong et al., 2020)
Higher perceived leader authenticity (sincerity; Ma et al., 2020; Oc et al., 2020; Owens & Hekman, 2012) and benevolence (X. Wang et al., 2022)	Higher perceived follower team contribution (Owens et al., 2013)	Higher leader trait narcissism in business and military leaders (L. Chen, 2018; Owens et al., 2015; Swain & Korenman, 2018) but lower trait narcissism in religious leaders (Jankowski et al., 2019)
Higher perceived leader empathy, kindness toward followers, and servant leadership behaviors (Krumrei-Mancuso, 2018)	Higher workplace spirituality (Naseer et al., 2020)	Higher leader positive mental health, lower negative mental health, and lower insecure attachment to God (religious attachment insecurity; Jankowski et al., 2019; Ruffing et al., 2021)

Table S3

Hierarchical Regression Results for Organizational Outcomes Among Humanitarian Aid Workers

Variable	Leader effectiveness				Leader impact on team				Team cohesion				Team psychological safety			
	R^2	ΔR^2	β	*sr*	R^2	ΔR^2	β	*sr*	R^2	ΔR^2	β	*sr*	R^2	ΔR^2	β	*sr*
Step 1: Sociodemographics	**.05**	**.05**			**.06**	**.06**			.04	.04			.02	.02		
Worker age			.02	.02			.04	.04			.04	.04			.14	.13
Worker gender (1 = female)			**-.17**	**-.17**			-.14	-.14			**-.18**	**-.17**			.05	.05
Worker time at organization			-.14	-.11			**-.22**	**-.18**			-.08	-.06			-.01	-.01
Worker time under leader			-.01	-.01			.08	.07			.07	.06			.01	.01
Step 2: Team cohesion/safety	**.24**	**.19**			**.28**	**.22**			--	--			--	--		
Team cohesion			**.42**	**.35**			**.48**	**.40**			--	--			--	--
Team psychological safety			.04	.04			-.01	-.01			--	--			--	--
Step 3: Leader/team humility	**.49**	**.25**			**.60**	**.32**			**.53**	**.49**			**.31**	**.29**		
Leader general humility			**.35**	**.22**			**.47**	**.30**			**.25**	**.17**			-.05	-.03
Leader relational humility			**.26**	**.17**			**.22**	**.14**			-.09	-.06			**.32**	**.21**
Worker general humility			.09	.08			.01	.01			.03	.02			-.07	-.06
Worker relational humility			-.03	-.03			.01	.01			.07	.06			-.02	-.02
Team collective humility			-.02	-.01			-.09	-.06			**.58**	**.48**			**.42**	**.35**

Note. N = 308. *sr* = semipartial (part) correlation coefficient. Statistics that are significant at $p < .005$ are indicated in boldface type (Bonferroni correction across the 11 tests .05/11 = .005).

Table S3

Hierarchical Regression Results for Organizational Outcomes Among Humanitarian Aid Workers (cont'd)

Variable	Worker job engagement				Worker job satisfaction				Team performance				Team collective promotion focus			
	R^2	ΔR^2	β	*sr*	R^2	ΔR^2	β	*sr*	R^2	ΔR^2	β	*sr*	R^2	ΔR^2	β	*sr*
Step 1: Sociodemographics	**.08**	**.08**			.02	.02			.03	.03			**.05**	**.05**		
Worker age			.08	.08			.01	.01			-.04	-.04			.05	.04
Worker gender (1 = female)			**-.19**	**-.19**			-.11	-.11			-.09	-.09			**-.21**	**-.20**
Worker time at organization			**-.19**	**-.16**			-.03	-.03			-.12	-.10			-.04	-.04
Worker time under leader			.11	.09			.05	.05			.01	.00			.02	.01
Step 2: Team cohesion/safety	**.23**	**.15**			**.25**	**.23**			**.27**	**.24**			**.44**	**.39**		
Team cohesion			**.43**	**.36**			**.40**	**.34**			**.52**	**.43**			**.65**	**.54**
Team psychological safety			-.08	-.07			.14	.12			-.04	-.03			-.02	-.01
Step 3: Leader/team humility	**.30**	**.07**			**.39**	**.15**			**.31**	**.04**			**.47**	.03		
Leader general humility			.11	.07			**.27**	**.17**			.03	.02			.10	.06
Leader relational humility			.02	.01			.10	.06			.08	.05			-.06	-.04
Worker general humility			**.24**	**.20**			.08	.07			.12	.10			.06	.05
Worker relational humility			.05	.04			-.01	-.01			.03	.02			.04	.04
Team collective humility			-.04	-.03			**.24**	**.16**			.13	.09			.15	.10

Note. *N* = 308. *sr* = semipartial (part) correlation coefficient. Statistics that are significant at *p* < .005 are indicated in boldface type (Bonferroni correction across the 11 analyses .05/11 = .005).

Table S4

Hierarchical Regression Results for Psychological Outcomes Among Humanitarian Aid Workers

Variable	Depression symptoms				Anxiety symptoms				PTSD symptoms				Secondary traumatic stress			
	R^2	ΔR^2	β	*sr*	R^2	ΔR^2	β	*sr*	R^2	ΔR^2	β	*sr*	R^2	ΔR^2	β	*sr*
Step 1: Sociodemographics	.02	.02			.02	.02			.02	.02			**.09**	**.09**		
Worker age			-.13	-.12			-.14	-.13			-.08	-.07			-.03	-.03
Worker gender (1 = female)			.07	.07			.06	.06			-.10	-.09			**-.26**	**-.26**
Worker time at organization			-.01	-.01			.00	.00			.01	.01			-.01	-.01
Worker time under leader			.01	.01			.02	.02			-.05	-.05			-.14	-.12
Step 2: Team cohesion/safety	**.07**	**.04**			**.06**	**.04**			**.05**	**.03**			**.15**	**.07**		
Team cohesion			**-.21**	**-.17**			-.11	-.09			-.01	-.01			**.28**	**.24**
Team psychological safety			-.01	-.01			-.11	-.10			-.18	-.15			**-.26**	**-.22**
Step 3: Leader/team humility	**.10**	.03			**.08**	.02			**.12**	**.07**			**.18**	.03		
Leader general humility			-.08	-.05			-.03	-.02			-.08	-.05			.09	.06
Leader relational humility			.06	.04			.01	.01			-.08	-.05			-.11	-.07
Worker general humility			-.01	-.01			.00	.00			.09	.08			.03	.02
Worker relational humility			**-.19**	**-.16**			-.14	-.12			**-.19**	**-.17**			-.05	-.04
Team collective humility			.01	.00			.06	.04			.12	.08			.18	.12

Note. *N* = 308. *sr* = semipartial (part) correlation coefficient. Statistics that are significant at $p < .005$ are indicated in boldface type (Bonferroni correction across the 11 tests .05/11 = .005).

Table S4

Hierarchical Regression Results for Psychological Outcomes Among Humanitarian Aid Workers (cont'd)

Variable	Burnout				Compassion satisfaction				Perceived PTG				Psychological flourishing			
	R^2	ΔR^2	β	*sr*	R^2	ΔR^2	β	*sr*	R^2	ΔR^2	β	*sr*	R^2	ΔR^2	β	*sr*
Step 1: Sociodemographics	.00	.00			**.05**	**.05**			.03	.03			.02	.02		
Worker age			-.04	-.04			-.04	-.04			.01	.01			.01	.01
Worker gender (1 = female)			-.01	-.01			**-.21**	**-.21**			-.14	-.14			-.16	-.16
Worker time at organization			.04	.03			-.01	-.01			-.04	-.03			.03	.02
Worker time under leader			-.05	-.04			-.01	-.01			-.05	-.05			-.04	-.04
Step 2: Team cohesion/safety	**.08**	**.08**			**.20**	**.15**			**.10**	**.08**			**.11**	**.08**		
Team cohesion			-.15	-.13			**.44**	**.37**			**.33**	**.28**			**.29**	**.24**
Team psychological safety			-.18	-.15			-.10	-.08			**-.21**	**-.18**			.00	.00
Step 3: Leader/team humility	**.21**	**.13**			**.32**	**.12**			**.16**	**.06**			**.17**	**.07**		
Leader general humility			-.08	-.05			.11	.07			.04	.03			.05	.03
Leader relational humility			.09	.06			-.19	-.12			-.18	-.12			-.16	-.10
Worker general humility			**-.18**	**-.15**			**.33**	**.28**			.16	.14			.13	.11
Worker relational humility			**-.29**	**-.26**			.12	.11			.15	.13			**.19**	**.17**
Team collective humility			.03	.02			-.07	-.05			-.04	-.02			.07	.04

Note. $N = 308$. *sr* = semipartial (part) correlation coefficient; PTG = posttraumatic growth. Statistics that are significant at $p < .005$ are indicated in boldface type (Bonferroni correction across the 11 analyses .05/11 = .005).

Table S5

Hierarchical Regression Results for Organizational Outcomes Among Humanitarian Aid Leaders

Variable	Leader job engagement				Leader job satisfaction				Team cohesion				Team psychological safety			
	R^2	ΔR^2	β	*sr*	R^2	ΔR^2	β	*sr*	R^2	ΔR^2	β	*sr*	R^2	ΔR^2	β	*sr*
Step 1: Sociodemographics	.02	.02			.06	.06			.04	.04			.08	.08		
Leader age			-.03	-.03			-.05	-.04			.10	.09			**.23**	**.21**
Leader gender (1 = female)			-.14	-.14			**-.23**	**-.22**			-.10	-.10			.13	.13
Leader time at organization			.04	.04			.12	.09			-.11	-.09			.03	.02
Leader time in leader role			-.02	-.01			-.10	-.08			.15	.13			-.01	-.01
Step 2: Team cohesion/safety	**.09**	**.07**			**.19**	**.13**			--	--			--	--		
Team cohesion			.22	.20			**.27**	**.25**			--	--			--	--
Team psychological safety			.09	.08			.17	.15			--	--			--	--
Step 3: Leader/team humility	**.24**	**.15**			**.28**	**.09**			**.34**	**.30**			**.23**	**.15**		
Leader general humility			**.33**	**.29**			**.22**	**.19**			.05	.05			.15	.14
Leader relational humility			-.01	-.01			-.05	-.04			.11	.10			.09	.08
Team collective humility			.24	.19			**.25**	**.20**			**.52**	**.48**			**.29**	**.27**

Table S5

Hierarchical Regression Results for Organizational Outcomes Among Humanitarian Aid Leaders (cont’d)

Variable	Workers’ performance				Team performance				Team collective promotion focus			
	R^2	ΔR^2	β	*sr*	R^2	ΔR^2	β	*sr*	R^2	ΔR^2	β	*sr*
Step 1: Sociodemographics	.02	.02			.01	.01			.06	.06		
Leader age			**.14**	**.13**			.01	.01			.03	.03
Leader gender (1 = female)			-.04	-.04			.00	.00			-.19	-.18
Leader time at organization			-.08	-.07			-.03	-.03			.00	.00
Leader time in current leader role			.01	.01			.11	.09			.12	.10
Step 2: Team cohesion/safety	**.17**	**.15**			**.12**	**.11**			**.25**	**.19**		
Team cohesion			**.34**	**.31**			**.30**	**.28**			**.40**	**.37**
Team psychological safety			.12	.11			.07	.06			.09	.08
Step 3: Leader/team humility	**.20**	.03			**.16**	.04			**.36**	**.12**		
Leader general humility (self-reported)			.04	.04			.10	.09			**.24**	**.21**
Leader relational humility (self-reported)			-.02	-.02			-.02	-.02			-.02	-.02
Team collective humility			.19	.15			.21	.16			**.28**	**.22**

Note. N = 167. *sr* = semipartial (part) correlation coefficient. Statistics that are significant at $p < .006$ are indicated in boldface type (Bonferroni correction across the 9 analyses .05/9 = .006).

Table S6

Hierarchical Regression Results for Psychological Outcomes Among Humanitarian Aid Leaders

Variable	Depression symptoms				Anxiety symptoms				PTSD symptoms				Secondary traumatic stress			
	R^2	ΔR^2	β	*sr*	R^2	ΔR^2	β	*sr*	R^2	ΔR^2	β	*sr*	R^2	ΔR^2	β	*sr*
Step 1: Sociodemographics	**.09**	**.09**			**.09**	**.09**			.05	.05			**.13**	**.13**		
Leader age			-.21	-.19			-.20	-.18			**-.23**	**-.21**			**-.32**	**-.29**
Leader gender (1 = female)			**.23**	**.23**			.17	.17			.01	.01			-.06	-.06
Leader time at organization			.02	.01			.01	.01			.02	.01			-.09	-.07
Leader time in leader role			.14	.12			**.25**	**.21**			.17	.14			**.30**	**.25**
Step 2: Team cohesion/safety	**.14**	.05			**.13**	.04			**.12**	.07			**.18**	.05		
Team cohesion			-.16	-.15			-.19	-.18			-.11	-.10			.10	.09
Team psychological safety			-.12	-.10			-.03	-.03			-.21	-.18			**-.25**	**-.23**
Step 3: Leader/team humility	**.16**	.02			**.14**	.01			**.14**	.02			**.27**	**.09**		
Leader general humility			-.02	-.02			.04	.03			.00	.00			**.31**	**.28**
Leader relational humility			-.14	-.13			-.09	-.09			-.12	-.11			-.07	-.07
Team collective humility			-.03	-.02			-.05	-.04			-.08	-.07			.05	.04

Note. N = 167. *sr* = semipartial (part) correlation coefficient. Statistics that are significant at $p < .006$ are indicated in boldface type (Bonferroni correction across the 9 analyses .05/9 = .006).

Table S6

Hierarchical Regression Results for Psychological Outcomes Among Humanitarian Aid Leaders (cont’d)

Variable	Burnout				Compassion satisfaction				Perceived PTG				Psychological flourishing			
	R^2	ΔR^2	β	*sr*	R^2	ΔR^2	β	*sr*	R^2	ΔR^2	β	*sr*	R^2	ΔR^2	β	*sr*
Step 1: Sociodemographics	.06	.06			.05	.05			.06	.06			.03	.03		
Leader age			-.14	-.12			-.01	-.01			-.06	-.06			.09	.08
Leader gender (1 = female)			.21	.21			-.16	-.16			-.01	-.01			-.03	-.03
Leader time at organization			.02	.01			-.06	-.05			.17	.14			.12	.10
Leader time in leader role			-.01	-.01			.15	.13			.13	.11			.02	.01
Step 2: Team cohesion/safety	**.17**	**.11**			**.18**	**.13**			.08	.02			**.11**	**.07**		
Team cohesion			**-.28**	**-.25**			**.34**	**.31**			.14	.13			.22	.20
Team psychological safety			-.11	-.09			.06	.06			-.11	-.10			.11	.10
Step 3: Leader/team humility	**.23**	.06			**.34**	**.16**			.11	.03			**.14**	.03		
Leader general humility			**-.22**	**-.20**			**.36**	**.32**			.19	.17			.18	.16
Leader relational humility			-.06	-.06			.01	.01			-.03	-.03			-.02	-.01
Team collective humility			-.06	-.05			.19	.15			.04	.03			.01	.01

Note. *N* = 167. *sr* = semipartial (part) correlation coefficient. Statistics that are significant at $p < .006$ are indicated in boldface type (Bonferroni correction across the 9 analyses .05/9 = .006).

Table S7

Prevalence Rates of Likely Mental Health Disorders Based on Leader Status, Personnel Category, and Region of Field Work

	N (%)	Major Depressive Disorder (MDD) likely		Generalized Anxiety Disorder (GAD) likely		Post-Traumatic Stress Disorder (PTSD) likely	
		n	%	*n*	%	*n*	%
Overall sample	475 (100.0)	48	10.1	30	6.3	31	6.5
Leader status							
Humanitarian aid leader	167 (35.2)	17	10.2	8	4.8	8	4.8
Humanitarian aid worker	308 (64.8)	31	10.1	22	7.1	23	7.5
χ^2		$\chi^2(1) = 0.00$, $p = 1.000$		$\chi^2(1) = 1.29$, $p = .256$		$\chi^2(1) = 1.27$, $p = .260$	
Gender identity							
Male	259 (54.5)	20	7.7	16	6.2	18	6.9
Female	216 (45.5)	28	13.0	14	6.5	13	6.0
χ^2		$\chi^2(1) = 3.02$, $p = .082$		$\chi^2(1) = 0.00$, $p = 1.000$		$\chi^2(1) = 0.13$, $p = .718$	
Personnel category							
Nationally recruited personnel	250 (52.6)	23	9.2	15	6.0	21	8.4
Internationally recruited personnel	119 (25.1)	18	15.1	9	7.6	8	6.7
Headquarters personnel	106 (22.3)	7	6.6	6	5.7	2	1.9
χ^2		$\chi^2(2) = 4.62$, $p = .100$		$\chi^2(2) = 0.78$, $p = .678$		$\chi^2(2) = 5.13$, $p = .077$	
Region of fieldwork							
Africa	161 (33.9)	14	8.7	9	5.6	13	8.1
Asia	71 (15.0)	4	5.6	4	5.6	4	5.6
Middle East	143 (30.1)	23	16.1	10	7.0	12	8.4
χ^2		$\chi^2(2) = 5.59$, $p = .061$		$\chi^2(2) = 0.21$, $p = .900$		$\chi^2(2) = 0.84$, $p = .657$	

Note. A diagnosis of Major Depressive Disorder was deemed likely based on a Patient Health Questionnaire–9 (PHQ-9; Kroenke et al., 2001) total score of 10 or above, as per the standard recommended cutoff score of 10 (Manea et al., 2012). A diagnosis of Generalized Anxiety Disorder was deemed likely based on a Generalized Anxiety Disorder–7 (GAD-7; Spitzer et al., 2006) total score of 10 or above, as per the standard recommended cutoff score of 10 (Spitzer et al., 2006). A diagnosis of PTSD was deemed likely based on a PTSD CheckList for DSM-5 8-item version (PCL5 8-item; Price et al., 2016) total score of 19 or above, as per the standard recommended cutoff score of 19 (Price et al., 2016).

Table S8

Descriptive Statistics and Intercorrelations for Individual-Level Variables (Level-1, Worker-Rated Variables)

Variable	1	2	3	4	5	6	7	8	9	10	11	12	13	14	15
1. Worker general humility															
2. Worker relational humility	**.37**														
3. Perceived leader effectiveness	**.28**	.22													
4. Perceived leader impact on team	.23	**.30**	**.74**												
5. Worker job engagement	**.48**	**.26**	**.30**	**.38**											
6. Worker job satisfaction	**.36**	**.24**	**.43**	**.53**	**.55**										
7. Team performance	**.39**	**.28**	**.51**	**.49**	**.43**	**.53**									
8. Team cohesion	**.37**	**.26**	**.43**	**.54**	**.47**	**.57**	**.59**								
9. Team psychological safety	.14	**.24**	**.32**	**.31**	.17	**.30**	.22	**.49**							
10. Secondary traumatic stress	.15	-.07	.16	.18	**.33**	-.23	**.25**	.16	**-.24**						
11. Burnout	**-.40**	**-.45**	-.12	-.13	**-.29**	**-.28**	-.23	-.23	**-.29**	.22					
12. Compassion satisfaction	**.54**	**.25**	.16	.21	**.49**	**.34**	**.41**	**.37**	.07	**.38**	**-.53**				
13. Depression symptoms	-.20	-.19	**-.25**	.19	**-.28**	**-.42**	**-.32**	**-.29**	-.14	-.03	**.37**	**-.25**			
14. PTSD symptoms	.06	-.21	-.08	-.06	.01	-.14	-.04	-.11	-.19	**.40**	**.27**	.08	**.44**		
15. Psychological flourishing	**.37**	**.26**	.23	.20	**.31**	**.28**	**.32**	**.36**	.13	.15	**-.43**	**.52**	**-.39**	-.11	
M	4.49	3.99	3.86	5.17	4.71	5.17	3.69	5.61	4.91	20.36	20.34	41.08	5.91	3.96	8.02
SD	0.44	0.48	0.93	1.74	1.02	0.97	0.73	1.18	0.94	7.18	5.05	6.69	0.95	4.10	6.44
Range	1–5	1–5	1–5	1–7	1–6	1–7	1–5	1–7	1–5	10–50	10–50	10–50	1–7	0–27	0–32

Note. $N = 189$. PTSD = Posttraumatic Stress Disorder. Correlations that are significant at $p \leq .001$ are indicated in boldface type.

Table S9

Descriptive Statistics and Intercorrelations for Leader- and Team-Level Variables (Level-2 Variables)

Variable	*M*	*SD*	Range	1	2	3	4	5	6
1. Leader self-rated general humility	4.43	0.50	1–5						
2. Leader self-rated relational humility	3.86	0.42	1–5	.27					
3. Leader self-rated trait narcissism	4.03	2.73	0–10	.01	-.16				
4. Team-rated leader general humility†	3.95	0.79	1–5	.03	-.19	.04			
5. Team-rated leader relational humility†	3.78	0.61	1–5	.01	-.11	-.12	**.85**		
6. Team-rated collective humility†	4.18	0.95	1–5	-.11	-.16	.07	**.68**	**.67**	
7. Perceived leader impact†	5.17	1.74	1–7	.14	-.05	.15	**.60**	**.47**	.32
8. Perceived leader effectiveness†	3.86	0.93	1–5	.13	-.09	.16	**.56**	**.42**	.32
9. Team cohesion†	5.61	1.18	1–7	-.01	-.11	.18	**.41**	.24	**.49**
10. Team psychological safety†	4.91	0.94	1–5	-.04	.01	.08	**.40**	**.42**	.27
11. Team performance†	3.69	0.73	1–5	.01	-.10	.16	.32	.16	.31
12. Job engagement†	4.71	1.02	1–6	-.04	.00	.19	.22	.12	**.37**
13. Job satisfaction†	5.17	0.97	1–7	.07	.07	.24	**.41**	.28	**.39**
14. Compassion satisfaction†	41.08	6.69	10–50	.02	-.08	.05	.09	.00	.22
15. Burnout†	20.34	5.05	10–50	-.09	-.06	-.11	-.09	-.04	-.16
16. Secondary traumatic stress†	20.36	7.18	10–50	.04	.05	.00	.03	.04	.07
17. Depression symptoms†	3.96	4.10	0–27	-.06	.00	-.22	-.17	-.04	-.20
18. PTSD symptoms†	8.02	6.44	0–32	-.06	.00	-.17	-.08	-.07	.03
19. Psychological flourishing†	5.91	0.95	1–7	.23	-.06	.20	.00	-.02	.11

Note. $N = 96$. Intercorrelations for columns 7 through 19 are not reported because they are either not as interpretable or not as relevant to the current study. Correlations that are significant at $p \leq .001$ are indicated in boldface type.
† = This variable is measured at the individual level but is reported here at the team level (specifically as the aggregate-mean score for all surveyed team members); therefore, this variable’s correlations should be interpreted as the influence of the respective leader- or team-rated variable on team-level outcomes.

References

Bharanitharan, K., Chen, Z. X., Bahmannia, S., & Lowe, K. B. (2019). Is leader humility a friend or foe, or both? An attachment theory lens on leader humility and its contradictory outcomes. *Journal of Business Ethics, 160*(3), 729–743. https://doi.org/10.1007/s10551-018-3925-z

Bin, D., Sok, K. M., Sok, P., & Mao, S. (2021). The tipping point: Mitigating the curvilinear effect of frontline service employees’ perception of leadership humility on frontline service performance. *Journal of Service Theory and Practice, 31*(1), 137–156. http://dx.doi.org/10.1108/JSTP-06-2020-0110

Carnevale, J. B., Huang, L., & Paterson, T. (2019). LMX-differentiation strengthens the prosocial consequences of leader humility: An identification and social exchange perspective. *Journal of Business Research*, *96*, 287–296. https://doi.org/10.1016/j.jbusres.2018.11.048

Chandler, J., Johnson, N., Jordan, S., Darren, K., & Short, J. (2023). A meta-analysis of humble leadership: Reviewing individual, team, and organizational outcomes of leader humility. *Leadership Quarterly*, *34*, 101660. https://doi.org/10.1016/j.leaqua.2022.101660

Chen, C., Feng, J., Liu, X., & Yao, J. (2021). Leader humility, team job crafting and team creativity: The moderating role of leader–leader exchange. *Human Resource Management Journal*, *31*(1), 326–340. https://doi.org/10.1111/1748-8583.12306

Chen, L. (2018). Thinking paradoxically: How and when female narcissistic leaders integrate gender–leader identities. *Social Behavior and Personality*, *46*(2), 339–352. https://doi.org/10.2224/sbp.6910

Chen, Y., Liu, B., Zhang, L., & Qian, S. (2018). Can leader “humility” spark employee “proactivity”? The mediating role of psychological empowerment. *Leadership & Organization Development Journal*, *39*(3), 326–339. https://doi.org/10.1108/LODJ-10-2017-0307

Cheung, S. Y., Huang, E. G., Chang, S., & Wei, L. (2020). Does being mindful make people more creative at work? The role of creative process engagement and perceived leader humility. *Organizational Behavior and Human Decision Processes*, *159*, 39–48. https://doi.org/10.1016/j.obhdp.2019.12.003

Chiu, C. Y., Balkundi, P., Owens, B. P., & Tesluk, P. E. (2022). Shaping positive and negative ties to improve team effectiveness: The roles of leader humility and team helping norms. *Human Relations*, *75*(3), 502–531. https://doi.org/10.1177/0018726720968135

Chiu, C. Y., Owens, B. P., & Tesluk, P. E. (2016). Initiating and utilizing shared leadership in teams: The role of leader humility, team proactive personality, and team performance capability. *Journal of Applied Psychology*, *101*(12), 1705–1720. http://dx.doi.org/10.1037/apl0000159

Chiu, C. Y., Marrone, J. A., & Tuckey, M. R. (2021). How do humble people mitigate group incivility? An examination of the social oil hypothesis of collective humility. *Journal of Occupational Health Psychology*, *26*(5), 361–373. http://dx.doi.org/10.1037/ocp0000244

Chiu, T. C., & Hung, Y. (2022). Impacts of leader humility between authority and trustworthiness on compliance: Tests of three-way interaction. *Psychological Reports*, *125*(1), 398–421. https://doi.org/10.1177/0033294120973942

Cojuharenco, I., & Karelaia, N. (2020). When leaders ask questions: Can humility premiums buffer the effects of competence penalties? *Organizational Behavior and Human Decision Processes*, *156*, 113–134. https://doi.org/10.1016/j.obhdp.2019.12.001

Cuthbert, A. D., Davis, E. B., Aten, J. D., Short, A., Yarborough, C. A., . . . Van Tongeren, D. R. (2018). Cultivating humility in religious leaders: The effectiveness of a spiritually integrated positive psychology intervention. *Spirituality in Clinical Practice*, *5*(4), 227–239. http://dx.doi.org/10.1037/scp0000185

Davis, E. B., Plante, T. G., Grey, M. J., Kim, C. L., Freeman-Coppadge, D., Lefevor, T., Paulez, J. A., Giwa, S., Lasser, J., Stratton, S. P., Deneke, E., & Glowiak, K.* (2021). The role of civility and cultural humility in navigating controversial areas in psychology. *Spirituality in Clinical Practice*, *8*(2), 79–87. http://dx.doi.org/10.1037/scp0000236

Diao, H., Song, L. J., Wang, Y., & Zhong, J. (2019). Being passionate to perform: The joint effect of leader humility and follower humility. *Frontiers in Psychology*, *10*, Article 1059. https://doi.org/10.3389/fpsyg.2019.01059

Hannah, S. T., Avolio, B. J., & May, D. R. (2011). Moral maturation and moral conation: A capacity approach to explaining moral thought and action. *Academy of Management Review*, *36*(4), 663–685. https://www.jstor.org/stable/41318090

Hu, J., Erdogan, B., Jiang, K., Bauer, T. N., & Liu, S. (2018). Leader humility and team creativity: The role of team information sharing, psychological safety, and power distance. *Journal of Applied Psychology*, *103*(3), 313–323. http://dx.doi.org/10.1037/apl0000277

Jankowski, P. J., Sandage, S. J., Bell, C. A., Ruffing, E. G., & Adams, C. (2019). Humility, relational spirituality, and well-being among religious leaders: A moderated mediation

model. *Journal of Religion and Health*, *58*(1), 132–152. http://dx.doi.org/10.1007/s10943-018-0580-8

Jeung, C.-W., & Yoon, H. J. (2016). Leader humility and psychological empowerment: Investigating contingencies. *Journal of Managerial Psychology*, *31*(7), 1122–1136. https://doi.org/10.1108/JMP-07-2015-0270

Jeung, C.-W., & Yoon, H. J. (2018). When leadership elicits voice: Evidence for a mediated moderation model. *Journal of Management & Organization*, *24*(1), 40–61. https://doi.org/jmo.2017.42

Johnson, R. E., Selenta, C., & Lord, R. G. (2006). When organizational justice and the self-concept meet: Consequences for the organization and its members. *Organizational Behavior and Human Decision Processes*, *99*(2), 175–201. https://doi.org/10.1016/j.obhdp.2005.07.005

Kroenke, K., Spitzer, R. L., & Williams, J. B. W. (2001). The PHQ-9: Validity of a brief depression severity measure. *Journal of General Internal Medicine*, *16*(9), 606–613. https://doi.org/10.1046/j.1525-1497.2001.016009606.x

Krumrei-Mancuso, E. J. (2018). Humility in servant leadership among Christian student leaders: A longitudinal pilot study. *Journal of Psychology and Theology*, *46*(4), 253–267. http://dx.doi.org/10.1177/0091647118807177

Li, M., Zhang, P., Xia, Y., & Liu, W. (2019). Shaping the shared mental model: How leader humility helps teams to learn. *Journal of Management & Organization*, *25*(5), 653–671. https://doi.org/10.1017/jmo.2017.21

Li, X., Li, M., Fu, J., & Ullah, A. (2019). Leader humility and employee voice: The role of employees’ regulatory focus and voice–role conception. *Social Behavior and Personality*, *47*(6), Article e7811. https://doi.org/10.2224/sbp.7811

Li, X., Xue, J., & Liu, J. (2021). Linking leader humility to employee creative performance: Work engagement as a mediator. *Social Behavior and Personality*, *49*(6), Article e10358. https://doi.org/10.2224/sbp.10358

Lie, D. A., Forest, C. P., Walsh, A., Banzali, Y., & Lohenry, K. (2016). What and how do students learn in an interprofessional student-run clinic? An educational framework for team-based care. *Medical Education Online*, *21*, Article 31900. https://doi.org/10.3402/meo.v21.31900

Lin, X., Chen, Z. X., Tse, H. H. M., Wei, W., & Ma, C. (2019). Why and when employees like to speak up more under humble leaders? The roles of personal sense of power and power distance. *Journal of Business Ethics*, *158*, 937–950. https://doi.org/10.1007/s10551-017-3704-2

Liu, W., Mao, J., & Chen, X. (2017). Leader humility and team innovation: Investigating the substituting role of task interdependence and the mediating role of team voice climate. *Frontiers in Psychology*, *8*, Article 1115. https://doi.org/10.3389/fpsyg.2017.01115

Luthans, F., & Youssef-Morgan, C. M. (2017). Psychological capital: An evidence-based positive approach. *Annual Review of Organizational Psychology and Organizational Behavior*, *4*, 339–366. https://doi.org/10.1146/annurev-orgpsych-032516-113324

Luu, T. T. (2020). Can sales leaders with humility create adaptive retail salespersons. *Psychology & Marketing*, *37*(9), 1292–1315. https://doi.org/10.1002/mar.21365

Ma, C., Wu, C.-H., Chen, Z. X., Jiang, X., & Wei, W. (2020). Why and when leader humility promotes constructive voice: A crossover of energy perspective. *Personnel Review*, *49*(5), 1157–1175. https://doi.org/10.1108/PR-02-2019-0049

Mallen, F., Dominguez-Escrig, E., Lapiedra, R., & Chiva, R. (2020). Does leader humility matter? Effects on altruism and innovation. *Management Decision*, *58*(5), 967–981. http://dx.doi.org/10.1108/MD-11-2018-1180

Manea, L., Gilbody, S., & McMillan, D. (2012). Optimal cut-off score for diagnosing depression with the Patient Health Questionnaire (PHQ-9): A meta-analysis. *CMAJ*, *184*(3), E191–E196. https://doi.org/10.1503/cmaj.110829

Mao, J., Chiu, C. Y., Owens, B. P., Brown, J. A., & Liao, J. (2019). Growing followers: Exploring the effects of leader humility on follower self-expansion, self-efficacy, and performance. *Journal of Management Studies*, *56*(2), 343–371. https://doi.org/10.1111/joms.12395

Mao, J., Liao, J., Han, Y., & Liu, W. (2017). The mechanism and effect of leader humility: An interpersonal relationship perspective. *Acta Psychologica Sinica*, *49*(9), 1219–1233. https://doi.org/10.3724/SP.J.1041.2017.01219

Meyer, J. P., Stanley, D. J., Herscovitch, L., & Topolnytsky, L. (2002). Affective, continuance, and normative commitment to the organization: A meta-analysis of antecedents, correlates, and consequences. *Journal of Vocational Behavior*, *61*(1), 20–52. https://doi.org/10.1006/jvbe.2001.1842

Naseer, S., Syed, F., Nauman, S., Fatima, T., Jameel, I., & Riaz, N. (2020). Understanding how leaders’ humility promotes followers’ emotions and ethical behaviors: Workplace

spirituality as a mediator. *Journal of Positive Psychology*, *15*(3), 407–419. https://doi.org/10.1080/17439760.2019.1615103

Nguyen, D. T. N., Teo, S. T. T., Halvorsen, B., & Staples, W. (2020). Leader humility and knowledge sharing intention: A serial mediation model. *Frontiers in Psychology, 11*, Article 560704. https://doi.org/10.3389/fpsyg.2020.560704

Oc, B., Bashshur, M. R., Daniels, M. A., Greguras, G. J., & Diefendorff, J. M. (2015). Leader humility in Singapore. *The Leadership Quarterly*, *26*(1), 68–80. https://doi.org/10.1016/j.leaqua.2014.11.005

Oc, B., Daniels, M. A., Diefendorff, J. M., Bashshur, M. R., & Greguras, G. J. (2020). Humility breeds authenticity: How authentic leader humility shapes follower vulnerability and felt authenticity. *Organizational Behavior and Human Decision Processes, 158*, 112–125. https://doi.org/10.1016/j.obhdp.2019.04.008

Ou, A. Y., Seo, J., Choi, D., & Hom, P. (2017). When can humble top executives retain middle managers? The moderating role of top management team faultlines. *Academy of Management Journal*, *60*(5), 1915–1931. https://doi.org/10.5465/amj.2015.1072

Owens, B., & Hekman, D. (2012). Modeling how to grow: An inductive examination of humble leader behaviors, contingencies, and outcomes. *Academy of Management Journal*, *55*(4), 787–818. https://doi.org/10.5465/amj.2010.0441

Owens, B., & Hekman, D. (2016). How does leader humility influence team performance? Exploring the mechanisms of contagion and collective promotion focus. *Academy of Management Journal*, *59*(3), 1088–1111. https://doi.org/10.5465/amj.2013.0660

Owens, B. P., Johnson, M. D., & Mitchell, T. R. (2013). Expressed humility in organizations: Implications for performance, teams, and leadership. *Organization Science*, *24*(5), 1517–1538. https://doi.org/10.1287/orsc.1120.0795

Owens, B. P., Wallace, A. S., & Waldman, D. A. (2015). Leader narcissism and follower outcomes: The counterbalancing effect of leader humility. *Journal of Applied Psychology*, *100*(4), 1203–1213. https://doi.org/10.1037/a0038698

Owens, B. P., Yam, K. C., Bednar, J. S., Mao, J., & Hart, D. W. (2019). The impact of leader moral humility on follower moral self-efficacy and behavior. *Journal of Applied Psychology,104*(1), 146–163. https://doi.org/10.1037/apl0000353

Peng, A. C., Wang, B., Schaubroeck, J. M., & Gao, R. (2020). Can humble leaders get results? The indirect and contextual influences of skip-level leaders. *Journal of Leadership & Organizational Studies*, *27*(4), 329–339. https://doi.org/10.1177%2F1548051820952402

Price, M., Szafranski, D. D., van Stolk-Cooke, K., & Gros, D. (2016). Investigation of abbreviated 4 and 8 item versions of the PTSD Checklist 5. *Psychiatry Research, 239*, 124–130. https://doi.org/10.1016/j.psychres.2016.03.014

Qian, J., Li, X., Song, B., Wang, B., Wang, M., Chang, S., & Xiong, Y. (2018). Leaders’ expressed humility and followers’ feedback seeking: The mediating effects of perceived image cost and moderating effects of power distance orientation. *Frontiers in Psychology*, *9*, Article 563. https://doi.org/10.3389/fpsyg.2018.00563

Qin, X., Chen, C., Yam, K. C., Huang, M., & Ju, D. (2020). The double-edged sword of leader humility: Investigating when and why leader humility promotes versus inhibits subordinate deviance. *Journal of Applied Psychology, 105*(7), 693–712. https://doi.org/10.1037/apl0000456

Qin, X., Liu, X., Brown, J. A., Zheng, X., & Owens, B. P. (2021). Humility harmonized? Exploring whether and how leader and employee humility (in)congruence influences employee citizenship and deviance behaviors. *Journal of Business Ethics*, *170*(1), 147–165. https://doi.org/10.1007/s10551-019-04250-4

Quiyun, G., Liu, W., Zhou, K., & Mao, J. (2020). Leader humility and employee organizational deviance: The role of sense of power and organizational identification. *Leadership & Organization Development*, *41*(3), 463–479. http://dx.doi.org/10.1108/LODJ-07-2019-0287

Rego, A., Cunha, M. P. & Simpson, A. V. (2018). The perceived impact of leaders’ humility on team effectiveness: An empirical study. *Journal of Business Ethics*, *148*(1), 205–218. https://doi.org/10.1007/s10551-015-3008-3

Rego, A., Melo, A. I., Bluhm, D. J., & Júnior, D. R. (2021). Leader-expressed humility predicting team psychological safety: A personality dynamics lens. *Journal of Business Ethics*, *174*(3), 669–686. https://doi.org/10.1007/s10551-020-04622-1

Rego, A., Owens, B., Leal, S., Melo, A. I., e Cunha, M. P., Gonçalves, L., & Ribeiro, P. (2017). How leader humility helps teams to be humbler, psychologically stronger, and more effective: A moderated mediation model. *The Leadership Quarterly*, *28*(5), 639–658. https://doi.org/10.1016/j.leaqua.2017.02.002

Rego, A., Owens, B., Yam, K. C., Bluhm, D., e Cunha, M. P., Silard, A., ... & Liu, W. (2019). Leader humility and team performance: Exploring the mediating mechanisms of team PsyCap and task allocation effectiveness. *Journal of Management*, *45*(3), 1009–1033. https://doi.org/10.1177%2F0149206316688941

Ruffing, E. G., Oleson, D., Tomlinson, J., Park, S. H., & Sandage, S. J. (2021). Humility and relational spirituality as predictors of well-being among Christian seminary students. *Journal of Psychology and Theology*, *49*(4), 419–435. http://dx.doi.org/10.1177/0091647121988968

Shannonhouse, L. R., Mize, M. C. B., Hall, M. E. L., Aten, J. D., Wang, D. C., Davis, E. B., & Van Tongeren, D. R. (2019). The behaviors, benefits, and barriers of humanitarian aid leader humility. *Journal of Psychology and Theology*, *47*(3), 143–159. https://doi.org/10.1177%2F0091647119847539

Spitzer, R. L., Kroenke, K., Williams, J. B. W., & Löwe, B. (2006). A brief measure for assessing generalized anxiety disorder: The GAD-7. *Archives of Internal Medicine, 166*(10), 1092–1097. https://doi.org/10.1001/archinte.166.10.1092

Swain, J. E. (2018). Effects of leader humility on the performance of virtual groups. *Journal of Leadership Studies*, *12*(1), 21–37. https://doi.org/10.1002/jls.21552

Swain, J., & Korenman, L. (2018). In their humble opinion: How expressions of humility affect superiors’ assessments of leadership potential in the US Army. *Military Psychology*, *30*(6), 507–527. https://doi.org/10.1080/08995605.2018.1503002

Tuan, L. T., Rowley, C., Masli, E., Le, V., & Nhi, L. T. P. (2021). Nurturing service-oriented organizational citizenship behavior among tourism employees through leader humility. *Journal of Hospitality and Tourism Management*, *46*, 456–467. https://doi.org/10.1016/j.jhtm.2021.02.001

Van Dyne, L., & LePine, J. A. (1998). Helping and voice extra-role behaviors: Evidence of construct and predictive validity. *Academy of Management Journal, 41*(1), 108–119. https://doi.org/10.2307/256902

Walters, K., & Diab, D. (2016). Humble leadership: Implications for psychological safety and follower engagement. *Journal of Leadership Studies*, *10*(2), 7–18. https://doi.org/10.1002/jls.21434

Wang, J., Zhang, Z., & Jia, M. (2017). Understanding how leader humility enhances employee creativity: The roles of perspective taking and cognitive reappraisal. *Journal of Applied Behavioral Science, 53*(1), 5–31. https://doi.org/10.1177%2F0021886316678907

Wang, L., Owens, B. P., Li, J. (J.), & Shi, L. (2018). Exploring the affective impact, boundary conditions, and antecedents of leader humility. *Journal of Applied Psychology, 103*(9), 1019–1038. https://doi.org/10.1037/apl0000314

Wang, X., Li, H., & Yin, H. (2019). Antecedents and consequences of creativity in teams: When and how leader humility promotes performance via team creativity. *Journal of Creative Behavior, 54*(4), 843–856. https://doi.org/10.1002/jocb.410

Wang, X., Liu, Z., Wen, X., & Xiao, Q. (2022). An implicit leadership theory lens on leader humility and employee outcomes: Examining individual and organizational contingencies. *Tourism Management, 89*, Article 104448. https://doi.org/10.1016/j.tourman.2021.104448

Yang, J., Zhang, W., & Chen, X. (2019). Why do leaders express humility and how does this matter: A rational choice perspective. *Frontiers in Psychology*, Article 1925. https://doi.org/10.3389/fpsyg.2019.01925

Ye, B. H., Tun, V. W. S., Li, J. J., & Zhu, H. (2020). Leader humility, team humility, and employee creative performance: The moderating roles of task dependence and competitive climate. *Tourism Management, 81*, Article 104170. https://doi.org/10.1016/j.tourman.2020.104170

Youssef-Morgan, C. M., & Luthans, F. (2015). Psychological capital and well-being. *Stress and Health, 31*(3), 180–188. https://doi.org/10.1002/smi.2623

Yuan, L., Zhang, L., & Tu, Y. (2018). When a leader is seen as too humble: A curvilinear mediation model linking leader humility to employee creative process engagement. *Leadership & Organization Development Journal*, *39*(4), 468–481. https://doi.org/10.1108/LODJ-03-2017-0056

Zapata, C. P., & Hayes-Jones, L. C. (2019). The consequences of humility for leaders: A double-edged sword. *Organizational Behavior and Human Decision Processes*, *152*, 47–63. https://doi.org/10.1016/j.obhdp.2019.01.006

Zhang, W., & Liu, W. (2019). Leader humility and taking charge: The role of OBSE and leader prototypicality. *Frontiers in Psychology, 10*, Article 2515. https://doi.org/10.3389/fpsyg.2019.02515

Zhong, J., Zhang, L., Li, P., & Zhang, D. Z. (2020). Can leader humility enhance employee wellbeing? The mediating role of employee humility. *Leadership & Organization Development Journal, 41*(1), 19–36. https://doi.org/10.1108/LODJ-03-2019-0124
